# *N*-alkyl and *N*-benzyl indoles are anti-SARS-CoV-2 agents and nsp13 inhibitors

**DOI:** 10.1080/14756366.2025.2539445

**Published:** 2025-08-12

**Authors:** Aurora Albano, Roberta Emmolo, Riccardo De Santis, Elisa Patacchini, Valentina Noemi Madia, Stefania Maloccu, Davide Ialongo, Giuseppe Ruggieri, Merve Arpacioglu, Luigi Scipione, Francesco Saccoliti, Donatella Amatore, Giorgia Grilli, Florigio Lista, Francesca Esposito, Enzo Tramontano, Angela Corona, Roberto Di Santo, Roberta Costi

**Affiliations:** a Dipartimento di Chimica e Tecnologie del Farmaco, Istituto Pasteur-Fondazione Cenci Bolognetti, “Sapienza” Università di Roma, Rome, Italy; b Laboratorio di Virologia Molecolare, Dipartimento di Scienze della Vita e dell’Ambiente Sezione biomedica, Università di Cagliari Cittadella Universitaria di Monserrato, Monserrato, Italy; c Istituto di Scienze Biomediche della Difesa, Roma, Italy; d Dipartimento di Sanità Pubblica e Malattie Infettive, Sapienza, Università di Roma, Rome, Italy; e Dottorato di Interesse Nazionale in One Health approaches to infectious diseases and life science research, Dipartimento di Sanità Pubblica, Medicina Sperimentale e Forense, Università degli Studi di Pavia, Pavia, Italy; f Department of Life Science, Health, and Health Professions, Link Campus University, Rome, Italy

**Keywords:** SARS-CoV-2 inhibition, nsp13, unwinding inhibition, ATPase inhibition, drug discovery

## Abstract

COVID-19 pandemic stimulated tremendous efforts to develop therapeutic strategies targeting SARS-CoV-2, leading to the evaluation of a wide range of potential treatments in clinical trials. However, effective therapeutics remain elusive when the development of new variants and the limits of antiviral drugs is considered. Therefore, the development of antiviral drugs against SARS-CoV-2 is of paramount importance. Among potential drug targets, the SARS-CoV-2 nsp13 is highly attractive thanks to its pivotal role in viral replication. Pursuing our studies on the development of nsp13 inhibitors, in this work we describe the design, synthesis, and biological evaluation of novel inhibitors targeting SARS-CoV-2 nsp13. The newly designed *N*-benzyl indole derivatives were active against both enzymatic activities showing measurable IC_50_ under 30 μM concentration, while *N*-alkyl derivatives showed less promising results. Interestingly, the tested compounds blocked viral replication with no cytotoxicity. Docking studies predicted their binding into an allosteric conserved site located in the RecA2 domain.

## GRAPHICAL ABSTRACT

**Figure:**
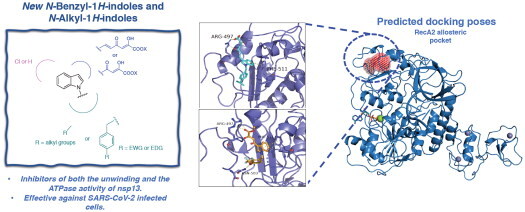

## Introduction

The outbreak of the novel coronavirus (CoV), the severe acute respiratory syndrome coronavirus 2 (SARS-CoV-2), in late 2019 has presented an unprecedented global health challenge^1^. About four years have passed by now, and the Coronavirus Disease 2019 (COVID-19) pandemic has gradually shifted towards its endemic state^2^. Even so, we are still confronting with a sobering reality. To date, the world has witnessed over 7 million confirmed fatalities, with the persistence of long-term COVID cases and approximately 189 000 new infections during the 28-day period, as of December 2024^3^. These ongoing challenges still have a profound impact on the socioeconomic engine of our society. While the vaccination campaign and continuous viral exposure are seen as paths to achieving herd immunity, the ever-evolving spike protein targeted by vaccines^4^, growing campaign weariness, and global disparities in vaccine access create opportunities for the virus to persist and adapt, prolonging its endemic status. For example, the Omicron variant and its sublineages show more than 30 mutations on the spike protein and have proven higher transmissibility and escape of immune elicited by natural infection or vaccines than other variants^5^^,^^6^. Moreover, as history teaches us, the long-term existence of SARS-CoV-2 is likely unavoidable^7^. Consequently, in addition to vaccines, the development of effective antiviral treatments represents a crucial strategy for tackling the ongoing pandemic and potential future outbreaks.

Currently, some small-molecule antivirals targeting two viral proteins, the main protease (M^pro^) or the RNA-dependent RNA polymerase (RdRp), have been approved for therapeutic use in some countries and regions worldwide. For example, the US Food and Drug Administration (FDA) has approved three small-molecule drugs acting as specific antivirals for clinical use, namely Remdesivir, Molnupiravir, and Nirmatrelvir (used in combination with the booster Ritonavir, licenced with the trade name Paxlovid*^™^*)^8^. Along with them, the FDA has also approved an immune modulator (Baricitinib) for certain hospitalised adults with COVID-19. Furthermore, Ensitrelvir has been approved by the Ministry of Health, Labour and Welfare of Japan^9^. China National Medical Products Administration also approved three antivirals, Azvudine, Renmindevir, and Simnotrelvir (used in combination with the booster Ritonavir, licenced with the trade name Xiannuoxin*^®^*)^10^. Of them, Remdesivir^11^, Molnupiravir^12^, Renmindevir^13^, and Azvudine^14^ are inhibitors of the SARS-CoV-2 RdRp, while Nirmatrelvir^15^, Ensitrelvir^16^, and Simnotrelvir^10^ are inhibitors of SARS-CoV-2 M^pro^. However, owing to the necessity for a prompt response to the pandemic, they exhibit varying degrees of shortcomings, i.e. suboptimal potency, toxicity, or poorly adequate pharmacokinetic (PK) properties, including low oral drug exposure, poor oral bioavailability, and moderate stability in human liver microsomes^10^^,^^12–15^. Moreover, drug resistant variants *vs* currently approved drugs have already emerged. For example, variants bearing E166N/V, M165T, G143S, Q189E, A173V, H172F/Q/Y, or Q192S/T/V mutations in M^pro^ have been reported to be resistant to Nirmatrelvir^17–19^. Therefore, developing next-generation effective antivirals is of paramount importance.

Mutational analysis of the current SARS-CoV-2 and comparison of its genome to previous CoVs has revealed alternative targets that exhibit significantly lower mutation rates and higher structural conservation^20^. These include functional proteins that can be targeted with small-molecule inhibitors, which, in contrast to vaccines, offer distinct advantages in terms of ease of formulation, production, storage, and administration. Notably, among these candidates, the viral non-structural protein 13 (nsp13) demonstrates a 99.8% of sequence identity^21^^,^^22^ when comparing SARS-CoV-1 and SARS-CoV-2, with only a single amino acid out 601 of difference between nsp13s of SARS-CoV (I570) and SARS-CoV-2 (V570), making it a highly attractive target for drug development. Moreover, nsp13 is an enzyme that plays a pivotal role in viral RNA synthesis and unwinding. The CoVs nsp13 belongs to the 1B (SF1B) helicase superfamily that can target natural nucleotides as substrates when performing its ATPase activity. Nsp13, in fact, utilises the energy of nucleotide triphosphate hydrolysis to catalyse the unwinding of double-stranded DNA or RNA in a 5′ to 3′ direction^23^. As all SF1 helicases, nsp13 shows two canonical RecA ATPase domains (RecA1 and RecA2)^24^, coupled with three domains unique to nidovirus helicases: the N-terminal zinc-binding domain (ZBD), essential for the helicase activity, a stalk domain (SD), and a 1B domain^25^^,^^26^. The helicase function of SARS-CoV-2 is regarded as crucial for viral replication, as it has been demonstrated to be indispensable for other coronaviruses, including the nidovirus equine arteritis virus^27^ and the beta-coronavirus murine hepatitis virus^28^. Consequently, nsp13 serves as a vital enzyme for viral replication and represents a validated target for drug discovery, whose inhibition offers a promising avenue for the development of antiviral agents.

This work delves into the design, synthesis, and biological evaluation of novel inhibitors targeting nsp13, aiming to shed light on the development of therapeutic agents capable of mitigating the impact of SARS-CoV-2.

**Figure 1.**
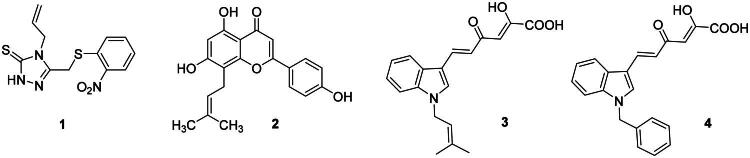
Structures of nsp13 inhibitors.

Very recently, SARS-CoV-2 nsp13 has been actively explored as drug target, with several reports describing small molecules as inhibitors of SARS-CoV-2 nsp13^29–32^, both of synthetic (as for SSYA10-001^31^, **1**, Figure 1) or natural origin, such as in the case of licoflavone C (**2**, Figure 1)^29^. Even so, most of them showed selective inhibition of nsp13-associated unwinding activity in respect to the ATPase one, and/or weak activity or inactivity *vs* SARS-CoV-2 infected cells. SSYA10-001, for example, was one of the first SARS-CoV-2 nsp13 inhibitors described, showing IC_50_ *vs* unwinding of 7.5 µM, even though reporting a weak antiviral activity (EC_50_ = 81 µM)^31^. Similarly, the naturally occurring flavonoid **2** was shown to allosterically block both SARS-CoV-2 nps13-associated activities at micromolar concentrations (IC_50_ of 9.9 and 18.3 µM against unwinding and ATPase, respectively) but without exerting antiviral activity (EC_50_ > 100 µM). Very recently, we described the first report of inhibitors active against both the SARS-CoV-2 nsp13-associated activities and capable also of blocking viral replication in SARS-CoV-2 infected cells^33^. In particular, we found a small set of indolyl diketo acid (DKA) derivatives endowed with low micromolar inhibitory activity against both the unwinding and ATPase. Among them, compounds **3** and **4** (Figure 1) were shown as promising hit compounds (**3**: IC_50_ unwinding = 9.51 µM, IC_50_ ATPase = 26.8 µM, EC_50_ = 4.54 µM, CC_50_ > 264 µM; **4**: IC_50_ unwinding = 12.69 µM, IC_50_ ATPase = 12.8 µM, EC_50_ = 1.03 µM, CC_50_ > 264 µM). Furthermore, both compounds proved to be active also on other human CoVs such as HCoV229E and MERS-CoV in the low micromolar/submicromolar range (**3**, EC_50_ = 0.33 µM on MERS-CoV and EC_50_ = 0.54 µM on HCoV229E; **4**, EC_50_ = 6.32 µM on MERS-CoV and EC_50_ = 1.83 µM on HCoV229E). The experimental investigation of the binding mode revealed ATP-non-competitive kinetics of inhibition, not affected by substrate-displacement effect, suggesting an allosteric binding mode that was further supported by molecular modelling calculations predicting the binding into an allosteric conserved site located in the RecA2 domain.

**Figure 2.**
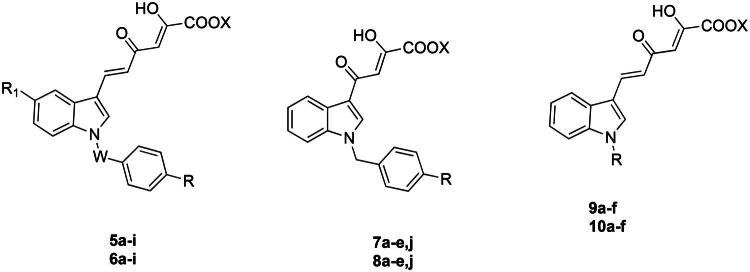
Structures of derivatives **5a − i, 6a − i, 7a − e,j, 8a − e,j, 9a − f,** and **10a − f**.

In the light of these successful results, we decided to pursue our studies within this new class of nsp13 inhibitors. Therefore, we designed and synthesised new indolyl DKA derivatives structurally related to **3** and **4** with the aim of deepening the structure-activity relationships (SARs) within this series of inhibitors. In detail, by keeping fixed the 3-diketo acid indole moiety, we conceived new series of derivatives (**5a − i, 6a − i, 7a − e,j, 8a − e,j, 9a − f** and **10a − f**) characterised by one or more of the following modifications: introduction of variously substituted benzyl or alkyl groups on the nitrogen atom of the indole ring, and/or shortening of the diketohexenoic branch into a diketobutanoic one. Depending on the substituent in 1-position of the indole core, the so obtained derivatives can be, therefore, mainly grouped in: (i) *N*-benzyl diketohexenoic (series **5** and **6**) and diketobutanoic (series **7** and **8**) derivatives, structurally related to **4**, characterised by various substituents endowed with different steric or electronic properties in position 4 of the benzyl ring; (ii) *N*-alkyl diketohexenoic derivatives (series **9** and **10**), structurally related to **3**^33^, in turn grouped in sub-series of unsaturated and saturated derivatives (Figure 2).

## Materials and methods

### Chemistry

#### General instrumentation

Melting points were determined on a Bobby Stuart Scientific SMP1 melting point apparatus and are uncorrected. Compound purity was always >95% as determined by combustion analysis. Analytical results agreed to within ±0.40% of the theoretical values. Infra-red (IR) spectra were recorded on a PerkinElmer Spectrum-One spectrophotometer.^1^H NMR and ^13^C NMR spectra were recorded at 400 MHz and 100 MHz, respectively, on a Bruker AC 400 Ultrashield 10 spectrometer (400 MHz); the following abbreviations were used: s for singlet, bs for broad singlet, d for doublet, t for triplet, dd for double doublet, m for multiplet; chemical shift are given in d with respect to the residual solvent signal, coupling constant are given in Hz. Dimethyl sulfoxide-*d*_6_ 99.9% (CAS 2206–27-1) and deuteromethanol-*d*_4_ 98.8% (CAS 811–98-3) of isotopic purity (Aldrich) were used. Mass spectra were recorded on a ThermoFinnigan LCQ Classic LC/MS/MS ion trap equipped with an ESI source and a syringe pump. Samples (1 0 ^−4^ − 1 0 ^−5^ M in MeOH/H_2_O 90:10) were infused in the electrospray system at a flow rate of 5 − 10 μL min^−1^. Column chromatography was performed on silica gel (Merck, 70 − 230 mesh) or alumina (Merck, 70 − 230 mesh). All compounds were routinely checked on TLC by using aluminium-baked silica gel plates (Merck silica-gel 60 F_254_ plates). Developed plates were visualised by UV light. Solvents and reagent were of analytical-grade and, when necessary, were purified and dried by standard methods. Concentration of solutions after reactions and extractions involved the use of rotary evaporator (Büchi) operating at a reduced pressure (ca. 20 Torr). Organic solutions were dried over anhydrous sodium sulphate (Merck). All solvents were freshly distilled under nitrogen and stored over molecular sieves for at least 3 h prior to use.

#### General experimental procedures

**General Procedure A (GP-A) to Obtain *N*-Benzyl Indoles (11b,c,e, 12f** and **13a–e,j)**. To a solution of 1*H*-indole-3-carboxaldehyde (for compounds **11b,c,e,**) or 3-acetylindole (for compounds **13a–e,j**) or (*E*)-4-(1*H*-indol-3-yl)but-3-en-2-one (for compound **12f**) (27.6 mmol) in anhydrous DMF (30 ml), *t*-BuOK (33.1 mmol) was added at 0 °C. After stirring at room temperature for 10 min, the proper benzyl bromide (33.1 mmol) or benzenesulphonyl chloride (for compound **12f**) (35.9 mmol) was added at 0 °C. Then, the resulting mixture was stirred at room temperature for the proper time and monitored by TLC. The reaction was diluted with water (40 ml) and acidified with 1 N HCl up to pH 4. The solid that formed was filtered under vacuum, washed with water and light petroleum ether to afford the pure compounds. For compound **12f**, the reaction was quenched with water (90 ml), acidified up to pH 4 with 1 N HCl, and extracted with ethyl acetate (3 × 100 ml). The combined organic layers were washed with brine (200 ml), dried over Na_2_SO_4_, concentrated, and purified by column chromatography on silica gel eluting with *n*-hexane/ethyl acetate 30:70. For each compound proper benzyl bromide; reaction time; recrystallization solvent; yield (%); melting point (°C); IR; ^1^H NMR and elemental analysis are reported.

**General procedure B (GP-B) to Obtain α,β-Unsaturated Ketones (12b,c,e,** and **15d)**. The proper indole carboxaldehyde (8.5 mmol) was dissolved in 29 ml of acetone. To this mixture was added 5 N NaOH (12.4 ml), and the mixture was stirred at 50 °C for 24 h. After this period water (300 ml) was added and the solid that formed was filtrated under vacuum, washed with water and light petroleum ether to obtain pure compounds for compounds **12b, 12c, 12e** or purified via column chromatography (SiO_2_) to obtain pure derivatives. For each compound proper indole carboxaldehyde; chromatography eluent; recrystallization solvent; yield (%); melting point (°C); IR;^1^H NMR and elemental analysis are reported.

**General procedure C (GP-C) to Obtain Diketo Esters (6b,c,e,f, 8a–e,j,** and **10d)**. For compounds **6b,c,e,f** and **10d**, freshly prepared sodium ethoxide (7.2 mmol), obtained by the dissolution of Na (72 mmol) in 7.47 ml of absolute ethanol, was added to a well-stirred solution of the proper α,β-unsaturated ketone derivative (18 mmol) and diethyl oxalate (72 mmol) in anhydrous THF (4 ml) under argon atmosphere^34^.

For compounds **8a–e,j**, freshly prepared sodium ethoxide (34 mmol), obtained as above, was added to a well-stirred solution of the proper acetyl derivative (17 mmol) in anhydrous THF (3.8 ml) under argon atmosphere; afterwards, diethyl oxalate (34 mmol) was added.

The mixture was stirred at room temperature for the proper time under argon atmosphere and then was poured into *n*-hexane (200 ml). The resulting precipitate was vigorously stirred for 30 min in 1 N HCl (200 ml). The solid that formed was filtered, washed with water and light petroleum ether, and dried under IR lamp to afford the pure diketo esters. For each compound proper aldehyde or acetyl derivative; reaction time; recrystallization solvent; yield (%); melting point (°C); IR;^1^H NMR and elemental analysis are reported.^1^H NMR and ^13^C NMR spectra are reported in Supporting Information.

**General procedure D (GP-D) to Obtain Diketo Acids (5b,c,e,f, 7a–e,j,** and **9d)**. A solution of 1 N NaOH (11.9 mmol) was added to a solution of the appropriate ester (2.39 mmol) in 1:1 THF/methanol (54 ml) and the reaction was stirred vigorously at room temperature for the proper time. The organic phase was removed under vacuum and to the resulting suspension crushed ice was added. The mixture was acidified with 1 N HCl up to pH 4–5 and the solid that formed was filtered, washed with water and light petroleum ether, and dried under IR lamp (or extracted with ethyl acetate, dried over Na_2_SO_4_, filtered and evaporated under reduced pressure for derivatives **5c,e** and **7a-c,e**) to afford pure acids. For each compound appropriate ester; reaction time; recrystallization solvent; yield (%); melting point (°C); IR; ^1^H NMR and elemental analysis are reported. ^1^H NMR, ^13^C NMR and MS (ESI) spectra are reported in Supporting Information.

#### Specific experimental procedures and characterization

Specific experimental procedures and characterisation of compounds **5a − i, 6a − i, 7a − e,j, 8a − e,j, 9a − f, 10a − f, 11b,c,e, 12b,c,e,f, 13a − e,j, 14d** and **15d** are reported in Supplementary Material.

FTIR, ^1^H NMR, ^13^C NMR and MS (ESI) spectra for the newly synthesised compounds **5b,c,e,f, 6b,c,e,f, 7a − c,e, 8a − c,e, 9d** and **10d** are reported in (Supplementary Materials Figures S4–S75).

### Biological assays

#### SARS-CoV-2 nsp13 expression and purification

SARS-CoV-2 nsp13 was expressed from pNIC-ZB vector (addgene 159614)^35^ following the procedure described in reference^33^. Briefly, the protein was expressed in *E. coli* BL21 Rosetta 2 cells in Terrific Broth media induced with 300 µM IPTG and lasted overnight at 18 °C at 200 rpm.

Cell pellets were resuspended and sonicated for 15 min, 10s on 5s off, and clarified by centrifugation at 11 000× g for 50 min. The supernatant was used for a batch binding of 40 min with 3 ml of Ni-sepharose (Cytiva). Beads were loaded on a gravity flow column and washed with 50 ml lysis buffer, 25 ml wash buffer (50 mM HEPES pH 7.5, 500 mM NaCl, 5% Glycerol, 45 mM Imidazole, 0.5 mM TCEP), 10 ml Hi-salt buffer (50 mM HEPES pH 7.5, 1 M NaCl, 5% Glycerol, 0.5 mM TCEP) and again with 10 ml of wash buffer. The protein was eluted with elution buffer (50 mM HEPES pH 7.5, 500 mM NaCl, 5% Glycerol, 300 mM Imidazole, 0.5 mM TCEP). The eluted fraction was immediately applied to a 5 ml Hi-Trap SP HP column using a syringe. The column was washed with 20 ml elution buffer and proteins were eluted with 20 ml Hi-salt buffer (20 fractions 0–100% Hi-salt buffer). Protein fractions were pooled and loaded into a Superdex 200 10/300 GL column equilibrated in 50 mM Hepes, 500 mM NaCl, 5% Glycerol and 0.5 mM TCEP. The elution fractions were analysed for purity by SDS page. Proteins were stored at −80 °C.

#### Determination of SARS-CoV-2 nsp13 unwinding-associated activity

The SARS-CoV-2 nsp13 unwinding-associated activity was measured as reported^29^ in black 384 well plates (PerkinElmer), in 20 μl reaction volume containing 20 mM Tris–HCl pH 7.2, 50 mM NaCl, 2 μM Hel Capture oligo (5′- TGG TGC TCG AAC AGT GAC −3′) from Biomers, 5 mM MgCl_2_, 10 µg/mL BSA and 180 µM TCEP, 5% dimethyl sulfoxide (DMSO) or inhibitor and 2 nM of purified nsp13. The reaction mixture containing the enzyme was pre-incubated for 10 min with inhibitor at room temperature (RT). The reaction was started adding 500 µM ATP and 500 nM annealed DNA substrate (5′- AGT CTT CTC CTG GTG CTC GAA CAG TGA C-Cy3-3′, 5′- BHQ-2-GTC ACT GTT CGA GCA CCA CCT CTT CTG A-3′) from Biomers. After 15 min of incubation at 37 °C, fluorescence products were measured with Victor Nivo (Perkin) at 530/580 nm.

#### Determination of SARS-CoV-2 nsp13 ATPase-associated activity

The SARS-CoV-2 nsp13 ATPase-associated activity was measured as reported^29^ in a transparent 96 well plate (PerkinElmer), in 25 μl reaction volume containing 20 mM Tris–HCl pH 7.2, 50 mM NaCl, 2 mM MgCl_2_, 10 µg/mL BSA and 180 µM TCEP, 5% DMSO or inhibitor and 25 nM of purified nsp13. The reaction was started adding 200 µM ATP. After 30 min of incubation at 37 °C, 50 µl of Biomol^®^ Green Reagent (Prod. No. BML-AK111, Enzo Lifescience) were added and reaction was incubated for 10 min at RT, protected from the light. Products were measured with Victor Nivo (Perkin) at 650 nm for ABS value.

#### Data analysis

The EC_50_ value was determined and graphed by GraphPad Prism v9.0 software by fitting a variable slope-sigmoidal dose-response curve.

#### Virus production

A virus working stock was prepared through the propagation in Vero E6 cells cultured in minimum essential medium (MEM) containing 2% (w/v) foetal bovine serum (Euroclone S.p.A.) of a strain of SARS-CoV-2 variant omicron XBB.1.16.11 (hCoV-19/Italy/LAZ-DIBS-230829301/2023) isolated from nasopharyngeal swab by Defence Institute for Biomedical Sciences, Rome Italy. 72 h after the infection, supernatants containing the released viral particles were collected and centrifuged at 600 g for 5 min. Virus stocks were kept at −80 °C until use. The viral titre was determined by plaque assay. The swab was accompanied by signed informed consent.

#### SARS-CoV-2 plaque assay

The Vero cell line used in this work was Vero E6, a fibroblast-like cell line isolated from the kidney of an African green monkey. Vero E6 was acquired in 2016 from Sigma-Aldrich (catalog no. 85020206).

Confluent Vero E6 cell monolayers were infected for 1 h at 37 ◦C with SARS-CoV-2 (0.01 MOI). Then, after 1 h incubation, the inoculum was replaced with fresh medium supplemented with 2% FBS and containing respectively each indole derivative at different concentrations. Untreated-infected cells were used as positive control of viral infection. After 24 h, the supernatants were collected, and plaque assay was performed. Briefly, Vero E6 cells seeded in a 12/24-well plate for 1 h at 37 °C. Then, the inoculum was removed, and the medium was replaced with a mixture of MEM (no glutamine, no phenol-red-GIBCO), 1.5% Tragacanth (SIGMA), NaHCO_3_ 7% (Gibco), L-glutamine 1X (Gibco), MEM NEAA 1x (Gibco), 0.02 M Hepes (Euroclone), DMSO (Sigma-Aldrich) and 2% FBS (final concentration). Three days post-infection the mixture was carefully removed, plates were washed with saline solution, stained with 1% crystal violet for 10 min and plaque forming units (PFU) were counted. The plaque reduction ratio was calculated as (100− N/N0 × 100) where N is the PFU count of the treated sample, and N0 is the PFU count of the control sample. The plaque assay was also used to determine the concentration of molecules that inhibits 50% of viral titre in each well (IC_50_).

#### Cellular toxicity assay

Vero E6-green fluorescent protein (GFP) cells (Janssen Pharmaceutical were maintained in Dulbecco’s modified Eagle’s medium (DMEM, Gibco) supplemented with 10% v/v foetal beef serum (FBS, Gibco), 0.075% Sodium Bicarbonate (7.5% solution, Gibco) and 1X Pen-strep (Euroclone) and kept under 5% CO_2_ on 37 ◦C. The cells were seeded at 10 000 cells/well in 96-well black cell-treated plates (PerkinElmer). The following day, cells were incubated with the control compounds at different concentrations. Compound was dissolved in 0.1% DMSO. Seventy-two hours post infection the GPF signal, as direct index of cellular viability, was quantified by measuring the total-well fluorescence with a Victor 3 multiplate reader (PerkinElmer) set to excitation and emission wavelengths of 485 and 535 nm, respectively.

The percentage of induced cytopathic effects (CPE) was calculated considering the mock as 100% of cell viability, and the readout from empty wells as blank. The compound half-maximal cytopathic concentration (CC_50_), was determined via non-linear regression by using on Prism 9 v. 9.4.1 software (GraphPad) the built-in function dose-response inhibition, log-concentration-normalized response. Experimental points represent the average and standard deviation of at least two sets of independent triplicates.

### Molecular docking protocol

The 3D model of the SARS-CoV-2 nsp13 protein was generated as previously reported^33^. Briefly, the 3D model of the nsp13 protein of SARS-CoV-2 adopted here is the SwissModel generated with the SARS-CoV helicase (PDB: 6JYT). The mentioned complex reports the natural ligands: adenosine diphosphate (ADP) and single-stranded RNA (ssRNA). The nsp13 3D structure has been prepared using the Protein Preparation Wizard of Schrodinger. Before utilising these receptor structures in docking calculations, preparation steps were taken using the Protein Preparation Wizard utility within the Maestro software package^36^. The receptor structures underwent preparation, including assigning bond orders, adding hydrogens, and generating physiological pH states using the EPIK tool. Subsequently, the “Minimize and Delete Waters” tool was employed to minimise overall protein structures, with restrained heavy atoms and removal of all water molecules.

After generating the model, docking calculations were attained for **5c**, **5e**, **5g**, **5i**, **8d** and **8e** employing the Glide tool implemented in Maestro^37^. To prepare all the ligands for docking calculations, the Schrodinger software suite’s “LigPrep” tool was employed. This included adding hydrogen atoms, generating all tautomeric states, and retaining specified chiralities. The docking grid boxes were centred on RecA2 domain based on previously reported DKA inhibitors (centre in −34.42 × 1.69 × −21.5) with a grid box dimension equal to 20 Å × 20 Å × 20 Å. Finally, docking runs were carried out using the standard Glide protocol with a rigid treatment of the protein, employing standard settings. The best-scoring complexes in terms of GlideScore were selected.

All the figures were rendered with Pymol^38^.

### Molecular dynamics simulations protocol

The molecular dynamics (MD) simulations were based on the **5g**-nsp13 structure by means of the Desmond module of the Schrödinger software package^39^. The System Builder panel was used to prepare the system for the MD calculation. It was solvated in a orthorombic water box with a buffer distances of 10 Å, using as a solvent model the predefined option (TIP3P). The initial +3 positive charge was neutralised using 3 Cl^-^ ions. The salt concentration was set to 0.15 M NaCl. The OPLS4 force field was used for the constructed receptor/ligand/membrane system. Then, the system was relaxed and then analysed employing the NPγT ensemble simulation with increasing temperature from 100 to 300 K at 1 bar pressure for 150 ns.

## Results and discussion

### Chemistry

**Scheme 1.**
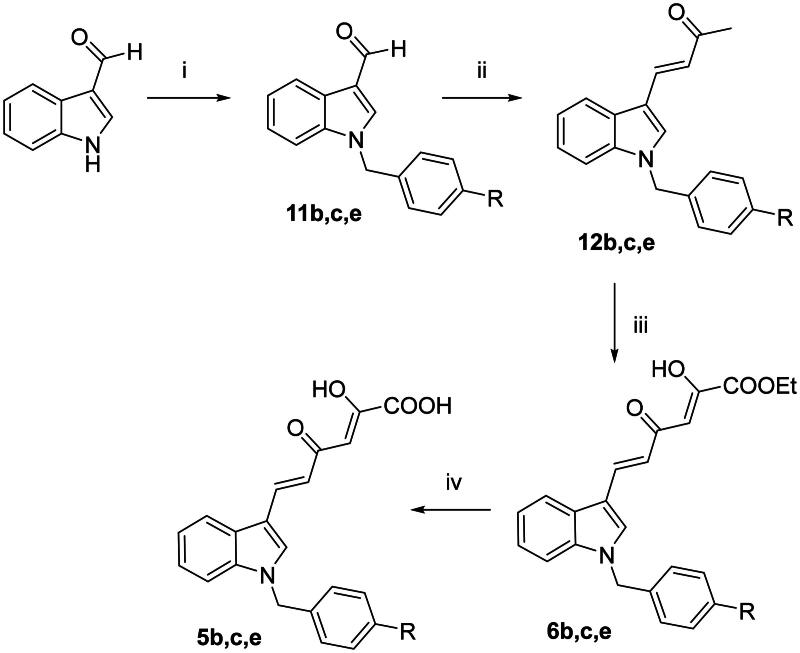
Synthetic route to **5b,c,e** and **6b,c,e** derivatives. Reagents and conditions: (i) proper benzyl bromide, *t*-BuOK, DMF dry, 0 °C to room temp, 30 min–15 h, 74–95% yield; (ii) acetone, 5 N NaOH, 50 °C, 24 h, 72–100% yield; (iii) diethyl oxalate, EtONa, THF dry, N_2_, room temp, 20–45 min, 63–95% yield; and (iv) 1 N NaOH, 1:1 THF/EtOH, room temp, 15–30 min, 64–91% yield.

Compounds **5a,d,g-i** and **6a,d,g − i** were obtained as previously reported^40^. *N*-benzyl diketohexenoic derivatives **5b,c,e** and **6b,c,e** were synthesised as reported in Scheme 1. Commercially available 1*H*-indole-3-carboxaldehyde underwent a nucleophilic substitution reaction with the appropriate benzyl bromide using potassium *tert*-butoxide as a base to give *N*-benzyl derivatives **11b,c,e**. Afterwards, a crossed aldol condensation reaction with acetone in the presence of 5 N NaOH furnished the enones **12b,c,e** that underwent a Claisen-Schmidt condensation with diethyl oxalate using freshly prepared sodium ethoxide as base, to give dikehexenoic ethyl esters **6b,c,e**. The subsequent base-catalyzed hydrolysis using 1 N NaOH afforded the corresponding acids **5b,c,e**.

**Scheme 2.**
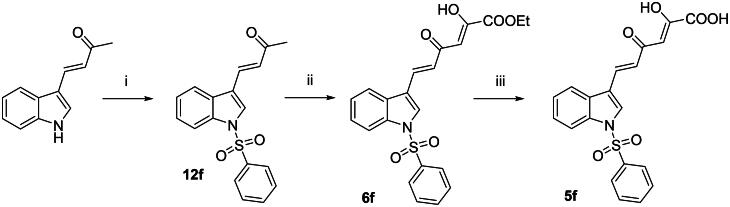
Synthetic route to **5f** and **6f** derivatives. Reagents and conditions: (i) benzenesulphonyl chloride, *t*-BuOK, DMF dry, 0 °C to room temp, 1 h, 28% yield; (ii) diethyl oxalate, EtONa, THF dry, N_2_, room temp, 2 h, 58% yield; and (iv) 1 N NaOH, 1:1 THF/EtOH, room temp, 30 min, 75% yield.

The sulphonyl derivatives **5f** and **6f** were synthesised as reported in Scheme 2. By reaction of (*E*)-4-(1*H*-indol-3-yl)but-3-en-2-one^41^^,^^42^ with benzenesulphonyl chloride in presence of potassium *tert*-butoxide, the *N*-sulphonyl derivative **12f** was obtained. The subsequent Claisen-Schmidt condensation and alkaline hydrolysis gave compounds **6f** and **5f**, respectively, in a similar fashion to the previous scheme. It is worthy of note that several attempts have been made in order to synthesise the diketobutanoic analogues of **5f** and **6f**, but no compound except of the NH derivative **11f** was obtained. Given that the benzenesulphonyl ring is a good leaving group, we speculated that the presence of strong bases as NaOEt could affect the nitrogen-sulphur bond when an electron-withdrawing group (an acetyl group in our case) is present in three-position of the indole. Conversely, the presence of a α,β-unsaturated chain in three-position of the indole ring, might stabilise the overall structure, preventing the desulphonylation reaction.

**Scheme 3.**
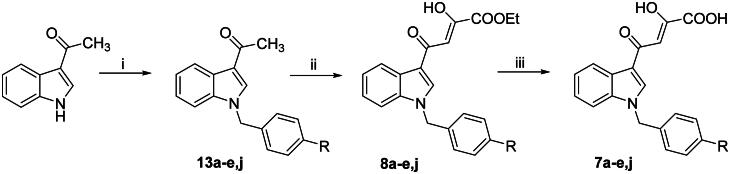
Synthetic route to **7a − e,j,** and **8a − e,j** Derivatives. Reagents and conditions: (i) proper benzyl bromide, *t*-BuOK, DMF dry, 0 °C to room temp, 30 min–15 h, 79–100% yield; (ii) diethyl oxalate, EtONa, THF dry, N_2_, room temp, 45 min–2.5 h 40–97% yield; and (iii) 1 N NaOH, 1:1 THF/EtOH, room temp, 15 min–1 h, 48–87% yield.

*N*-benzyl diketobutanoic derivatives **7a − e,j** and **8a − e,j** were synthesised as described in Scheme 3. Commercially available 3-acetylindole was reacted with the appropriate benzyl bromide in a similar fashion to that described in Scheme 1, obtaining *N*-benzyl derivatives **13a − e,j** that were subsequently converted into the corresponding diketo esters **8a − e,j** *via* a Claisen-Schmidt condensation in the same conditions described above. Finally, an alkaline hydrolysis gave the corresponding acids **7a − e,j**.

**Scheme 4.**
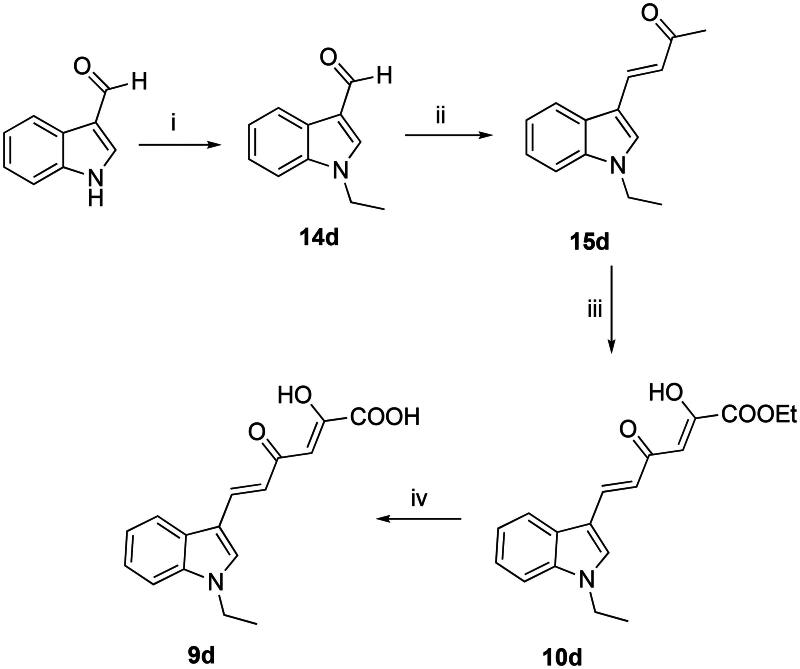
Synthetic route to **9d** and **10d** derivatives. Reagents and conditions: (i) iodoethane, NaH 60%, DMF dry, room temp, 1 h, 77% yield; (ii) acetone, 5 N NaOH, 50 °C, 24 h, 84% yield; (iii) diethyl oxalate, EtONa, THF dry, N_2_, room temp, 1.5 h, 61% yield; and (iv) 1 N NaOH, 1:1 THF/MeOH, room temp, 1 h, 72% yield.

Compounds **9a − c,e,f** and **10a − c,e,f** have been obtained as previously reported by us^40^. The synthesis of *N*-alkyl diketohexenoic derivatives **9d** and **10d** was performed as reported in Scheme 4. Also for these derivatives the synthetic route resembles that of Scheme 1.

### Evaluation of biological activities

#### In vitro screening for nsp13 inhibitory activity

All compounds **5a − i, 6a − i, 7a − e,j, 8a − e,j, 9a − f** and **10a − f** were tested *in vitro* on both the SARS-CoV-2 nsp13 unwinding and ATPase associated activities (Table 1), using compound **4** as positive control^33^.

**Table 1.** Inhibition of SARS-CoV-2 nsp13-associated activities by the newly synthesised compounds **5a − i, 6a − i, 7a − e,j, 8a − e,j, 9a − f,** and **10a − f**.

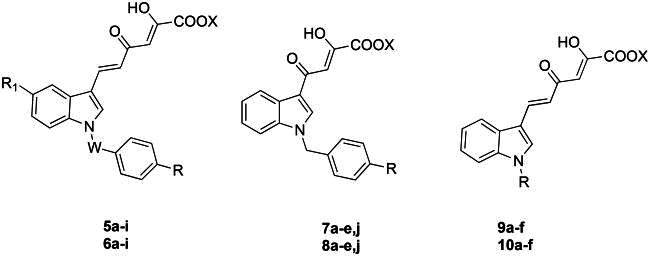						
Cpd	R	R_1_	W	X	Activity in enzyme assay IC_50_ (µM)^a^	
					Unwinding BSA-TCEP^b^	ATPase BSA-TCEP^c^
**5a**	OCH_3_	H	CH_2_	H	10.47 ± 0.81	19.92 ± 0.73
**5b**	CH_3_	H	CH_2_	H	13.56 ± 1.96	3.83 ± 0.45
**5c**	*i*-Pr	H	CH_2_	H	4.73 ± 0.50	1.8 ± 0.19
**5d**	F	H	CH_2_	H	7.63 ± 2.48	9.91 ± 0.5
**5e**	OCF_3_	H	CH_2_	H	6.24 ± 0.24	6.41 ± 0.05
**5f**	H	H	SO_2_	H	7.54 ± 0.76	5.36 ± 0.74
**5 g**	CN	H	CH_2_	H	2.08 ± 0.04	1.91 ± 0.31
**5h**	OH	H	CH_2_	H	2.77 ± 0.42	9.1 ± 3.4
**5i**	F	Cl	CH_2_	H	0.47 ± 0.01	26.35 ± 2.52
**6a**	OCH_3_	H	CH_2_	Et	6.65 ± 1.20	2.21 ± 0.26
**6b**	CH_3_	H	CH_2_	Et	7.34 ± 1.81	2.88 ± 0.59
**6c**	*i*-Pr	H	CH_2_	Et	5.81 ± 0.47	5.80 ± 0.12
**6d**	F	H	CH_2_	Et	3.64 ± 0.11	3.22 ± 0.14
**6e**	OCF_3_	H	CH_2_	Et	5.66 ± 0.21	2.17 ± 0.26
**6f**	H	H	SO_2_	Et	11.025 ± 0.37	7.075 ± 0.74
**6 g**	CN	H	CH_2_	Et	6.76 ± 1.18	6.24 ± 0.24
**6h**	OH	H	CH_2_	Et	15.4 ± 1.47	3.70 ± 0.74
**6i**	F	Cl	CH_2_	Et	5.22 ± 0.44	3.92 ± 0.96
**7a**	OCH_3_	–	–	H	>30 (50.41% ^d^)	>30 (73.40% ^d^)
**7b**	CH_3_	–	–	H	20.62 ± 3.22	8.33 ± 0.45
**7c**	*i*-Pr	–	–	H	14.12 ± 0.48	13.01 ± 1.08
**7d**	F	–	–	H	>30 (50 %)	>30 (70 %)
**7e**	OCF_3_	–	–	H	22.23 ± 1.37	20.335 ± 1.35
**7j**	H	–	–	H	30.0 ± 1.50	10.3 ± 1.75
**8a**	OCH_3_	–	–	Et	13.12 ± 1.78	8.06 ± 1.78
**8b**	CH_3_	–	–	Et	13.18 ± 2.76	7.01 ± 0.05
**8c**	*i*-Pr	–	–	Et	9.66 ± 2.89	9.64 ± 2.27
**8d**	F	–	–	Et	1.73 ± 0.53	8.7 ± 0.53
**8e**	OCF_3_	–	–	Et	11.29 ± 0.65	2.29 ± 0.02
**8j**	H	–	–	Et	26.02 ± 0.89	14.53 ± 1.49
**9a**	CH_2_CH = CHCH_3_	–	–	H	19.4 ± 3.7	>30 (70%)
**9b**	CH_2_C(CH_3_)=CH_2_	–	–	H	>30 (72%)	>30 (68%)
**9c**	CH = C(CH_3_)_2_	–	–	H	1.56 ± 0.16	>30 (98.61%)
**9d**	CH_2_CH_3_	–	–	H	11.80 ± 4.03	8.32 ± 0.08
**9e**	CH_2_CH_2_CH_2_CH_3_	–	–	H	>30 (41%)	>30 (100%)
**9f**	CH_2_CH_2_CH(CH_3_)_2_	–	–	H	15.94 ± 0.59	23.11 ± 1.41
**10a**	CH_2_CH = CHCH_3_	–	–	Et	13.21 ± 1.86	8.79 ± 0.69
**10b**	CH_2_C(CH_3_)=CH_2_	–	–	Et	21.46 ± 0.23	7.26 ± 0.65
**10c**	CH = C(CH_3_)_2_	–	–	Et	19.27 ± 0.76	4.93 ± 1.4
**10d**	CH_2_CH_3_	–	–	Et	11.85 ± 4.03	18.51 ± 0.65
**10e**	CH_2_CH_2_CH_2_CH_3_	–	–	Et	14.79 ± 1.18	5.72 ± 1.63
**10f**	CH_2_CH_2_CH(CH_3_)_2_	–	–	Et	13.5 ± 0.25	6.27 ± 0.2
**4** ^e^	–	–	–	–	12.69 ± 4.9	12.8 ± 1.3

^a^Inhibitory concentration 50% (*μ*M) determined from dose-response curves.

^b^Experiments performed against SARS-CoV-2 nsp13-associated unwinding activity.

^c^Experiments performed against SARS-CoV-2 nsp13-associated ATPase activity.

^d^Percentage of enzymatic residual activity measured at 30 µM.

^e^From reference^33^.

In general, the newly designed indolyl derivatives of series **5 − 8** were proven active against both the enzymatic activities showing measurable IC_50_ under 30 μM concentration, with only 2 out of 30 tested compounds inactive *vs* both unwinding and ATPase, only 1 active at 30 μM against unwinding and no compound inactive against ATPase activity. Among them, 1 compound proved to be active in the submicromolar range and 8 derivatives showing inhibitory potencies about or lower than 5 μM against unwinding, while 12 compounds showed IC_50_ values about or lower than 5 μM *vs* ATPase (out of the 30 compounds tested). Derivatives of series **9** and **10** proved to be less promising, with 3 out of 14 tested compounds inactive *vs* both unwinding and ATPase, no one inactive against unwinding and 2 inactive against ATPase activity.

Overall, *N*-benzyl diketohexenoic derivatives (series **5** and **6**) were found to be good dual inhibitors of both the nsp13-associated activities with highly promising inhibitory potencies against both the unwinding and ATPase activities. Regarding the unwinding, we can observe that all derivatives of this series showed IC_50_ values between 0.47 μM (**5i**) and 15.4 μM (**6h**). More in detail, 5 derivatives (**5c,g-i** and **6d**) out of 18 tested showed high inhibitory activities, with IC_50_ values lower than 5 μM, 10 compounds (**5a,d,e,f** and **6a − c,e,g,i**) proved to be active with 5 μM < IC_50_ < 10, and only 4 (**5b** and **6f,h**) reported inhibitory activity with IC_50_ values > 10 μM. As concerns ATPase, all the compounds of this series were active up to the tested concentrations, with IC_50_s in the range 1.8 (**5c**) **− 26.35** μM (**5i**). Particularly, 9 derivatives (**5b,c,g** and **6a,b,d,e,h,i**) out of 18 tested showed high inhibitory activities, with IC_50_ values lower than 5 μM, 7 compounds (**5d-f,h** and **6c,f,g**) proved to be active with 5 μM < IC_50_ < 10, and only 2 (**5a,i**) reported inhibitory activity with IC_50_ values > 10 μM. Notably, the most active compound against unwinding was derivative **5i** showing IC_50_ of 0.47 μM, the best acting compounds *vs* ATPase were **5c** and **5 g** (IC_50_ of 1.8 and 1.91 μM, respectively), that proved to be also the best dual inhibitors along with compound **6d** (IC_50_ unwinding = 3.64 μM, IC_50_ ATPase = 3.22 μM). In the light of this, we can state that *N*-benzyl diketohexenoic derivatives are generally more active against ATPase than unwinding, even though the best IC_50_ value was observed for the unwinding inhibition (**5i**, IC_50_ = 0.47 μM). It is also possible to notice that the ester derivatives were, overall, more active than the acid counterparts, although notable exceptions can be cited like the case of acids **5f** and **5g** being more active than the ester counterparts and for the couples **5c − 6c, 5h − 6h**, and **5i − 6i** (where the acids are more potent than the corresponding esters only for the unwinding inhibition).

Regarding the substituents in *para* position of the benzyl ring, it is possible to observe that the introduction of electron-withdrawing groups gave comparable potencies than the electron-donating ones whether for the inhibition of the unwinding, ATPase, or both activities. It is worthy of note that the introduction of a chlorine atom in five-position of the indole ring confers selective inhibitory activity against unwinding for the acid derivative **5i**. Indeed, the chlorine-substituted derivative **5i** reported a 2 order of magnitude selectivity over ATPase (IC_50_ unwinding = 0.47 μM, IC_50_ ATPase = 26.35 μM), while its unsubstituted counterpart **5d** showed comparable inhibitory values against the two enzymatic activities. The replacement of the CH_2_ of the benzyl ring in position 1 of the indole core with a SO_2_ group gave comparable potencies for both the acid and ester derivatives, as it is possible to notice by comparing the SO_2_-substituted acid derivative **5f** with its CH_2_-substituted counterpart **4** (**5f**: IC_50_ unwinding = 7.54 μM, IC_50_ ATPase = 5.36 μM; **4**: IC_50_ unwinding = 12.69 μM, IC_50_ ATPase = 12.8 μM; **6f**: IC_50_ unwinding = 11.025 μM, IC_50_ ATPase = 7.075 μM; **4-COOEt**: IC_50_ unwinding = 5.49 μM, IC_50_ ATPase = 13.6 μM, data not shown^33^. With reference to the hit compound **4**, we can state that all the acids of series **5** reported higher potencies (except only 2 compounds) than that of **4** with respect to unwinding or ATPase, similarly to what observed for the ester derivatives of series **6** that were more potent or equipotent to **4-COOEt**. The best acting compound on the unwinding **5i** resulted in a 27-fold gain in activity with respect to the hit **4** and the best acting ATPase inhibitor **5c** reported a 7-fold higher inhibition in respect to **4**.

In general, the *N*-benzyl diketohexenoic derivatives proved to be more promising than the *N*-benzyl diketobutanoic ones (series **7** and **8**). Indeed, regarding the unwinding, we can observe that 3 compounds were inactive up to the maximum tested concentration (or active at 30 μM), while all the other derivatives of this series showed IC_50_ values between 1.73 μM (**8d**) and 26.02 μM (**8j**). More in detail, 1 derivative (**8d**) out of 12 tested showed high inhibitory activities, with IC_50_ value lower than 5 μM, 1 compound (**8c**) proved to be active with 5 μM < IC_50_ < 10, and 7 compounds (**7b,c,e** and **8a,b,e,j**) reported inhibitory activity with IC_50_ values > 10 μM. As concerns ATPase, 2 compounds reported no activity up to 30 μM, while the others were active up to the tested concentrations, with IC_50_s in the range 2.29 (**8e**) **− 20.34** μM (**7e**). In particular, 1 derivative (**8e**) out of 12 tested showed high inhibitory activities, with IC_50_ values lower than 5 μM, 6 compounds (**7b,j** and **8a − d**) proved to be active with 5 μM < IC_50_ < 10, and 3 compounds (**7c,e** and **8j**) reported inhibitory activity with IC_50_ values > 10 μM. Notably, the most active compound against unwinding was derivative **8d** showing IC_50_ of 1.73 μM, proving to be also the best dual inhibitor (IC_50_ ATPase = 8.7 μM), while the best acting compound *vs* ATPase was **8e** (IC_50_ of 2.29 μM). Therefore, it is possible to state that also the *N*-benzyl diketobutanoic derivatives are generally more active against ATPase than unwinding, even though the best IC_50_ value was observed for the unwinding inhibition (**8d**, IC_50_ = 1.73 μM). Interestingly, the trend according to which the esters are more active than the corresponding acids was confirmed also for the *N*-benzyl diketobutanoic, in a similar fashion to that observed for the *N*-benzyl diketohexenoic compounds.

The introduction of electron-withdrawing groups in *para* position of the benzyl ring led to comparable activity values than that of the electron-donating ones whether for the inhibition of the unwinding or of the ATPase activity. The sole exception to this trend was observed for the dual inhibition of both nsp13-associated activities. Indeed, the presence of electron-withdrawing groups led to better inhibitory potencies, as can be seen with compounds **8d,e** characterised by electron-withdrawing substituents and endowed with the best dual inhibitory profile. It is also worthy of note that the presence of an unsubstituted benzyl ring confers three-folds higher inhibitory activity against ATPase in respect to unwinding activity for the acid derivative **7j** (IC_50_ unwinding = 30 μM, IC_50_ ATPase = 10.3 μM).

In general, *N*-alkyl diketohexenoic derivatives (series **9** and **10**) showed less promising results, with 10 compounds active against the unwinding and 8 active *vs* ATPase out of the 12 tested. Among them, there was only 1 compound active in the < 5 μM range *vs* unwinding (**9c**: IC_50_ of 1.56 μM, that retained also full selectivity over ATPase) and 1 against ATPase (**10c**: IC_50_ of 4.93 μM) while most of them reported inhibitory values higher than 10 μM. A similar trend of SAR considerations observed with *N*-benzyl diketohexenoic indoles, with esters more active than the corresponding acid counterparts and with better inhibitory activities against ATPase in respect to the unwinding, was observed as well. Within these *N*-alkyl diketohexenoic indoles, saturated derivatives proved to be slightly more promising than the unsaturated ones. Indeed, among the saturated subseries there were 5 compounds active against unwinding and 5 against ATPase, while among the unsaturated subseries, 5 were active *vs* unwinding and 3 *vs* ATPase (out of 6 tested). Within the unsaturated subseries, the ester derivatives are inactive *vs* ATPase while 2 compounds showed micromolar activity *vs* unwinding. On the other hand, all the acid counterparts were active against both unwinding and ATPase, with the vinyl group proving to be more promising for a dual activity in respect to the allyl one.

Within the saturated subseries, it is possible to notice that compound **10f**, being the saturated ­counterpart of the hit **3**, showed a four-times gain in potency than the reference compound against ATPase and only a slight decrease in potency against unwinding (**10f**: IC_50_ unwinding = 13.5 μM, IC_50_ ATPase = 6.27 μM; **3**: IC_50_ unwinding = 9.51 μM, IC_50_ ATPase = 26.8 μM).

**Figure 3.**
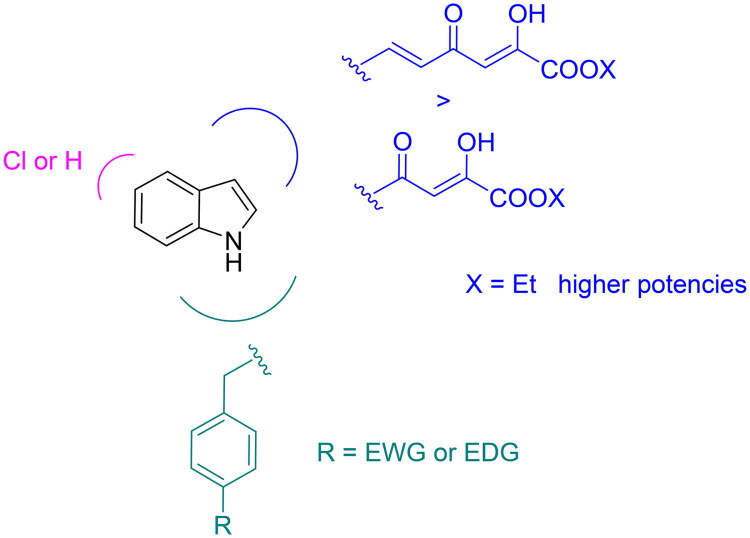
Chemical features of indolyl DKAs modulating their anti-nsp13 properties.

Altogether, combining our previous results with the new insights gained from the present study, we were then able to describe the structural features that a compound should hold to achieve nsp13 inhibition with respect to both its associated enzymatic activities (Figure 3).

In this regard, the indolyl scaffold is a good starting point since it can be easily functionalised with different chemical groups. On this scaffold, the introduction at three-position of a four- or six-carbons DKA/diketoester branch has led to many derivatives endowed with promising inhibitory profile on the enzymatic target. In particular, the presence of the diketoester side chain generally improves the inhibition of the unwinding, ATPase, or both activities. Specifically, the presence of a diketohexenoic branch is amenable for higher potencies. It is also worthy of note that a chlorine atom can be introduced in five-position of the indole scaffold, leading to selective unwinding inhibition when the acid function is also present. In addition, the introduction of variously substituted benzyl rings in position 1 of the indole core confers better inhibitory profiles against unwinding, ATPase, or both. It can be also noticed that the benzyl ring can be substituted with groups endowed with electron-withdrawing or electron-donating properties.

#### Antiviral activity

The best acting compounds against both the enzymatic activities (**5c,g**) and the best inhibitors against the single activity (**5i, 9c**) were chosen to investigate their ability to block SARS-CoV-2 replication in infected cells. Moreover, compound **8d** was also added being the most potent compound active against both the enzymatic activities among the diketobutanoic derivatives. Lastly, the ester-acid couple **5e** and **6e** was also tested given that, in this case, we observed no marked difference between the inhibitory activity of the ester and that of the acid.

**Figure 4.**
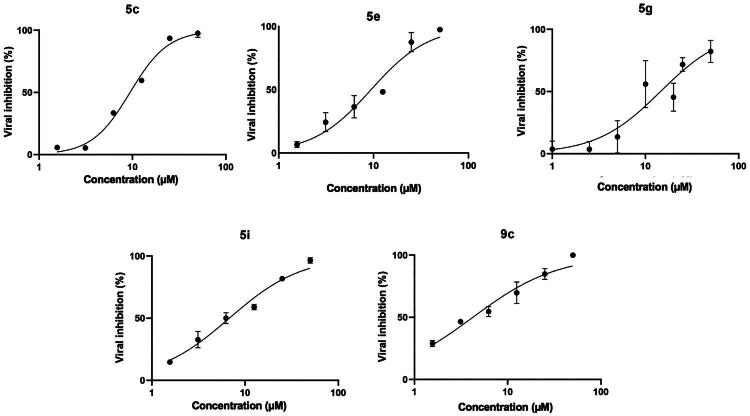
Antiviral effect of DKAs inhibitors against SARS-CoV-2. SARS-CoV-2 infected cells were treated with the compounds at concentrations ranging from 1 to 50 μM, and viral titre inhibition was calculated by plaque assay. Values are expressed as means ± the *SD* from three experiments, each performed in triplicate.

In the first set of experiments, the effect of compounds on Vero cell viability was evaluated. To this end, confluent monolayers of cells were treated with different concentrations (100–10 μM) of exemplificative indolyl DKA derivatives for 24, 48, and 72 h. Microscopic examination showed no significant CPE in cells treated with the compounds at a concentration less than 50 μM while marked CPE were observed at concentration of 100 μM. Based on these results, the antiviral activity of the DKA derivatives against SARS-CoV-2 was tested through the plaque assay by evaluating the compounds at concentrations ranging from 1 to 50 μM (Figure 4).

Untreated-infected cells were used as a positive control of viral infection (0.01 MOI). The results indicate that compounds **5c,e,g,i** and **9c** inhibit SARS-CoV-2 with varying degrees of effectiveness (Table 2). On the other hand, compound **6e** showed low activity while derivative **8d** not showed any antiviral activity up to the maximum concentration tested.

**Table 2.** Antiviral activity against SARS-CoV-2 activities of derivatives **5c,e,g,i, 6e, 8d,** and **9c**.

Cpd	EC_50_ (µM)^a^	CC_50_^b^
**5c**	9.41 ± 0.91	>100
**5e**	9.397 ± 2.26	>100
**5 g**	14.48 ± 4.20	>100
**5i**	6.769 ± 1.17	>100
**6e**	50	>100
**8d**	> 50	>100
**9c**	4.231 ± 0.96	>100
**Camptothecin**	–	11.09 ± 0.16
**4** ^c^	1.03 ± 0.24	>264

^a^Compound concentration required to reduce the SARS-CoV-2 replication in Vero cells by 50% (*μ*M).

^b^Compound concentration required to reduce Vero cells viability by 50% (*μ*M).

^c^From reference^33^.

To assess the *in vitro* inhibition potency of the selected compounds, the 50% inhibitory concentration (EC_50_) for each compound was calculated from the experimental curves to evaluate the *in vitro* inhibitory activity of the compounds. The values reported in Table 2 showed that compound **9c** was the best acting inhibitor (EC_50_ = 4.231 μM) and that compounds **5c,e,g,h** block viral replication in the low micromolar range. In detail, compound **5i** inhibits SARS-CoV-2 replication slightly more efficiently than **5e, 5c**, and **5g** (EC_50_ values of 6.769, 9.397, 9.41, and 14.48 μM, respectively). The 95% confidence interval (CI) for each compound was: **5i**: 5.687 − 8.032; **5e**: 7.364 **−** 11.90; **5c**: 8.540 − 10.36; **5g**: 10.90 − 19.31; **9c**: 3.332 − 5.257.

#### Evaluation of inhibition type

**Figure 5.**
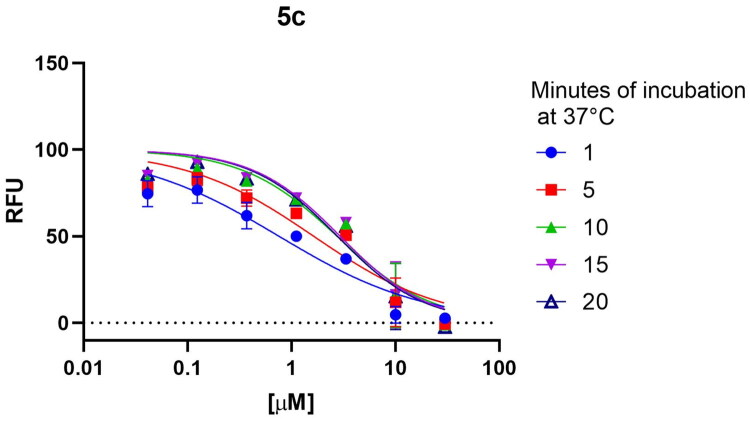
Incubation-dependent potency of **5c**.

To investigate the type of inhibition of this set of compounds as reversible/irreversible and covalent/non-covalent inhibitors, we performed additional assays on the most promising compound **5c**. Considering that the incubation-dependent potency should lead to a decrease in IC_50_ values in case of covalent-irreversible inhibition^43^, the compound activity was monitored at different time-points during the incubation time with the inhibitor, checking the amount of reaction products: 1 min, 5 min, 10 min, 15 min, 20 min after the activation of the reaction with the substrate (Figure 5). The time-increased obtained IC_50_ values (0.76 µM; 1.64 µM; 2.75 µM; 2.94 µM and 2.76 µM, respectively) indicate that the nature of the inhibition is non-covalent/reversible. To further confirm the hypothesis, we extended the pre-incubation time of compound and enzyme, before the activation of the reaction with the substrate, to +20 min and +50 min at RT and we observed that this pre-incubation did not lead to an increase of the inhibitory potency.

### Molecular docking prediction

A molecular docking protocol was employed to predict the potential binding mode of the DKA compounds on the nsp13 complex. Following the indication of the binding within the RecA2 domain as the most supported by our previously reported experimental findings^33^, we hypothesised the most important residues for both unwinding and ATPase inhibition performing docking procedures on **5c**, **5g**, **5i, 8d** and **8e**.

**Figure 6.**
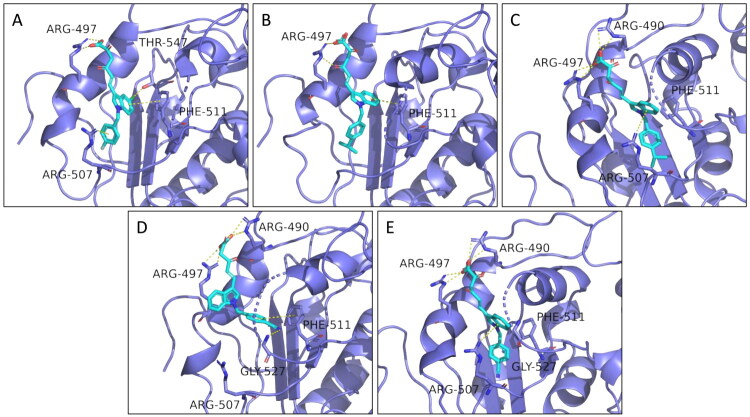
Predicted binding modes for **5i** (A), **5c** (B,C), and **5g** (D,E).

In general, the newly designed indolyl derivatives of series **5 **−** 8** demonstrated activity against both enzymatic functions, showing measurable IC_50_ values below 30 μM concentration. Among them, the *N*-benzyl diketohexenoic derivatives (series **5** and **6**) revealed highly promising inhibitory potencies against both unwinding and ATPase activities. In particular, the most active compound against unwinding was derivative **5i** (IC_50_ of 0.47 μM), while the best-performing compounds against ATPase were **5c** and **5g**, with IC_50_ values of 1.8 and 1.91 μM, respectively, also proving to be the best dual inhibitors. To elucidate the inhibitory properties of these newly synthesised indolyl DKA derivatives at the atomic level, molecular modelling studies were attended. The 3D model of the SARS-CoV-2 nsp13 helicase protein used in this study is the previously published by our group^33^. After generating the model, docking calculations were attained for **5c**, **5g** and **5i**, resulting in the binding pose depicted in Figure 6.

Compounds **5i** (Figure 6(A)) and **5c** (Figure 6(B)) exhibit a similar binding mode, with the indole ring establishing a π-π stacking with Phe511. This residue is also involved in a T-shaped π-π stacking with the *p*-CN-benzyl moiety of **5g** (Figure 6(D)). Furthermore, this type of binding is stabilised by the presence of the CN group that is capable to interact *via* H-bond with the backbone of Gly527.

Among these compounds, **5i** (Figure 6(A)) also shows an interesting additional interaction. Indeed, the chlorine-substituted derivative is the only one capable of establishing a halogen bond with Thr547. The introduction of a chlorine atom in five-position of the indole ring would seem to be the optimal substitution for establishing this type of interaction.

Regarding **5c** and **5g**, these two compounds show an additional binding mode, as shown in Figure 6(C,E), respectively. Indeed, both of them would seem to have a possible cation-π interaction between the indole ring and Arg507. In favour of this new type of interaction, **5c** and **5g** align themselves in a way that π-π stacking with Phe511 is lost.

All the compounds interact *via* DKA portion with basic residues Arg497 (Figure 6(A–E)) and Arg490 (Figure 6(C,E)) through salt bridges and H-bond interactions. This finding is consistent with the *in vitro* biological assays, in which the ester counterparts **6c**, **6g** and **6i** are less active than the acid ones **5c**, **5g** and **5i**, respectively.

It bears mentioning that the poses obtained for **5g** (Figure 6(D,E)) are very similar to those obtained for **5e** (data not shown). Indeed, these two derivatives are both *N*-benzyl diketohexenoic acids and only differ in one substituent (Table 1). However, the *p*-CN-benzyl moiety of **5g** has characteristics such as polarity, electron-withdrawing and H-bond accepting capacity comparable to those of *p*-OCF_3_-benzyl moiety of **5e**. Considering this, it is not surprising to observe comparable *in cellulo* activity of these two compounds (Table 2).

**Figure 7.**
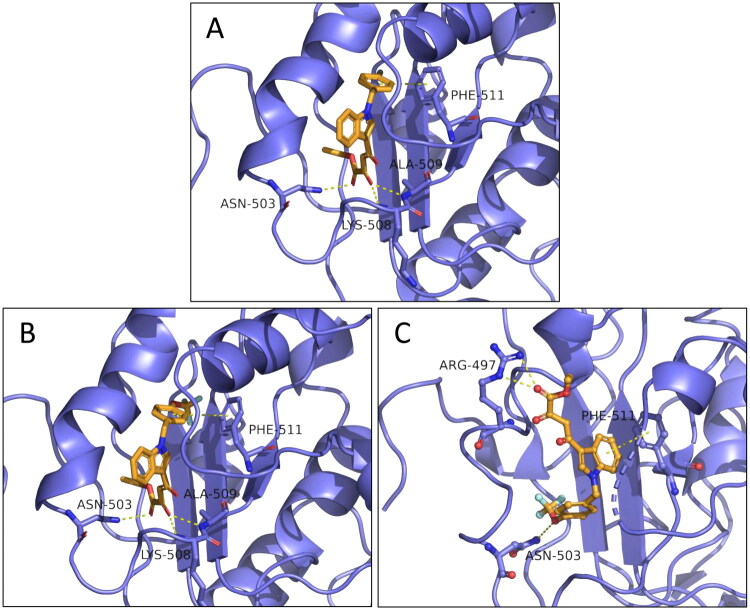
Predicted binding modes for **8d** (A) and **8e** (B, C).

Despite the *N*-benzyl diketobutanoic derivatives (series **7** and **8**) proved to be less promising than the *N*-benzyl diketohexenoic ones (series **5** and **6**), some molecules exhibit interesting inhibition profiles. Indeed, among them, **8d** shows high inhibitory activity against unwinding (IC_50_ of 1.73 μM), proving to have also the best dual activity (IC_50_ ATPase = 8.7 μM). Regarding the ATPase activity, the most promising compound was derivative **8e** (IC_50_ of 2.29 μM). To predict their binding poses, the same SARS-CoV-2 nsp13 helicase model was used to perform docking calculations on **8d** and **8e**, the results of which are shown in Figure 7.

The *N*-benzyl diketobutanoic derivatives shows interesting binding poses. Indeed, the shorter DKA chain compared to diketohexenoic ones allows the molecules to fit in the same allosteric pocket but in a different way (Figure 7(A–C)). It is possible to appreciate how **8d** (Figure 7(A)) and **8e** (Figure 7(B)) are able to anchor themselves to RecA2 domain interacting with their diketo esters portion *via* H-bond interactions with both the backbone and the chains of residues Asn503, Lys508 and Ala509. Moreover, the benzyl moiety of these two derivatives establishes a π-π stacking with Phe511. It is worthy of note that the same residue is involved into a π-π stacking interactions with the indole portion **5i** (Figure 6(A)) and **5c** (Figure 6(B)) and with *p*-cyano-benzyl moiety of **5g** (Figure 6(D)).

About **8e**, this compound shows an additional binding mode (Figure 7(D)). Indeed, the presence of OCF_3_ moiety allows the molecule to establish a H-bond interaction with Asn503. These two binding modes, which are equivalent in terms of probability, are both possible. We hypothesise that the pronounced inhibitory activity towards the ATPase of **8e** is due to this duality. The unwinding activity is less pronounced than for the other derivative **8d** in which a single binding mode is possible, due to the absence of oxygen atoms at the *para*-position of the benzyl moiety capable of acting as H-bond acceptors. Actually, even if many small-molecules crystallographic structures contain X-H…F-C interactions (X = N or O), it has been demonstrated that these type of contacts are so weak that they cannot be considered to be H-bonds, since they rather have similar energies to those of van der Waals complexes^44^^,^^45^.

In light of these results, we speculate that: (1) Phe511 is the most important residue for unwinding inhibition, which can be increased involving adjacent residues such as Thr547 (Figure 6(A)). Additionally, the introduction of electron-withdrawing groups on the aryl moiety responsible for π-π stacking with Phe511 can improve the inhibition (Figure 6(A,B,D) and Figure 7(A,B)); (2) Interactions with Arg507, Lys508 and nearby residues such as Asn503 and Ala509 are essential for ATPase inhibition (Figure 6(C,E) and Figure 7(A–C)).

MD simulations on **5g** were then performed to probe the stability of these interactions. The structure was subjected to a 150 ns long MD simulation and the results analysed examining ligand and protein root-mean-square deviation and fluctuation (RMSD and RMSF, respectively) and the stability of the interactions between ligand and adjacent residues. **5g** binding mode demonstrated to be very stable throughout the entire prediction run, showing an average RMSD value of 1,16 Å (Figure S1). The protein RMSD, on the other hand, revealed peaks up to over 7 Å in the last nanoseconds of the simulation (Figure S1). By analysing the RMSF of protein residues, it is evident that the most involved domains in the **5g**-induced conformational change are ZBD (residues 1 to 100), SD (101 to 150) and 1B domain (150 to 261), with fluctuations up to 6.93, 5.61 and 6.82 Å, respectively, as shown in Figure S2. Interestingly, these results are very similar to those already published by Perez-Lemus *et al.*^46^, who discussed how SSYA10-001, bananin, and chromone inhibitors induce strain in the same regions.

As expected, most ligand-protein interactions occur at the DKA chain, which interacts with the positively charged residues Arg490 and Arg497 (Figure S3). The latter also shows a weak cation-π interaction with the indole ring which, surprisingly, completely loses interactions with Arg507. However, Phe511 proves to be important for **5g** binding, interacting with the *p*-cyano-benzyl moiety *via* π-π stacking. As discussed above, this type of interaction is favoured by the presence of electron-withdrawing groups.

## Conclusions

The recent namely coronavirus disease 2019 (COVID-19) pandemic, caused by SARS-CoV-2, is a glaring example of how the emergence of a new infectious agent in our globalised society can have devastating effects. In the light of this, the development of antiviral agents acting against emerging and re-emerging pathogenic viruses is mandatory. In this context, the SARS-CoV-2 nsp13 enzyme plays a key role thanks to its pivotal role in the viral life cycle and a high interspecific similarity among coronaviruses. Herein, we reported the synthesis of a new series of indolyl DKA derivatives structurally related to the ones we recently described, in order to deepen the SAR studies within this class of compounds. Owing to their better dual inhibitory profile against nsp13-associated unwinding and ATPase activities, the *N*-benzyl diketohexenoic derivatives (series **5** and **6**) were found to be the most promising among the newly synthesised compounds. Interestingly, *in vitro* assays showed their ability to block viral replication in infected cells in the low micromolar range, in addition to no cytotoxicity. Furthermore, docking studies within the allosteric pocket located in the Rec2A domain, allowed us to rationalise the structural features involved in the interaction with the enzyme, providing valuable insights to guide medicinal chemistry research for the development of new SARS-CoV-2 nsp13 inhibitors.

## Supplementary Material

Supplemental_NSP13_Inhibitors_Albano_et_al_CLEAN_COPY (1).docx

## Data Availability

The data presented in this study are available on request from the corresponding author.
