# Supplementary material for: N-alkyl and N-benzyl indoles are anti-SARS-CoV-2 agents and nsp13 inhibitors
Source: J Enzyme Inhib Med Chem. 2025 Aug 12;40(1):2539445. doi: 10.1080/14756366.2025.2539445 (PMC12344683; doi:10.1080/14756366.2025.2539445)
Supplement: Supplemental_NSP13_Inhibitors_Albano_et_al_CLEAN_COPY (1).docx [file IENZ_A_2539445_SM1901.docx]

Supplemental materials for

***N*-Alkyl and *N*-Benzyl Indoles are Anti-SARS-CoV-2 Agents and Nsp13 Inhibitors**

**Aurora Albano^1^, Roberta Emmolo^2^, Riccardo De Santis^3,4^, Elisa Patacchini^1^, Valentina Noemi Madia^1,*^, Stefania Maloccu^2^, Davide Ialongo^1^, Giuseppe Ruggieri^5,1^, Merve Arpacioglu^1^, Luigi Scipione^1^, Francesco Saccoliti^6,1^, Donatella Amatore^3^, Giorgia Grilli^3^, Florigio Lista^3^, Francesca Esposito^2^, Enzo Tramontano^2^, Angela Corona^2^, Roberto Di Santo^1^ and Roberta Costi^1^**

^1^ Istituto Pasteur-Fondazione Cenci Bolognetti, Dipartimento di Chimica e Tecnologie del Farmaco, “Sapienza” Università di Roma, p.le Aldo Moro 5, I-00185 Rome, Italy; aurora.albano@uniroma1.it (A.A.); elisa.patacchini@uniroma1.it (E.P.); valentinanoemi.madia@uniroma1.it (V.N.M.); da.ial@outlook.com (D.I.); merve.arpacioglu@uniroma1.it (M.A.); luigi.scipione@uniroma1.it (L.S.); roberto.disanto@uniroma1.it (R. Di S.); roberta.costi@uniroma1.it (R.C.).

^2^ Dipartimento di Scienze della Vita e dell’Ambiente Sezione biomedica, Laboratorio di Virologia Molecolare Blocco E, primo piano Università di Cagliari Cittadella Universitaria di Monserrato, SS554 -09042 Monserrato (CA) Italia; roberta.emmolo@unica.it (R.E.); stefania.maloccu@unica.it (S.M.); francescaesposito@unica.it (F.E.); tramon@unica.it (E.T.); angela.corona@unica.it (A.C.).

^3^ Istituto di Scienze Biomediche della Difesa, 00184 Roma, Italy; donatella.amatore@persociv.difesa.it (D.A.); giorgia.grilli@persociv.difesa.it (G.G.); florigio.lista@esercito.difesa.it (F.L.).

^4^ Dipartimento di Sanità Pubblica e Malattie Infettive, Sapienza, Università di Roma, 00161 Roma, Italy; r.desantis@uniroma1.it (R. De S.).

^5^ Dottorato di Interesse Nazionale in One Health approaches to infectious diseases and life science research, Dipartimento di Sanità Pubblica, Medicina Sperimentale e Forense, Università degli Studi di Pavia, Pavia, 27100, Italia; giuseppe.ruggieri01@univeristadipavia.it (G.R.).

^6^ Department of Life Science, Health, and Health Professions, Link Campus University, Via del Casale di San Pio V 44, I-00165, Rome, Italy; f.saccoliti@unilink.it (F.S.).

***** Correspondence: valentinanoemi.madia@uniroma1.it; Tel.: +39 0649913965.

**Contents:**

| 1. MD simulations results | 2-4 |
| --- | --- |
| 1. Specific Procedures and Characterization | 5-14 |
| 1. References | 15 |
| 1. Figures S4 – S75: FTIR, ^1^H NMR, ^13^C NMR, MS (ESI) Spectra for compounds **5b,c,e,f, 6b,c,e,f, 7a−c,e, 8a−c,e, 9d** and **10d** | 16-88 |

**MD simulations results**


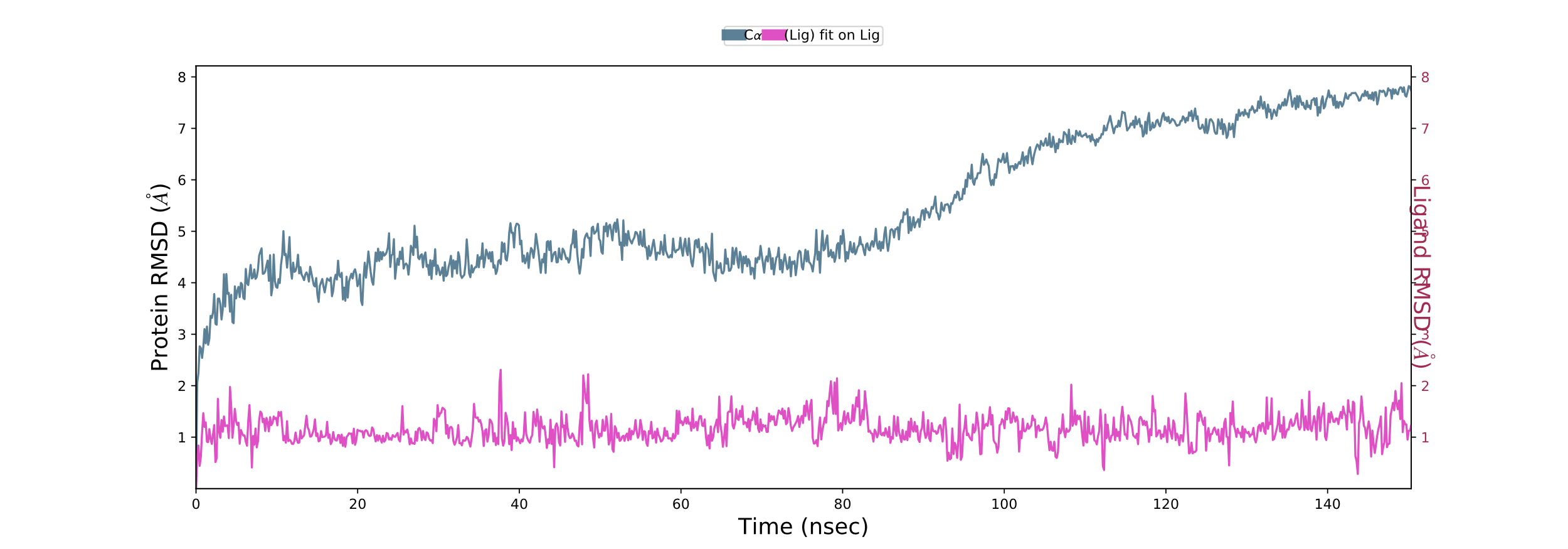
Figure S1: RMSD calculated all long MD simulation time (150 ns) for **5g**-nsp13 complex. Ligand RMSD is shown in pink, protein RMSD in blue.


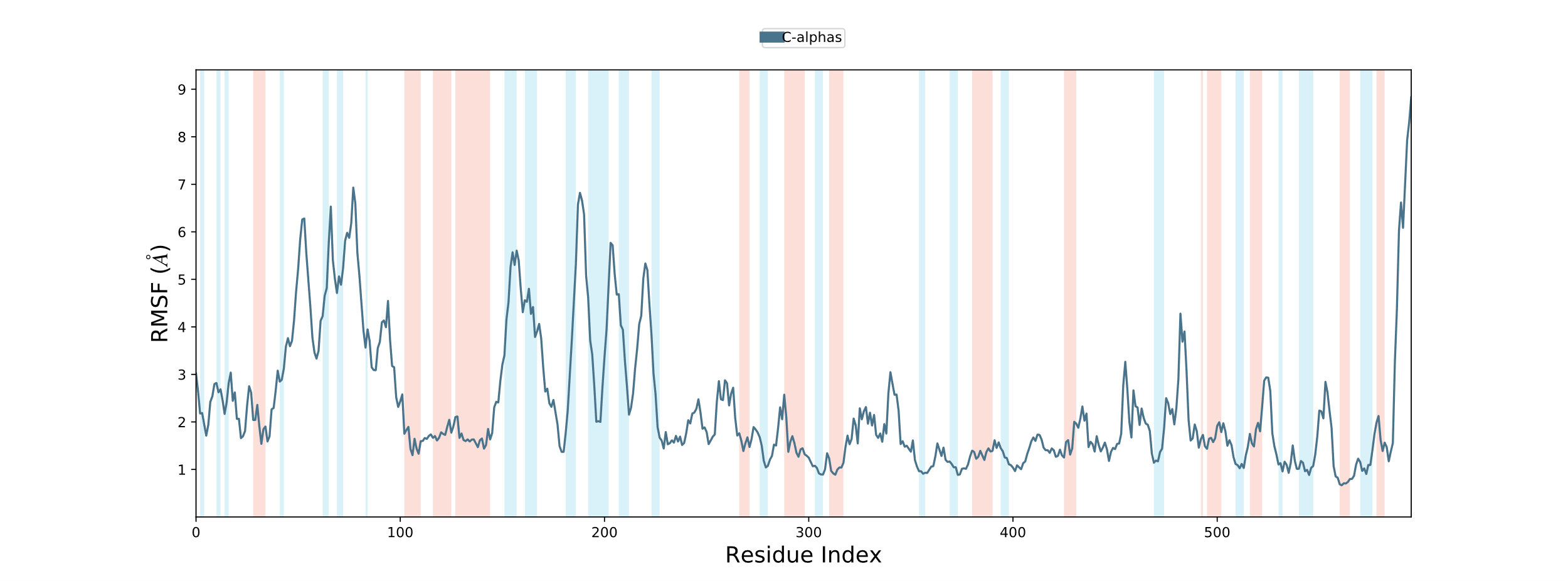


Figure S2: RMSF plot. Peaks indicate areas of the protein that fluctuate the most during the simulation. Alpha-helical and beta-strand regions that persist over 70% of the entire simulation are highlighted in orange and blue backgrounds, respectively.


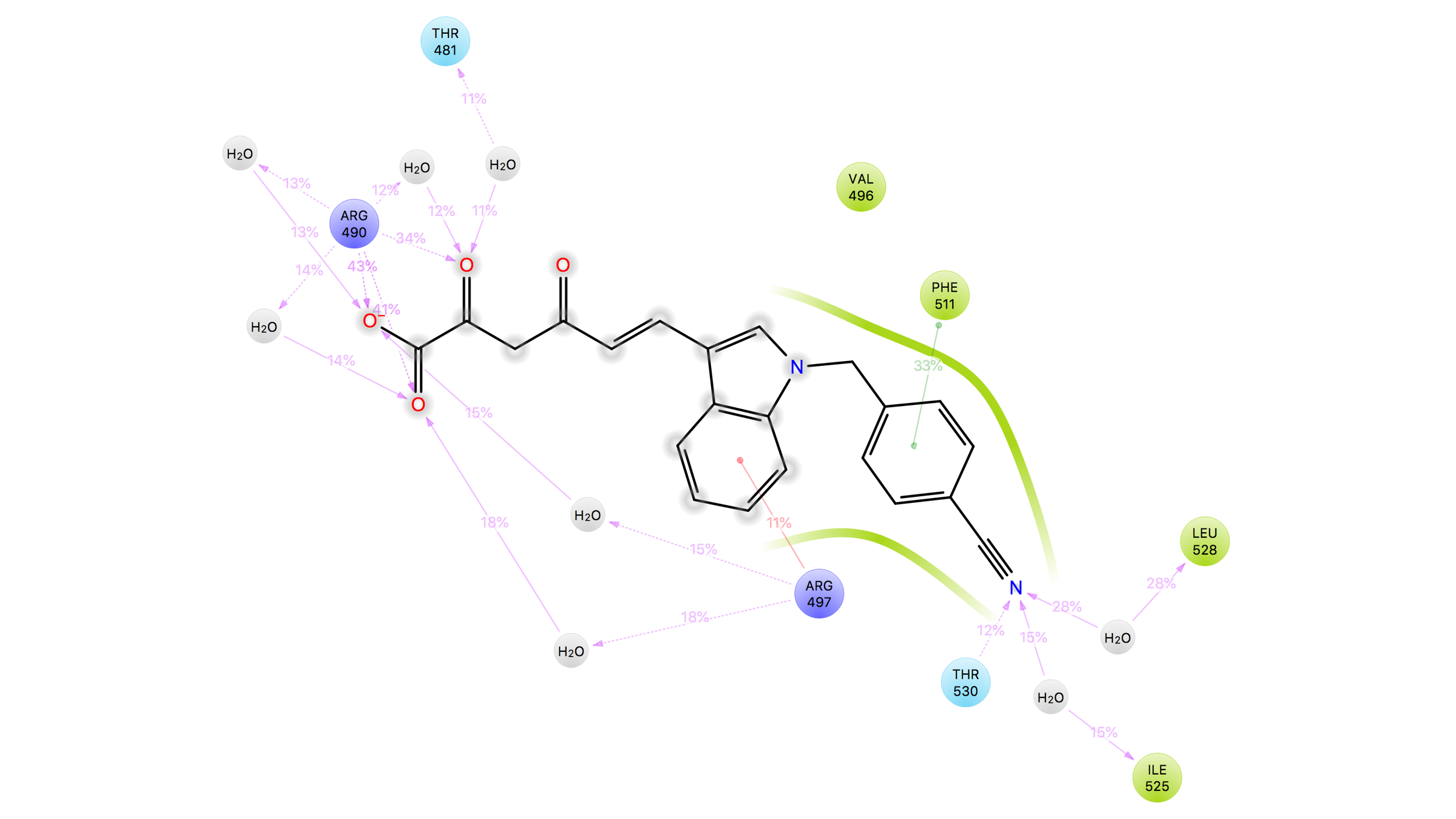


Figure S3: Schematic overview of ligand atoms interactions with protein residues.

**Specific Procedures and Characterization**

*(2Z,5E)-2-hydroxy-6-(1-(4-methoxybenzyl)-1H-indol-3-yl)-4-oxohexa-2,5-dienoic acid (****5a****)*. Synthesis, analytical, and spectroscopic data are reported in the literature [1].

*(2Z,5E)-2-hydroxy-6-(1-(4-methylbenzyl)-1H-indol-3-yl)-4-oxohexa-2,5-dienoic acid (****5b****)*. Compound **5b** was prepared from **6b** by means of GP-D; 20 min; methanol; 91% as a red solid; decomposes at 88 °C; IR ν OH 3298, CO acid 1754, CO ketone 1592 cm^-1^; ^1^H NMR (400 MHz, DMSO-*d*_6_) δ 13.79 (bs, 1H, OH), 8.23 (s, 1H, indole C2-H), 8.12 – 8.10 (m, 1H, indole C4-H), 8.03 (d, *J* = 15.8 Hz, 1H, hexenoate C6-H), 7.58 (d, *J* = 7.8 Hz, 1H, indole C7-H), 7.26 – 7.14 (m, 6H, benzene H and indole C5-H and C6-H), 6.94 (d, *J* = 15.8 Hz, 1H, hexenoate C5-H), 6.67 (s, 1H, hexenoate C3-H), 5.44 (s, 2H, CH_2_), 2.24 (s, 3H, CH_3_). ^13^C NMR (101 MHz, CD_3_OD) δ 197.12, 179.65, 171.55, 147.80, 146.96, 146.76, 146.48, 143.37, 138.72, 136.83, 135.06, 132.69, 131.31, 130.45, 126.73, 122.05, 121.01, 110.61, 71.31, 30.14. Anal. calcd for C_22_H_19_NO_4_: C, 73.12; H, 5.30; N, 3.88%. Found: C, 73.05; H, 5.29; N, 3.87%. MS (ESI) *m/z* calc. [M+H]^+^ for C_22_H_20_NO_4_ ^+^: 362.40; found: 384.39 [M+Na]^+^.

*(2Z,5E)-2-hydroxy-6-(1-(4-isopropylbenzyl)-1H-indol-3-yl)-4-oxohexa-2,5-dienoic acid (****5c****)*. Compound **5c** was prepared from **6c** by means of GP-D; 15 min; methanol; 80% as a red solid; 145 - 147 °C; IR ν OH 2965, CO acid 1708, CO ketone 1591 cm^-1^; ^1^H NMR (400 MHz, DMSO-*d*_6_) δ 13.78 (bs, 1H, OH), 8.24 (s, 1H, indole C2-H), 8.11 (d, *J* = 7.2 Hz, 1H, indole C4-H), 8.04 (d, *J* = 15.8 Hz, 1H, hexenoate C6-H), 7.61 (d, *J* = 7.1 Hz, 1H, indole C7-H), 7.25 – 7.20 (m, 6H, benzene H and indole C5-H and C6-H), 6.92 (d, *J* = 15.9 Hz, 1H, hexenoate C5-H), 6.68 (s, 1H, hexenoate C3-H), 5.44 (s, 2H, CH_2_), 2.84 – 2.81 (m, 1H, CH), 1.14 (d, *J* = 6.9 Hz, 6H, CH_3_). ^13^C NMR (101 MHz, DMSO-*d*_6_) δ 163.69, 147.93, 137.47, 136.88, 134.43, 127.35, 126.60, 125.58, 123.18, 121.75, 120.90, 117.64, 112.54, 111.47, 101.05, 49.33, 33.09, 23.80. Anal. calcd for C_24_H_23_NO_4_: C, 74.02; H, 5.95; N, 3.60%. Found: C, 73.98; H, 5.96; N, 3.61%. MS (ESI) *m/z* calc. [M+H]^+^ for C_24_H_24_NO_4_ ^+^: 390.45; found: 390.44 and 412.44 [M+Na]^+^.

*(2Z,5E)-6-(1-(4-fluorobenzyl)-1H-indol-3-yl)-2-hydroxy-4-oxohexa-2,5-dienoic acid (****5d****)*. Synthesis, analytical, and spectroscopic data are reported in the literature [1].

*(2Z,5E)-2-hydroxy-4-oxo-6-(1-(4-(trifluoromethoxy)benzyl)-1H-indol-3-yl)hexa-2,5-dienoic acid (****5e****)*. Compound **5e** was prepared from **6e** by means of GP-D; 30 min; DMF/H_2_O; 64% as a red solid; 157 - 159 °C; IR ν OH 3068, CO acid 1727, CO ketone 1594 cm^-1^; ^1^H NMR (400 MHz, DMSO-*d*_6_) δ 13.83 (bs, 1H, OH), 8.25 (s, 1H, indole C2-H), 8.13 (d, *J* = 7.4 Hz, 1H, indole C4-H), 8.03 (d, *J* = 15.8 Hz, 1H, hexenoate C6-H), 7.60 (d, *J* = 7.3 Hz, 1H, indole C7-H), 7.40 – 7.18 (m, 6H, benzene H and indole C5-H and C6-H), 6.94 (d, *J* = 15.8 Hz, 1H, hexenoate C5-H), 6.69 (s, 1H, hexenoate C3-H), 5.55 (s, 2H, CH_2_). ^13^C NMR (101 MHz, DMSO-*d*_6_) δ 187.85, 163.63, 161.04, 147.76, 137.81, 137.42, 136.85, 136.58, 129.16, 125.60, 123.33, 121.89, 121.37, 121.30, 121.00, 118.75, 117.91, 112.76, 111.37, 101.07, 48.72. Anal. calcd for C_22_H_16_F_3_NO_5_: C, 61.26; H, 3.74; N, 3.25%. Found: C, 61.13; H, 3.75; N, 3.24%. MS (ESI) *m/z* calc. [M+H]^+^ for C_22_H_17_F_3_NO_5_ ^+^: 432.37; found: 432.40 and 435.38 [M+Na]^+^.

*(2Z,5E)-2-hydroxy-4-oxo-6-(1-(phenylsulfonyl)-1H-indol-3-yl)hexa-2,5-dienoic acid (****5f****)*. Compound **5f** was prepared from **6f** by means of GP-D; 30 min; washed with ethanol; 75% as a brown solid; decomposes at 157 °C; IR ν OH 3123, CO acid 1709, CO ketone 1529, SO_2_ 1364 and 1133 cm^-1^; ^1^H NMR (400 MHz, DMSO-*d*_6_) δ 13.90 (bs, 1H, OH), 8.56 (s, 1H, indole C2-H), 8.18 (d, *J* = 7.9 Hz, 1H, indole C4-H), 8.07 – 7.92 (m, 4H, benzene H, indole C7-H and hexenoate C6-H), 7.73 (t, *J* = 7.3 Hz, 1H, benzene H), 7.63 (t, *J* = 7.7 Hz, 2H, benzene H), 7.46 – 7.38 (m, 2H, indole C5-H and C6-H), 7.19 (d, *J* = 16.4 Hz, 1H, hexenoate C5-H), 6.74 (s, 1H, hexenoate C3-H). ^13^C NMR (101 MHz, DMSO-*d*_6_) δ 197.91, 185.69, 173.97, 163.25, 136.58, 136.50, 135.14, 134.94, 131.56, 130.10, 127.35, 126.94, 125.88, 124.55, 123.54, 121.61, 118.49, 113.43, 101.32. Anal. calcd for C_20_H_15_NO_6_S: C, 60.45; H, 3.80; N, 3.52; S, 8.07%. Found: C, 60.35; H, 3.81; N, 3.51; S, 8.05%. MS (ESI) *m/z* calc. [M+H]^+^ for C_20_H_16_NO_6_S ^+^: 398.40; found: 398.33 and 420.30 [M+Na]^+^.

*(2Z,5E)-6-(1-(4-cyanobenzyl)-1H-indol-3-yl)-2-hydroxy-4-oxohexa-2,5-dienoic acid (****5g****)*. Synthesis, analytical, and spectroscopic data are reported in the literature [1].

*(2Z,5E)-2-hydroxy-6-(1-(4-hydroxybenzyl)-1H-indol-3-yl)-4-oxohexa-2,5-dienoic acid (****5h****)*. Synthesis, analytical, and spectroscopic data are reported in the literature [1].

*(2Z,5E)-6-(5-chloro-1-(4-fluorobenzyl)-1H-indol-3-yl)-2-hydroxy-4-oxohexa-2,5-dienoic acid (****5i****)*. Synthesis, analytical, and spectroscopic data are reported in the literature [1].

*Ethyl (2Z,5E)-2-hydroxy-6-(1-(4-methoxybenzyl)-1H-indol-3-yl)-4-oxohexa-2,5-dienoate (****6a****)*. Synthesis, analytical, and spectroscopic data are reported in the literature [1].

*Ethyl (2Z,5E)-2-hydroxy-6-(1-(4-methylbenzyl)-1H-indol-3-yl)-4-oxohexa-2,5-dienoate (****6b****)*. Compound **6b** was prepared from **12b** by means of GP-C; 20 min; washed with isopropanol; 92% as a red solid; decomposes at 189 °C; IR ν CO ester 1723, CO ketone 1605 cm^-1^; ^1^H NMR (400 MHz, DMSO-*d*_6_) δ 8.24 (s, 1H, indole C2-H), 8.12 (d, *J* = 7.4 Hz, 1H, indole C4-H), 8.04 (d, *J* = 15.8 Hz, 1H, hexenoate C6-H), 7.58 (d, *J* = 7.6 Hz, 1H, indole C7-H), 7.27 – 7.12 (m, 6H, benzene H and indole C5-H and C6-H), 6.95 (d, *J* = 15.8 Hz, 1H, hexenoate C5-H), 6.69 (s, 1H, hexenoate C3-H), 5.44 (s, 2H, CH_2_), 4.29 (q, *J* = 7.2 Hz, 2H, *CH_2_*CH_3_), 2.24 (s, 3H, CH_3_), 1.30 (t, *J* = 7.1 Hz, 3H, CH_2_*CH_3_*). ^13^C NMR (101 MHz, DMSO-*d*_6_) δ 187.64, 170.17, 162.07, 138.32, 137.48, 137.28, 137.00, 133.89, 129.24, 127.35, 125.58, 123.21, 121.83, 120.97, 117.25, 112.56, 111.53, 101.13, 61.83, 49.44, 20.66, 13.94. Anal. calcd for C_24_H_23_NO_4_: C, 74.02; H, 5.95; N, 3.60%. Found: C, 73.98; H, 5.96; N, 3.59%. MS (ESI) *m/z* calc. [M+H]^+^ for C_24_H_24_NO_4_^+^: 390.45; found: 412.60 [M+Na]^+^.

*Ethyl (2Z,5E)-2-hydroxy-6-(1-(4-isopropylbenzyl)-1H-indol-3-yl)-4-oxohexa-2,5-dienoate (****6c****)*. Compound **6c** was prepared from **12c** by means of GP-C; 30 min; washed with methanol; 63% as a red solid; 97 - 100 °C; IR ν CO ester 1725, CO ketone 1610 cm^-1^; ^1^H NMR (400 MHz, CD_3_OD-*d*_6_) δ 8.08 (d, *J* = 15.7 Hz, 1H, hexenoate C6-H), 7.99 – 7.97 (m, 1H, indole C4-H), 7.89 (s, 1H, indole C2-H), 7.45 – 7.42 (m, 1H, indole C7-H), 7.27 – 7.25 (m, 2H, indole C5-H and C6-H), 7.19 (d, *J* = 8.2 Hz, 2H, benzene H), 7.13 (d, *J* = 8.2 Hz, 2H, benzene H), 6.78 (d, *J* = 15.7 Hz, 1H, hexenoate C5-H), 6.56 (s, 1H, hexenoate C3-H), 5.40 (s, 2H, CH_2_), 4.34 (q, *J* = 7.1 Hz, 2H, *CH_2_*CH_3_), 2.88 – 2.84 (m, 1H, CH), 1.38 (t, *J* = 7.1 Hz, 3H, CH_2_*CH_3_*), 1.20 (d, *J* = 6.9 Hz, 6H, CH_3_). ^13^C NMR (101 MHz, DMSO-*d*_6_) δ 187.64, 170.19, 162.07, 147.94, 138.32, 137.51, 137.27, 134.38, 127.36, 126.60, 125.55, 123.25, 121.83, 120.98, 117.28, 112.61, 111.51, 101.13, 61.83, 49.36, 33.09, 23.79, 13.94. Anal. calcd for C_26_H_27_NO_4_: C, 74.80; H, 6.52; N, 3.35%. Found: C, 74.69; H, 6.51; N, 3.34%. MS (ESI) *m/z* calc. [M+H]^+^ for C_26_H_28_NO_4_^+^: 418.51; found: 440.48 [M+Na]^+^.

*Ethyl (2Z,5E)-6-(1-(4-fluorobenzyl)-1H-indol-3-yl)-2-hydroxy-4-oxohexa-2,5-dienoate (****6d****)*. Synthesis, analytical, and spectroscopic data are reported in the literature [1].

*Ethyl (2Z,5E)-2-hydroxy-4-oxo-6-(1-(4-(trifluoromethoxy)benzyl)-1H-indol-3-yl)hexa-2,5-dienoate (****6e****)*. Compound **6e** was prepared from **12e** by means of GP-C; 45 min; washed with isopropanol; 95% as a brown solid; decomposes at 120 °C; IR ν CO ester 1729, CO ketone 1614 cm^-1^;^1^H NMR (400 MHz, DMSO-*d*_6_) δ 8.27 (s, 1H, indole C2-H), 8.15 (d, *J* = 7.5 Hz, 1H, indole C4-H), 8.04 (d, *J* = 15.8 Hz, 1H, hexenoate C6-H), 7.60 (d, *J* = 7.4 Hz, 1H, indole C7-H), 7.41 – 7.33 (m, 4H, benzene H), 7.29 – 7.25 (m, 2H, indole C5-H and C6-H), 6.97 (d, *J* = 15.8 Hz, 1H, hexenoate C5-H), 6.70 (s, 1H, hexenoate C3-H), 5.55 (s, 2H, CH_2_), 4.29 (q, *J* = 7.1 Hz, 2H, *CH_2_*CH_3_), 1.30 (t, *J* = 7.1 Hz, 3H, CH_2_*CH_3_*). ^13^C NMR (101 MHz, DMSO-*d*_6_) δ 187.57, 170.31, 162.05, 147.75, 138.16, 137.45, 137.20, 136.54, 129.18, 125.55, 123.38, 121.95, 121.36, 121.07, 117.55, 112.81, 111.40, 101.16, 61.85, 48.72, 13.94. Anal. calcd for C_24_H_20_F_3_NO_5_: C, 62.75; H, 4.39; N, 3.05%. Found: C, 62.64; H, 4.40; N, 3.04%. MS (ESI) *m/z* calc. [M+H]^+^ for C_24_H_21_F_3_NO_5_^+^: 460.42; found: 460.45 and 483.76 [M+Na]^+^.

*Ethyl (2Z,5E)-2-hydroxy-4-oxo-6-(1-(phenylsulfonyl)-1H-indol-3-yl)hexa-2,5-dienoate (****6f****)*. Compound **6f** was prepared from **12f** by means of GP-C; 2 h; washed with ethanol; 58% as a red solid; decomposes at 170 °C; IR ν OH 3130, CO ester 1720, CO ketone 1590, SO_2_ 1366 and 1119 cm^-1^;^1^H NMR (400 MHz, DMSO-*d*_6_) δ 8.57 (s, 1H, indole C2-H), 8.18 (d, *J* = 7.9 Hz, 1H, indole C4-H), 8.06 – 7.93 (m, 4H, benzene H, indole C7-H and hexenoate C6-H), 7.72 (t, *J* = 7.4 Hz, 1H, benzene H), 7.63 (t, *J* = 7.7 Hz, 2H, benzene H), 7.48 – 7.38 (m, 2H, indole C5-H and C6-H), 7.21 (d, *J* = 16.2 Hz, 1H, hexenoate C5-H), 6.75 (s, 1H, hexenoate C3-H), 4.30 (q, *J* = 7.2 Hz, 2H, *CH_2_*CH_3_), 1.30 (t, *J* = 7.1 Hz, 3H, CH_2_*CH_3_*). ^13^C NMR (101 MHz, DMSO-*d*_6_) δ 185.39, 172.96, 161.62, 136.48, 135.15, 134.93, 134.47, 131.78, 130.10, 127.29, 126.94, 125.90, 124.56, 123.23, 121.65, 118.44, 113.42, 101.38, 62.05, 13.91. Anal. calcd for C_22_H_19_NO_6_S: C, 62.11; H, 4.50; N, 3.29; S, 7.54%. Found: C, 62.01; H, 4.49; N, 3.28; S, 7.53%. MS (ESI) *m/z* calc. [M+H]^+^ for C_22_H_20_NO_6_S ^+^: 426.46; found: 449.46 [M+Na]^+^.

*Ethyl (2Z,5E)-6-(1-(4-cyanobenzyl)-1H-indol-3-yl)-2-hydroxy-4-oxohexa-2,5-dienoate (****6g****)*. Synthesis, analytical, and spectroscopic data are reported in the literature [1].

*Ethyl (2Z,5E)-2-hydroxy-6-(1-(4-hydroxybenzyl)-1H-indol-3-yl)-4-oxohexa-2,5-dienoate (****6h****)*. Synthesis, analytical, and spectroscopic data are reported in the literature [1].

*Ethyl (2Z,5E)-6-(5-chloro-1-(4-fluorobenzyl)-1H-indol-3-yl)-2-hydroxy-4-oxohexa-2,5-dienoate (****6i****)*. Synthesis, analytical, and spectroscopic data are reported in the literature [1].

*(Z)-2-hydroxy-4-(1-(4-methoxybenzyl)-1H-indol-3-yl)-4-oxobut-2-enoic acid (****7a****)*. Compound **7a** was prepared from **8a** by means of GP-D; 20 min; DMF/H_2_O; 52% as a yellow solid; decomposes at 155 °C; IR ν OH 2945, CO acid 1705, CO ketone 1609 cm^-1^; ^1^H NMR (400 MHz, DMSO-*d*_6_) δ 15.34 (bs, 1H, OH), 13.78 (bs, 1H, OH), 8.97 (s, 1H, indole C2-H), 8.24 – 8.22 (m, 1H, indole C4-H), 7.65 – 7.63 (m, 1H, indole C7-H), 7.35 (d, *J* = 8.5 Hz, 2H, benzene H), 7.28 – 7.26 (m, 2H, indole C5-H and C6-H), 7.00 (s, 1H, butenoate C3-H), 6.89 (d, *J* = 8.6 Hz, 2H, benzene H), 5.44 (s, 2H, CH_2_), 3.70 (s, 3H, CH_3_). ^13^C NMR (101 MHz, DMSO-*d*_6_) δ 189.01, 163.75, 162.73, 158.91, 138.46, 136.86, 129.06, 128.52, 125.89, 123.59, 122.87, 121.80, 114.14, 114.09, 111.74, 100.50, 55.08, 49.60. Anal. calcd for C_20_H_17_NO_5_: C, 68.37; H, 4.88; N, 3.99%. Found: C, 68.13; H, 4.89; N, 3.98%. MS (ESI) *m/z* calc. [M+H]^+^ for C_20_H_18_NO_5_^+^: 352.36; found: 352.40 and 374.40 [M+Na]^+^.

*(Z)-2-hydroxy-4-(1-(4-methylbenzyl)-1H-indol-3-yl)-4-oxobut-2-enoic acid (****7b****)*. Compound **7b** was prepared from **8b** by means of GP-D; 15 min; methanol; 48% as a red solid; 112 - 115 °C; IR ν OH 2924, CO acid 1707, CO ketone 1605 cm^-1^; ^1^H NMR (400 MHz, DMSO-*d*_6_) δ 15.33 (bs, 1H, OH), 13.79 (bs, 1H, OH), 8.97 (s, 1H, indole C2-H), 8.24 – 8.22 (m, 1H, indole C4-H), 7.61 – 7.59 (m, 1H, indole C7-H), 7.28 – 7.13 (m, 6H, benzene H and indole C5-H and C6-H), 7.00 (s, 1H, butenoate C3-H), 5.47 (s, 2H, CH_2_), 2.24 (s, 3H, CH_3_). ^13^C NMR (101 MHz, DMSO-*d*_6_) δ 189.25, 163.96, 162.89, 138.81, 137.36, 137.13, 133.80, 129.48, 129. 43, 127.71, 126.10, 123.83, 123.10, 122.03, 114.39, 111.92, 100.75, 50.11, 20.87. Anal. calcd for C_20_H_17_NO_4_: C, 71.63; H, 5.11; N, 4.18%. Found: C, 71.53; H, 5.12; N, 4.17%. MS (ESI) *m/z* calc. [M+H]^+^ for C_20_H_18_NO_4_^+^: 336.36; found: 358.39 [M+Na]^+^.

*(Z)-2-hydroxy-4-(1-(4-isopropylbenzyl)-1H-indol-3-yl)-4-oxobut-2-enoic acid (****7c****)*. Compound **7c** was prepared from **8c** by means of GP-D; 30 min; methanol; 57% as a yellow solid; decomposes at 135 °C; IR ν OH 3236, CO acid 1741, CO ketone 1592 cm^-1^; ^1^H NMR (400 MHz, DMSO-*d*_6_) δ 15.15 (bs, 1H, OH), 8.99 (s, 1H, indole C2-H), 8.25 – 8.22 (m, 1H, indole C4-H), 7.66 – 7.63 (m, 1H, indole C7-H), 7.31 – 7.19 (m, 6H, benzene H and indole C5-H and C6-H), 7.00 (s, 1H, butenoate C3-H), 5.47 (s, 2H, CH_2_), 2.86 – 2.79 (m, 1H, CH), 1.14 (d, *J* = 6.8 Hz, 6H, CH_3_). ^13^C NMR (101 MHz, DMSO-*d*_6_) δ 189.06, 163.76, 162.83, 148.07, 138.56, 136.96, 134.09, 127.53, 126.63, 125.85, 123.65, 122.88, 121.83, 114.23, 111.69, 100.50, 49.81, 33.10, 23.78. Anal. calcd for C_22_H_21_NO_4_: C, 72.71; H, 5.82; N, 3.85%. Found: C, 72.61; H, 5.83; N, 3.84%. MS (ESI) *m/z* calc. [M+H]^+^ for C_22_H_22_NO_4_^+^: 363.41; found: 386.79 [M+Na]^+^.

*(Z)-4-(1-(4-fluorobenzyl)-1H-indol-3-yl)-2-hydroxy-4-oxobut-2-enoic acid (****7d****)*. Compound **7d** was prepared from **8d** by means of GP-D; 1 h; 70% as a white solid. Analytical and spectroscopic data are in agreement with literature [2].

*(Z)-2-hydroxy-4-oxo-4-(1-(4-(trifluoromethoxy)benzyl)-1H-indol-3-yl)but-2-enoic acid (****7e****)*. Compound **7e** was prepared from **8e** by means of GP-D; 15 min; DMF/H_2_O; 87% as a yellow solid; 174 - 176 °C; IR ν OH 3334, CO acid 1747, CO ketone 1533 cm^-1^; ^1^H NMR (400 MHz, DMSO-*d*_6_) δ 13.73 (bs, 1H, OH), 9.01 (s, 1H, indole C2-H), 8.26 – 8.24 (m, 1H, indole C4-H), 7.66 – 7.62 (m, 1H, indole C7-H), 7.48 (d, *J* = 8.3 Hz, 2H, benzene H), 7.34 (d, *J* = 8.3 Hz, 2H, benzene H), 7.30 – 7.28 (m, 2H, indole C5-H and C6-H), 7.00 (s, 1H, butenoate C3-H), 5.58 (s, 2H, CH_2_). ^13^C NMR (101 MHz, DMSO-*d*_6_) δ 189.13, 163.72, 162.69, 161.06, 147.86, 138.65, 136.91, 136.19, 129.41, 125.84, 123.82, 123.03, 121.90, 121.39, 114.46, 111.58, 100.57, 49.19. Anal. calcd for C_20_H_14_F_3_NO_5_: C, 59.27; H, 3.48; N, 3.46%. Found: C, 59.05; H, 3.49; N, 3.45%. MS (ESI) *m/z* calc. [M+H]^+^ for C_20_H_15_F_3_NO_5_^+^: 406.33; found: 429.39 [M+Na]^+^.

*(Z)-4-(1-benzyl-1H-indol-3-yl)-2-hydroxy-4-oxobut-2-enoic acid (****7j****)*. Compound **7j** was prepared from **8j** by means of GP-D; 1 h; 79% as a green solid. Analytical and spectroscopic data are in agreement with literature [2].

*Ethyl (Z)-2-hydroxy-4-(1-(4-methoxybenzyl)-1H-indol-3-yl)-4-oxobut-2-enoate (****8a****)*. Compound **8a** was prepared from **13a** by means of GP-C; 2.5 h; washed with isopropanol; 80% as a green solid; 147 - 149 °C; IR ν CO ester 1719, CO ketone 1612 cm^-1^; ^1^H NMR (400 MHz, DMSO-*d*_6_) δ 9.00 (s, 1H, indole C2-H), 8.24 – 8.21 (m, 1H, indole C4-H), 7.65 – 7.63 (m, 1H, indole C7-H), 7.35 – 7.27 (m, 4H, benzene H and indole C5-H and C6-H), 7.00 (s, 1H, butenoate C3-H), 6.89 (d, *J* = 8.5 Hz, 2H, benzene H), 5.46 (s, 2H, CH_2_), 4.32 (q, *J* = 7.1 Hz, 2H, *CH_2_*CH_3_), 3.70 (s, 3H, CH_3_), 1.32 (t, *J* = 7.1 Hz, 3H, CH_2_*CH_3_*). ^13^C NMR (101 MHz, DMSO-*d*_6_) δ 188.76, 162.28, 161.87, 158.91, 138.72, 136.91, 129.01, 128.48, 125.87, 123.69, 122.97, 121.82, 114.09, 114.02, 111.81, 100.60, 61.85, 55.09, 49.63, 14.00. Anal. calcd for C_22_H_21_NO_5_: C, 69.65; H, 5.58; N, 3.69%. Found: C, 69.76; H, 5.57; N, 3.68%. MS (ESI) *m/z* calc. [M+H]^+^ for C_22_H_22_NO_5_^+^: 379.41; found: 403.76 [M+Na]^+^.

*Ethyl (Z)-2-hydroxy-4-(1-(4-methylbenzyl)-1H-indol-3-yl)-4-oxobut-2-enoate (****8b****)*. Compound **8b** was prepared from **13b** by means of GP-C; 1.5 h; washed with isopropanol; 40% as a yellow solid; decomposes at 150 °C; IR ν CO ester 1728, CO ketone 1627 cm^-1^; ^1^H NMR (400 MHz, DMSO-*d*_6_) δ 9.01 (s, 1H, indole C2-H), 8.25 – 8.23 (m, 1H, indole C4-H), 7.62 – 7.60 (m, 1H, indole C7-H), 7.26 – 7.14 (m, 6H, benzene H and indole C5-H and C6-H), 7.01 (s, 1H, butenoate C3-H), 5.50 (s, 2H, CH_2_), 4.33 (q, *J* = 7.1 Hz, 2H, *CH_2_*CH_3_), 2.26 (s, 3H, CH_3_), 1.34 (t, *J* = 7.1 Hz, 3H, CH_2_*CH_3_*). ^13^C NMR (101 MHz, DMSO-*d*_6_) δ 188.80, 162.28, 138.86, 137.16, 136.97, 133.58, 129.28, 127.45, 125.86, 123.72, 122.99, 121.83, 114.06, 111.79, 100.63, 61.87, 49.93, 20.67, 14.00. Anal. calcd for C_22_H_21_NO_4_: C, 72.71; H, 5.82; N, 3.85%. Found: C, 72.54; H, 5.83; N, 3.86%. MS (ESI) *m/z* calc. [M+H]^+^ for C_22_H_22_NO_4_^+^: 364.41; found: 387.75 [M+Na]^+^ and 418.45 [M+Na+CH_3_OH]^+^.

*Ethyl (Z)-2-hydroxy-4-(1-(4-isopropylbenzyl)-1H-indol-3-yl)-4-oxobut-2-enoate (****8c****)*. Compound **8c** was prepared from **13c** by means of GP-C; 1.5 h; washed with methanol; 55% as a yellow solid; decomposes at 120 °C; IR ν OH 2963, CO ester 1721, CO ketone 1626 cm^-1^; ^1^H NMR (400 MHz, DMSO-*d*_6_) δ 15.48 (bs, 1H, OH), 9.01 (s, 1H, indole C2-H), 8.24 – 8.22 (m, 1H, indole C4-H), 7.65 – 7.63 (m, 1H, indole C7-H), 7.30 – 7.19 (m, 6H, benzene H and indole C5-H and C6-H), 7.01 (s, 1H, butenoate C3-H), 5.49 (s, 2H, CH_2_), 4.32 (q, *J* = 7.1 Hz, 2H, *CH_2_*CH_3_), 2.83 (m, 1H, CH), 1.32 (t, *J* = 7.1 Hz, 3H, CH_2_*CH_3_*), 1.14 (d, *J* = 6.9 Hz, 6H, CH_3_). ^13^C NMR (101 MHz, DMSO-*d*_6_) δ 188.79, 162.26, 161.87, 148.07, 138.81, 137.00, 134.04, 127.47, 126.63, 125.81, 123.74, 122.98, 121.83, 114.09, 111.74, 100.61, 61.84, 49.82, 33.09, 23.77, 13.98. Anal. calcd for C_24_H_25_NO_4_: C, 73.64; H, 6.44; N, 3.58%. Found: C, 73.80; H, 6.45; N, 3.59%. MS (ESI) *m/z* calc. [M+H]^+^ for C_24_H_26_NO_4_^+^: 391.47; found: 415.81 [M+Na]^+^ and 446.82 [M+Na+CH_3_OH]^+^.

*Ethyl (Z)-4-(1-(4-fluorobenzyl)-1H-indol-3-yl)-2-hydroxy-4-oxobut-2-enoate (****8d****)*. Compound **8d** was prepared from **13d** by means of GP-C; 1 h; 97% as a white solid. Analytical and spectroscopic data are in agreement with literature [2].

*Ethyl (Z)-2-hydroxy-4-oxo-4-(1-(4-(trifluoromethoxy)benzyl)-1H-indol-3-yl)but-2-enoate (****8e****)*. Compound **8e** was prepared from **13e** by means of GP-C; 45 min; washed with isopropanol; 82% as a yellow solid; decomposes at 150 °C; IR ν CO ester 1724, CO ketone 1628 cm^-1^; ^1^H NMR (400 MHz, DMSO-*d*_6_) δ 9.03 (s, 1H, indole C2-H), 8.26 – 8.24 (m, 1H, indole C4-H), 7.66 – 7.62 (m, 1H, indole C7-H), 7.47 (d, *J* = 8.4 Hz, 2H, benzene H), 7.35 (d, *J* = 8.2 Hz, 2H, benzene H), 7.30 – 7.28 (m, 2H, indole C5-H and C6-H), 7.01 (s, 1H, butenoate C3-H), 5.59 (s, 2H, CH_2_), 4.32 (q, *J* = 7.1 Hz, 2H, *CH_2_*CH_3_), 1.32 (t, *J* = 7.1 Hz, 3H, CH_2_*CH_3_*). ^13^C NMR (101 MHz, DMSO-*d*_6_) δ 188.87, 162.23, 161.85, 147.84, 138.87, 136.94, 136.15, 129.34, 125.79, 123.88, 123.10, 121.89, 121.37, 118.74, 114.31, 111.62, 100.63, 61.86, 49.18, 13.98. Anal. calcd for C_22_H_18_F_3_NO_5_: C, 60.97; H, 4.19; N, 3.23%. Found: C, 60.77; H, 4.18; N, 3.24%. MS (ESI) *m/z* calc. [M+H]^+^ for C_22_H_19_F_3_NO_5_^+^: 433.38; found: 457.42 [M+Na]^+^.

*Ethyl (Z)-4-(1-benzyl-1H-indol-3-yl)-2-hydroxy-4-oxobut-2-enoate (****8j****)*. Compound **8j** was prepared from **13j** by means of GP-C; 1.5 h; 97% as a yellow solid. Analytical and spectroscopic data are in agreement with literature [2].

*(2Z,5E)-6-(1-((E)-but-2-en-1-yl)-1H-indol-3-yl)-2-hydroxy-4-oxohexa-2,5-dienoic acid (****9a****)*. Synthesis, analytical, and spectroscopic data are reported in the literature. literature [1].

*(2Z,5E)-2-hydroxy-6-(1-(2-methylallyl)-1H-indol-3-yl)-4-oxohexa-2,5-dienoic acid (****9b****)*. Synthesis, analytical, and spectroscopic data are reported in the literature [1].

*(2Z,5E)-2-hydroxy-6-(1-(2-methylprop-1-en-1-yl)-1H-indol-3-yl)-4-oxohexa-2,5-dienoic acid (****9c****)*. Synthesis, analytical, and spectroscopic data are reported in the literature [1].

*(2Z,5E)-6-(1-ethyl-1H-indol-3-yl)-2-hydroxy-4-oxohexa-2,5-dienoic acid (****9d****)*. Compound **9d** was prepared from **10d** by means of GP-D; 1.5 h; DMF/H_2_O; 82% as a red solid; 128 - 130 °C; IR ν OH 2980, CO acid 1725 cm^-1^; ^1^H NMR (400 MHz, CD_3_OD) δ 14.40 (bs, 1H, OH), 8.16 – 8.10 (m, 3H, indole C2-H and C4-H), 8.00 (s, 1H, hexenoate C4-H), 7.76 (d, *J* = 9.7 Hz, 1H, indole C4-H), 7.38 – 7.30 (m, 2H, indole C5-H and C6-H), 6.96 – 6.60 (m, 2H, hexenoate C3-H and C5-H), 4.31 (q, *J* = 6.9 Hz, 2H, *CH_2_*CH_3_), 1.45 (t, *J* = 7.2 Hz, 3H, CH_2_*CH_3_*). ^13^C NMR (101 MHz, CD_3_OD) δ 197.18, 179.49, 171.58, 147.98, 146.79, 146.02, 134.97, 132.59, 131.25, 130.44, 126.23, 121.78, 120.54, 110.62, 50.48, 24.50. Anal. calcd for C_16_H_15_NO_4_: C, 67.36; H, 5.30; N, 4.91%. Found: C, 67.47; H, 5.29; N, 4.90%. MS (ESI) *m/z* calc. [M+H]^+^ for C_16_H_16_NO_4_^+^: 286.30; found: 286.41 and 240.71 [M-COOH]^+^.

*(2Z,5E)-6-(1-butyl-1H-indol-3-yl)-2-hydroxy-4-oxohexa-2,5-dienoic acid (****9e****)*. Synthesis, analytical, and spectroscopic data are reported in the literature [1].

*(2Z,5E)-2-hydroxy-6-(1-isopentyl-1H-indol-3-yl)-4-oxohexa-2,5-dienoic acid (****9f****)*. Synthesis, analytical, and spectroscopic data are reported in the literature [1].

*Ethyl (2Z,5E)-6-(1-((E)-but-2-en-1-yl)-1H-indol-3-yl)-2-hydroxy-4-oxohexa-2,5-dienoate (****10a****)*. Synthesis, analytical, and spectroscopic data are reported in the literature [1].

*Ethyl (2Z,5E)-2-hydroxy-6-(1-(2-methylallyl)-1H-indol-3-yl)-4-oxohexa-2,5-dienoate (****10b****)*. Synthesis, analytical, and spectroscopic data are reported in the literature [1].

*Ethyl (2Z,5E)-2-hydroxy-6-(1-(2-methylprop-1-en-1-yl)-1H-indol-3-yl)-4-oxohexa-2,5-dienoate (****10c****)*. Synthesis, analytical, and spectroscopic data are reported in the literature [1].

*Ethyl (2Z,5E)-6-(1-ethyl-1H-indol-3-yl)-2-hydroxy-4-oxohexa-2,5-dienoate (****10d****)*. Compound **10d** was prepared from **15d** by means of GP-C; 1.5 h; toluene; 62% as a red solid; 126 - 128 °C; IR ν CO ester 1725, CO ketone 1587 cm^-1^; ^1^H NMR (400 MHz, DMSO-*d*_6_) δ 15.60 (bs, 1H, OH), 8.20 – 8.16 (m, 2H, indole C2-H and C4-H), 8.08 (d, 1H, *J* = 15.6 Hz, hexenoate C6-H), 7.66 (d, *J* = 8.1 Hz, 1H, indole C3-H), 7.38 – 7.29 (m, 2H, indole C5-H and C6-H), 6.97 (d, *J* = 15.5 Hz, 1H, hexenoate C5-H), 6.73 (s, 1H, hexenoate C3-H), 4.36-4.28 (m, 4H, *CH_2_*CH_3_), 1.47-1.33 (m, 6H, CH_2_*CH_3_*). ^13^C NMR (101 MHz, DMSO-*d*_6_) δ 187.69, 170.01, 162.10, 138.50, 137.31, 136.54, 125.49, 123.11, 121.77, 120.96, 116.75, 112.30, 111.06, 101.14, 61.81, 41.00, 15.02, 13.94. Anal. calcd for C_18_H_19_NO_4_: C, 69.00; H, 6.11; N, 4.47%. Found: C, 69.09; H, 6.12; N, 4.46%. MS (ESI) *m/z* calc. [M+H]^+^ for C_18_H_20_NO_4_^+^: 314.35; found: 314.42 and 336.79 [M+Na]^+^.

*Ethyl (2Z,5E)-6-(1-butyl-1H-indol-3-yl)-2-hydroxy-4-oxohexa-2,5-dienoate (****10e****)*. Synthesis, analytical, and spectroscopic data are reported in the literature [1].

*Ethyl (2Z,5E)-2-hydroxy-6-(1-isopentyl-1H-indol-3-yl)-4-oxohexa-2,5-dienoate (****10f****)*. Synthesis, analytical, and spectroscopic data are reported in the literature [1].

*1-(4-methylbenzyl)-1H-indole-3-carbaldehyde (****11b****)*. Compound **11b** was prepared from 1*H*-indole-3-carboxaldehyde by means of GP-A using 4-methylbenzyl bromide as alkylating agent; 15 h; 95% as a pink solid. Analytical and spectroscopic data are in agreement with literature [3].

*1-(4-isopropylbenzyl)-1H-indole-3-carbaldehyde (****11c****)*. Compound **11c** was prepared from 1*H*-indole-3-carboxaldehyde by means of GP-A using 4-isopropylbenzyl bromide as alkylating agent; 3 h; 74% as a pink solid. Analytical and spectroscopic data are in agreement with literature [4].

*1-(4-(trifluoromethoxy)benzyl)-1H-indole-3-carbaldehyde (****11e****)*. Compound **11e** was prepared from 1*H*-indole-3-carboxaldehyde by means of GP-A using 4-(trifluoromethoxy)benzyl bromide as alkylating agent; 30 min; toluene; 76% as a pink solid; 127 - 130 °C; IR ν CO 1645 cm^-1^; ^1^H NMR (400 MHz, DMSO-*d*_6_) δ 9.95 (s, 1H, CHO), 8.49 (s, 1H, indole C2-H), 8.12 (d, *J* = 7.2 Hz, 1H, indole C4-H), 7.60 (d, *J* = 7.2 Hz, 1H, indole C7-H), 7.42 (d, *J* = 8.6 Hz, 2H, benzene H), 7.35 (d, *J* = 8.6 Hz, 2H, benzene H), 7.28 – 7.23 (m, 2H, indole C5-H and C6-H), 5.60 (s, 2H, CH_2_). Anal. calcd for C_17_H_12_F_3_NO_2_: C, 63.95; H, 3.79; N, 4.39%. Found: C, 64.11; H, 3.78; N, 4.38%.

*(E)-4-(1-(4-methylbenzyl)-1H-indol-3-yl)but-3-en-2-one (****12b****)*. Compound **12b** was prepared from **11b** by means of GP-B; cyclohexane; 100% as a green solid; 113 - 115 °C; IR ν CO 1671 cm^-1^; ^1^H NMR (400 MHz, DMSO-*d*_6_) δ 8.10 (s, 1H, indole C2-H), 7.94 (d, *J* = 7.0 Hz, 1H, indole C4-H), 7.80 (d, *J* = 16.2 Hz, 1H, butenoate C4-H), 7.55 (d, *J* = 6.8 Hz, 1H, indole C7-H), 7.23 – 7.11 (m, 6H, benzene H and indole C5-H and C6-H), 6.69 (d, *J* = 16.3 Hz, 1H, butenoate C3-H), 5.41 (s, 2H, CH_2_), 2.30 (s, 3H, butenoate C1-H), 2.24 (s, 3H, CH_3_). Anal. calcd for C_20_H_19_NO: C, 83.01; H, 6.62; N, 4.84%. Found: C, 83.13; H, 6.61; N, 4.85%.

*(E)-4-(1-(4-isopropylbenzyl)-1H-indol-3-yl)but-3-en-2-one (****12c****)*. Compound **12c** was prepared from **11c** by means of GP-B; cyclohexane; 72% as a yellow solid; 85 - 88 °C; IR ν CO 1669 cm^-1^; ^1^H NMR (400 MHz, DMSO-*d*_6_) δ 8.12 (s, 1H, indole C2-H), 7.94 (d, *J* = 7.5 Hz, 1H, indole C4-H), 7.81 (d, *J* = 16.3 Hz, 1H, butenoate C4-H), 7.58 (d, *J* = 7.9 Hz, 1H, indole C7-H), 7.23 – 7.19 (m, 6H, benzene H and indole C5-H and C6-H), 6.69 (d, *J* = 16.3 Hz, 1H, butenoate C3-H), 5.42 (s, 2H, CH_2_), 2.84 – 2.80 (m, 1H, CH), 2.30 (s, 3H, butenoate C1-H), 1.15 (6H, dd, *J* = 6.9, 1.6 Hz, CH_3_). Anal. calcd for C_22_H_23_NO: C, 83.24; H, 7.30; N, 4.41; %. Found: C, 83.10; H, 7.31; N, 4.42%.

*(E)-4-(1-(4-(trifluoromethoxy)benzyl)-1H-indol-3-yl)but-3-en-2-one (****12e****)*. Compound **12e** was prepared from **12e** by means of GP-B; toluene; 97% as a yellow solid; 131 - 134 °C; IR ν CO 1669 cm^-1^; ^1^H NMR (400 MHz, DMSO-*d*_6_) δ 8.14 (s, 1H, indole C2-H), 7.96 (d, *J* = 7.1 Hz, 1H, indole C4-H), 7.82 (d, *J* = 16.3 Hz, 1H, butenoate C4-H), 7.57 (d, *J* = 7.2 Hz, 1H, indole C7-H), 7.38 – 7.32 (m, 4H, benzene H), 7.25 – 7.19 (m, 2H, indole C5-H and C6-H), 6.72 (d, *J* = 16.3 Hz, 1H, butenoate C3-H), 5.53 (s, 2H, CH_2_), 2.30 (s, 3H, butenoate C1-H). Anal. calcd for C_20_H_16_F_3_NO_2_: C, 66.85; H, 4.49; N, 3.90 %. Found: C, 66.80; H, 4.48; N, 3.91%.

*(E)-4-(1-(phenylsulfonyl)-1H-indol-3-yl)but-3-en-2-one (****12f****)*. Compound **12f** was prepared from (*E*)-4-(1*H*-indol-3-yl)but-3-en-2-one [5] by means of GP-A using benzenesulfonyl chloride as alkylating agent; 1 h; toluene; 28% as a black wax; 133 - 135 °C; IR ν SO_2_ 1362 and 1173 cm^-1^; ^1^H NMR (400 MHz, DMSO-*d*_6_) δ 8.47 8.05 – 7.98 (m, 4H, benzene H and indole C4-H and C7-H), 7.78 – 7.70 (m, 2H, butenoate C4-H and benzene H), 7.62 (7, *J* = 8.0 Hz, 2H, benzene H), 7.46 – 7.35 (m, 2H, indole C5-H and C6-H), 6.93 (d, *J* = 16.3 Hz, 1H, butenoate C3-H), 2.34 (s, 3H, butenoate C1-H). Anal. calcd for C_18_H_15_NO_3_S: C, 66.44; H, 4.65; N, 4.30; S, 9.85%. Found: C, 66.40; H, 4.64; N, 4.29; S, 9.86%.

*1-(1-(4-methoxybenzyl)-1H-indol-3-yl)ethan-1-one (****13a****)*. Compound **13a** was prepared from 3-acetylindole by means of GP-A using 4-methoxybenzyl bromide as alkylating agent; 3 h; washed with diisopropyl ether; 88% as a white solid; 150 - 153 °C; IR ν CO 1630 cm^-1^; ^1^H NMR (400 MHz, DMSO-*d*_6_) δ 8.52 (s, 1H, indole C2-H), 8.19 – 8.16 (m, 1H, indole C4-H), 7.56 – 7.53 (m, 1H, indole C7-H), 7.28 (d, *J* = 8.0 Hz, 2H, benzene H), 7.23 – 7.16 (m, 2H, indole C5-H and C6-H), 6.88 (d, *J* = 8.0 Hz, 2H, benzene H), 5.41 (s, 2H, CH_2_), 3.70 (s, 3H, CH_3_), 2.45 (s, 3H, COCH_3_). Anal. calcd for C_18_H_17_NO_2_: C, 77.40; H, 6.13; N, 5.01%. Found: C, 77.33; H, 6.12; N, 5.00%.

*1-(1-(4-methylbenzyl)-1H-indol-3-yl)ethan-1-one (****13b****)*. Compound **13b** was prepared from 3-acetylindole by means of GP-A using 4-methylbenzyl bromide as alkylating agent; 3 h; washed with diisopropyl ether; 82% as a white solid; 185 - 187 °C; IR ν CO 1629 cm^-1^; ^1^H NMR (400 MHz, DMSO-*d*_6_) δ 8.52 (s, 1H, indole C2-H), 8.19 – 8.16 (m, 1H, indole C4-H), 7.52 – 7.50 (m, 1H, indole C7-H), 7.21 – 7.12 (m, 6H, benzene H and indole C5-H and C6-H), 5.44 (s, 2H, CH_2_), 2.45 (s, 3H, COCH_3_), 2.43 (s, 3H, CH_3_). Anal. calcd for C_18_H_17_NO: C, 82.10; H, 6.51; N, 5.32%. Found: C, 82.20; H, 6.50; N, 5.31%.

*1-(1-(4-isopropylbenzyl)-1H-indol-3-yl)ethan-1-one (****13c****)*. Compound **13c** was prepared from 3-acetylindole by means of GP-A using 4-isopropylbenzyl bromide as alkylating agent; 15 h; cyclohexane; 100% as a white solid; 150 - 153 °C; IR ν CO 1630 cm^-1^; ^1^H NMR (400 MHz, DMSO-*d*_6_) δ 8.54 (s, 1H, indole C2-H), 8.18 (d, *J* = 7.3 Hz, 1H, indole C4-H), 7.55 (d, *J* = 7.7 Hz, 1H, indole C7-H), 7.24 – 7.18 (m, 6H, benzene H and indole C5-H and C6-H), 5.45 (s, 2H, CH_2_), 2.84 – 2.81 (m, 1H, CH), 2.46 (s, 3H, COCH_3_), 1.15 (6H, dd, *J* = 6.9, 1.6 Hz, CH_3_). Anal. calcd for C_20_H_21_NO: C, 82.44; H, 7.26; N, 4.81%. Found: C, 82.50; H, 7.24; N, 4.80%.

*1-(1-(4-fluorobenzyl)-1H-indol-3-yl)ethan-1-one (****13d****)*. Compound **13d** was prepared from 3-acetylindole by means of GP-A using 4-fluorobenzyl bromide as alkylating agent; 30 min; 93% as a yellow solid. Analytical and spectroscopic data are in agreement with literature [2].

*1-(1-(4-(trifluoromethoxy)benzyl)-1H-indol-3-yl)ethan-1-one (****13e****)*. Compound **13e** was prepared from 3-acetylindole by means of GP-A using 4-(trifluoromethoxy)benzyl bromide as alkylating agent; 30 min; washed with boiling ethanol; 100% as a white solid; decomposes at 180 °C; IR ν CO 1626 cm^-1^; ^1^H NMR (400 MHz, DMSO-*d*_6_) δ 8.56 (s, 1H, indole C2-H), 7.20 – 7.18 (m, 1H, indole C4-H), 7.55 – 7.53 (m, 1H, indole C7-H), 7.41 (d, *J* = 8.0 Hz, 2H, benzene H), 7.33 (d, *J* = 8.0 Hz, 2H, benzene H), 7.24 – 7.18 (m, 2H, indole C5-H and C6-H), 5.53 (s, 2H, CH_2_), 2.46 (s, 3H, COCH_3_). Anal. calcd for C_18_H_14_F_3_NO_2_: C, 64.86; H, 4.23; N, 4.20 %. Found: C, 64.91; H, 4.23; N, 4.20 %.

*1-(1-benzyl-1H-indol-3-yl)ethan-1-one (****13j****)*. Compound **13j** was prepared from 3-acetylindole by means of GP-A using benzyl bromide as alkylating agent; 15 h; 79% as a white solid. Analytical and spectroscopic data are in agreement with literature [6].

*Synthesis of 1-ethyl-1H-indole-3-carbaldehyde (****14d****)*. To a solution of indole-3-carboxaldehyde (9.2 mmol) in 20 mL of dry DMF, NaH 60% in mineral oil (14.7 mmol) was added portionwise at 0 °C within 10 minutes [7]. After development of H_2_, the solution was treated with the iodoethane (11 mmol) and the resulting mixture was stirred at room temperature for 1 h. Upon completion the reaction was diluted with water and the solid that formed was filtered under pressure, washed with absolute ethanol and light petroleum ether, giving the pure compound **14d** as a white solid (77% yield). Analytical and spectroscopic data are in agreement with literature [4].

*(E)-4-(1-ethyl-1H-indol-3-yl)but-3-en-2-one (****15d****)*. Compound **15d** was prepared from **14d** by means of GP-B; *n*-hexane/ethyl acetate 2:1; 84% as a yellow oil; IR ν CO 1656 cm^-1^; ^1^H NMR (400 MHz, DMSO-*d*_6_) δ 7.52 (s, 1H, indole C2-H), 8.00-7.98 (m, 1H, indole C4-H), 7.83 (d, *J* = 16.1 Hz, 1H, butenoate C4-H), 7.46-7.43 (m, 1H, indole C7-H), 7.39 – 7.30 (m, 2H, indole C5-H and C6-H), 6.82 (d, *J* = 16.1 Hz, 1H, butenoate C3-H), 4.25 (q, *J* = 7.3 Hz, 2H, *CH_2_*CH_3_), 2.43 (s, 3H, CH_3_), 1.57 (t, *J* = 7.3 Hz, 3H, CH_2_*CH_3_*). Anal. calcd for C_14_H_15_NO: C, 78.84; H, 7.09; N, 6.57%. Found: C, 78.80; H, 7.08; N, 6.56%.

REFERENCES

1. Costi, R.; Cuzzucoli Crucitti, G.; Pescatori, L.; Messore, A.; Scipione, L.; Tortorella, S.; Amoroso, A.; Crespan, E.; Campiglia, P.; Maresca, B.; Porta, A.; Granata, I.; Novellino, E.; Gouge, J.; Delarue, M.; Maga, G.; Di Santo, R. New nucleotide-competitive non-nucleoside inhibitors of terminal deoxynucleotidyl transferase: Discovery, characterization, and crystal structure in complex with the target. *J. Med. Chem.* **2013**, *56*, 7431–7441. doi: 10.1021/jm4010187.
2. Barreca, M. L.; Ferro, S.; Rao, A.; De Luca, L.; Zappalà, M.; Monforte, A. M.; Debyser, Z.; Witvrouw, M.; Chimirri, A. Pharmacophore-based design of HIV-1 integrase strand-transfer inhibitors. *J. Med. Chem.* **2005**, *48(22)*, 7084–7088. doi: 10.1021/jm050549e.
3. Chadha, N.; Silakari, O. Identification of low micromolar dual inhibitors for aldose reductase (ALR2) and poly (ADP-ribose) polymerase (PARP-1) using structure based design approach. *Bioorg. Med. Chem. Lett.* **2017**, *27(11)*, 2324–2330. doi: 10.1016/j.bmcl.2017.04.038.
4. Wang, G.; Li, C.; He, L.; Lei, K.; Wang, F.; Pu, Y.; Yang, Z.; Cao, D.; Ma, L.; Chen, J.; Sang, Y.; Liang, X.; Xiang, M.; Peng, A.; Wei, Y.; Chen, L. Design, synthesis and biological evaluation of a series of pyrano chalcone derivatives containing indole moiety as novel anti-tubulin agents. *Bioorg. Med. Chem.* **2014**, *22(7)*, 2060–2079. doi: 10.1016/j.bmc.2014.02.028.
5. Caballero, E.; Longieras, N.; Zausa, E.; del Rey, B.; Medardeand, M.; Tome, F. Diels–Alder reactivity and some synthetic applications of (*E*)-1-(3-indolyl)-3-*tert*-butyldimethylsiloxy-1,3-butadienes. *Tetrahedron Lett*. **2001**, *42*, 7233–7236. doi: 10.1016/S0040-4039(01)01487-3.
6. Perspicace, E.; Jouan-Hureaux, V.; Ragno, R.; Ballante, F.; Sartini, S.; La Motta, C.; Da Settimo, F.; Chen, B.; Kirsch, G.; Schneider, S.; Faivre, B.; Hesse, S. Design, synthesis and biological evaluation of new classes of thieno[3,2-d]pyrimidinone and thieno[1,2,3]triazine as inhibitor of vascular endothelial growth factor receptor-2 (VEGFR-2). *Eur. J. Med. Chem.* **2013**, *63*, 765–781. doi: 10.1016/j.ejmech.2013.03.022.
7. Cuzzucoli Crucitti, G.; Pescatori, L.; Messore, A.; Madia, V.N.; Pupo, G.; Saccoliti, F.; Scipione, L.; Tortorella, S.; Di Leva, F.S.; Cosconati, S.; Novellino, E.; Debyser, Z.; Christ, F.; Costi, R.; Di Santo, R. Discovery of *N*-aryl-naphthylamines as *in vitro* inhibitors of the interaction between HIV integrase and the cofactor LEDGF/p75. *Eur. J. Med. Chem.* **2015**, *101*, 288-94. doi: 10.1016/j.ejmech.2015.06.036.

**FTIR, ^1^H NMR, ^13^C NMR Spectra**


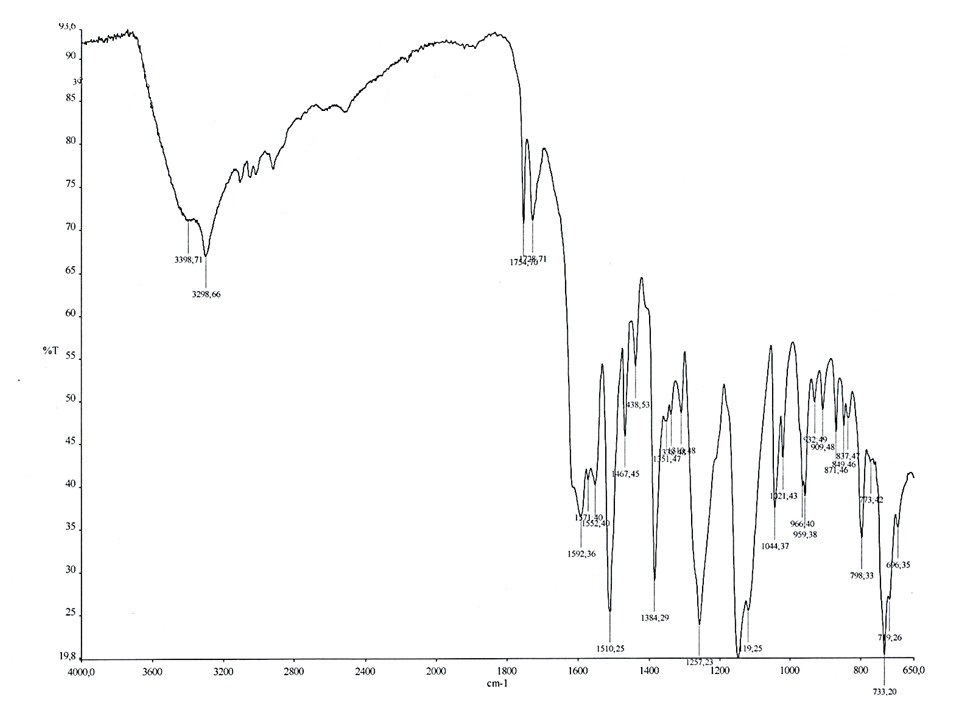


Figure S4. FTIR Spectrum for compound **5b**


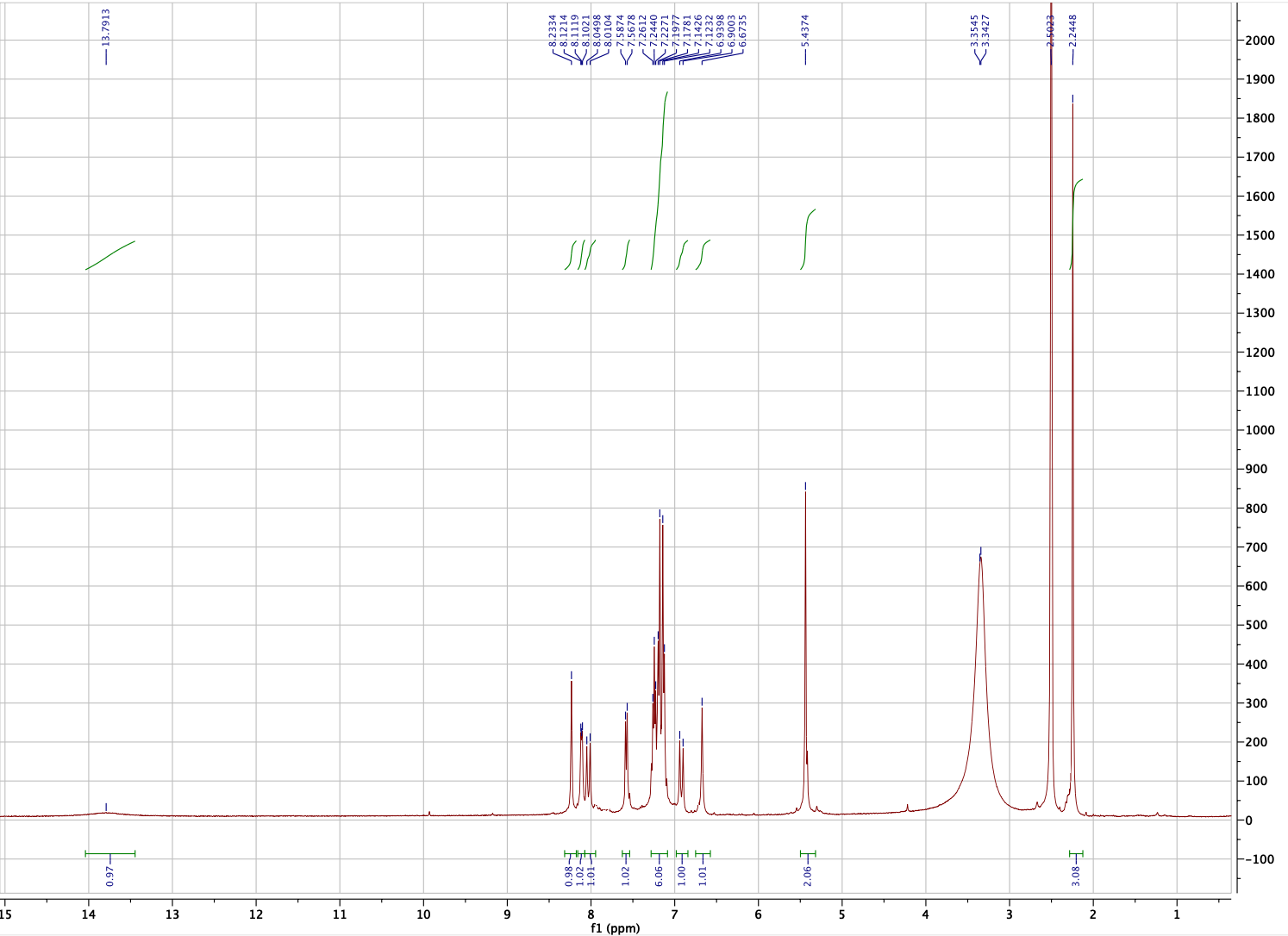


Figure S5. ^1^H NMR Spectrum for compound **5b**
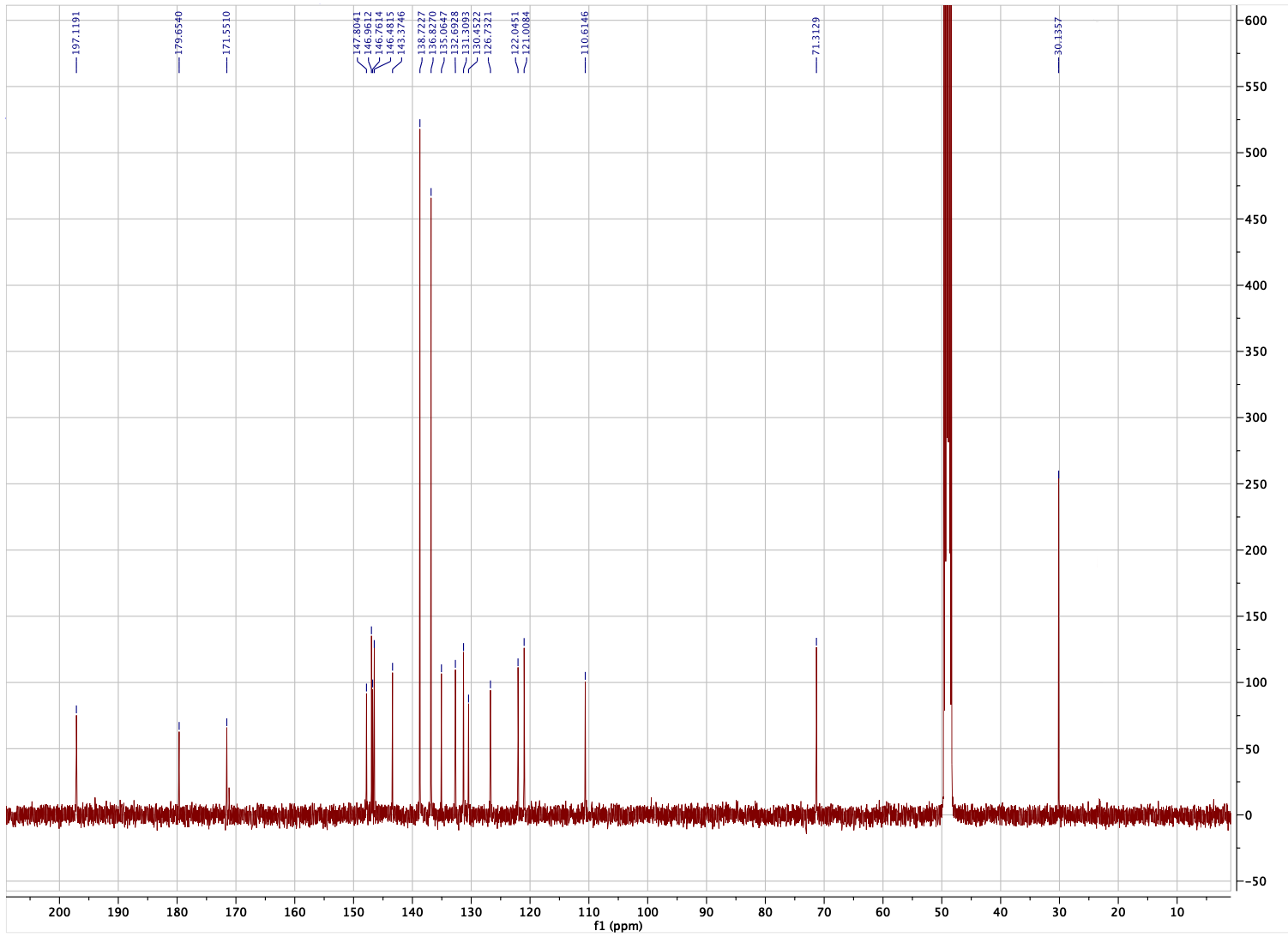


Figure S6. ^13^C NMR Spectrum for compound **5b**
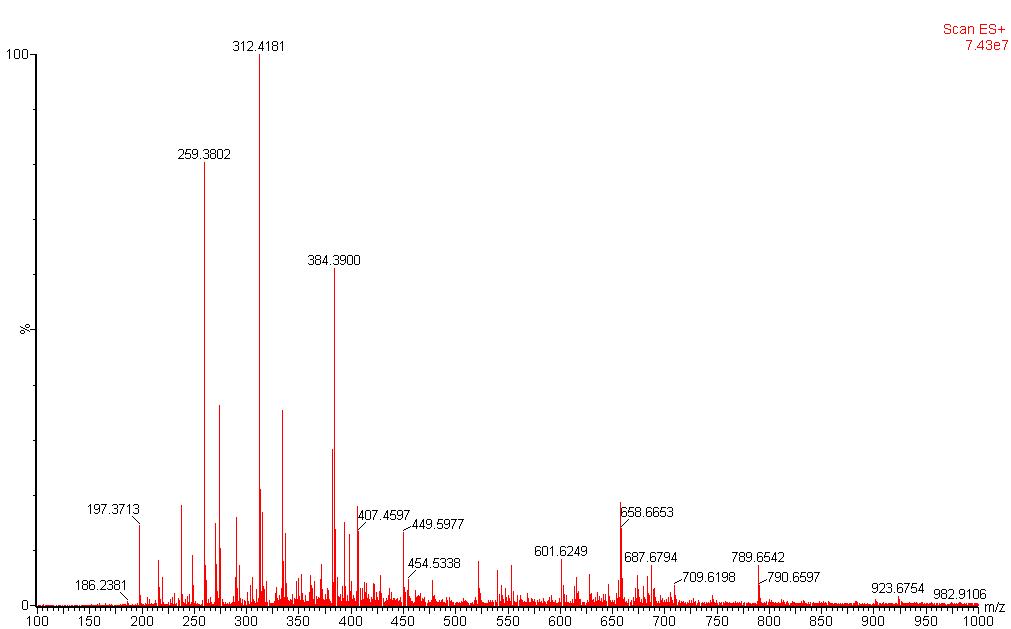


Figure S7. MS (ESI) Spectrum for compound **5b**


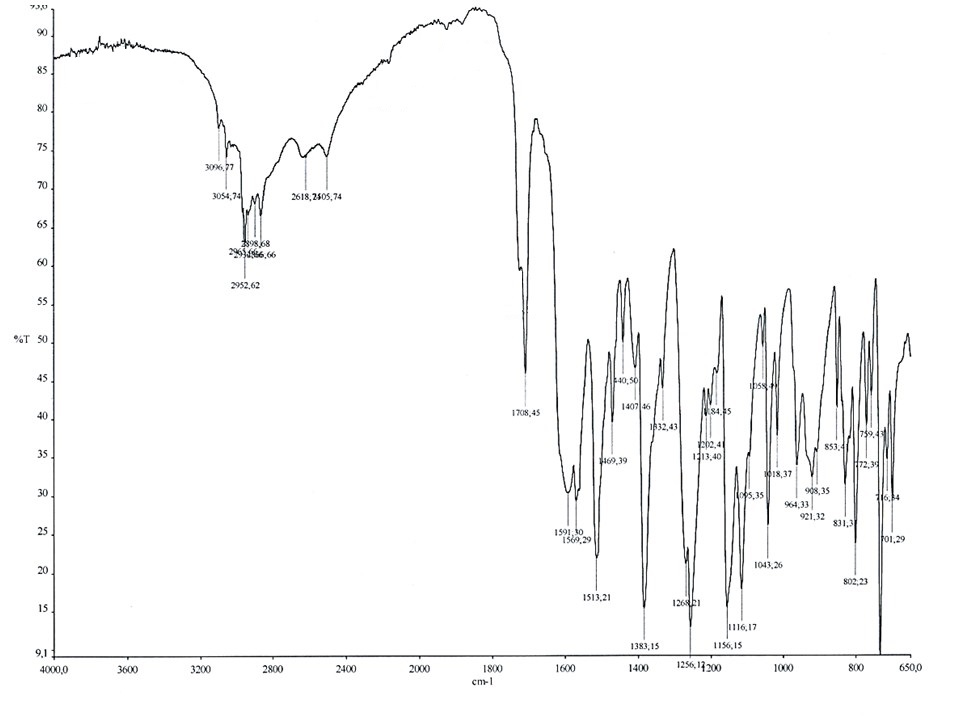


Figure S8. FTIR Spectrum for compound **5c**


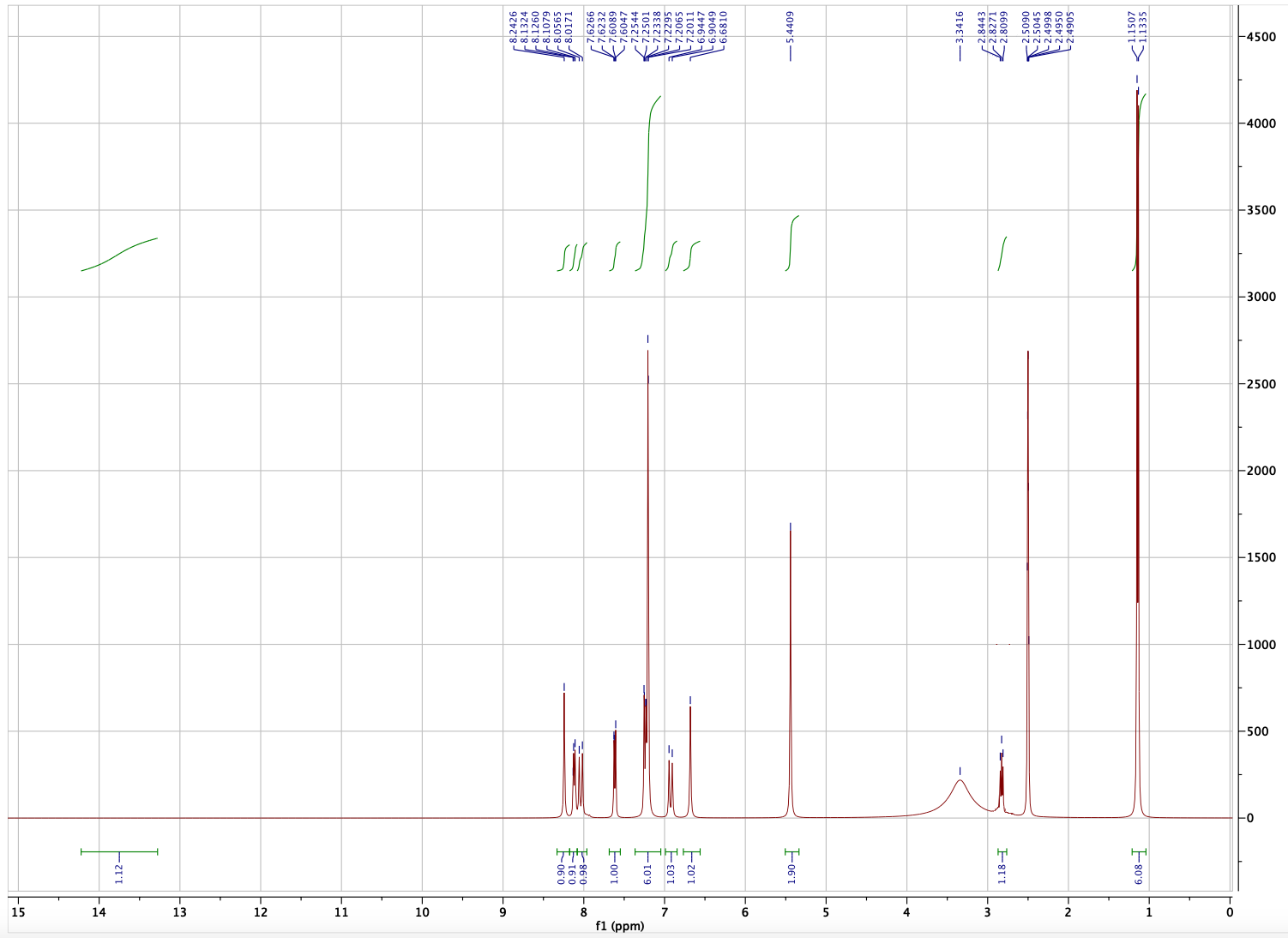


Figure S9. ^1^H NMR Spectrum for compound **5c**


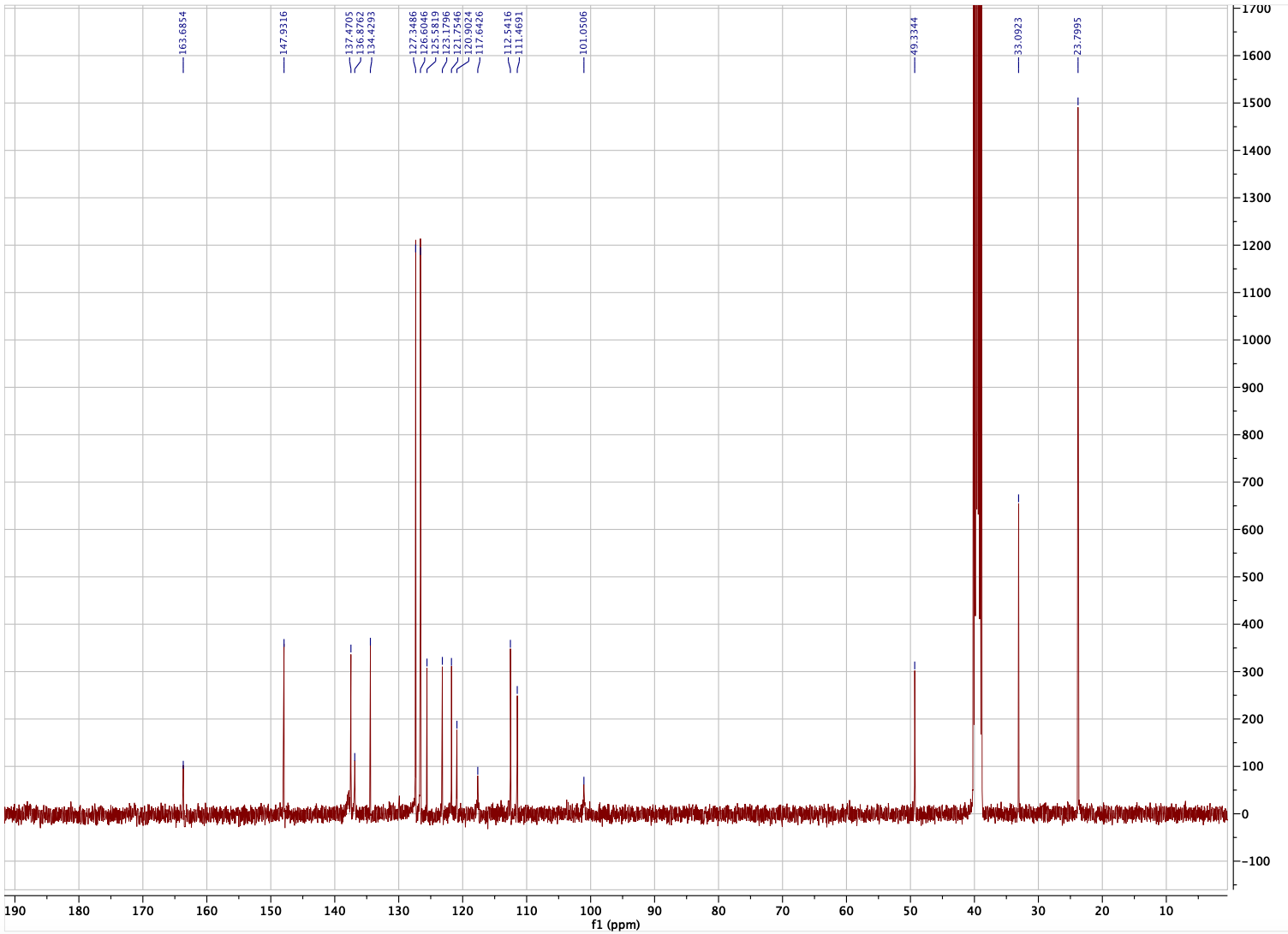


Figure S10. ^13^C NMR Spectrum for compound **5c**
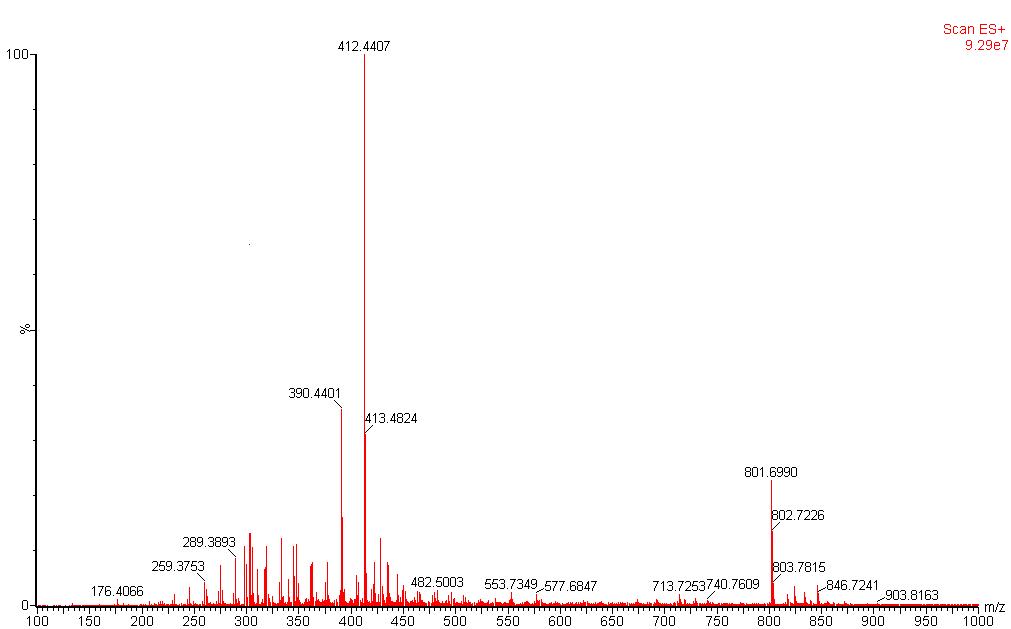


Figure S11. MS (ESI) Spectrum for compound **5c**


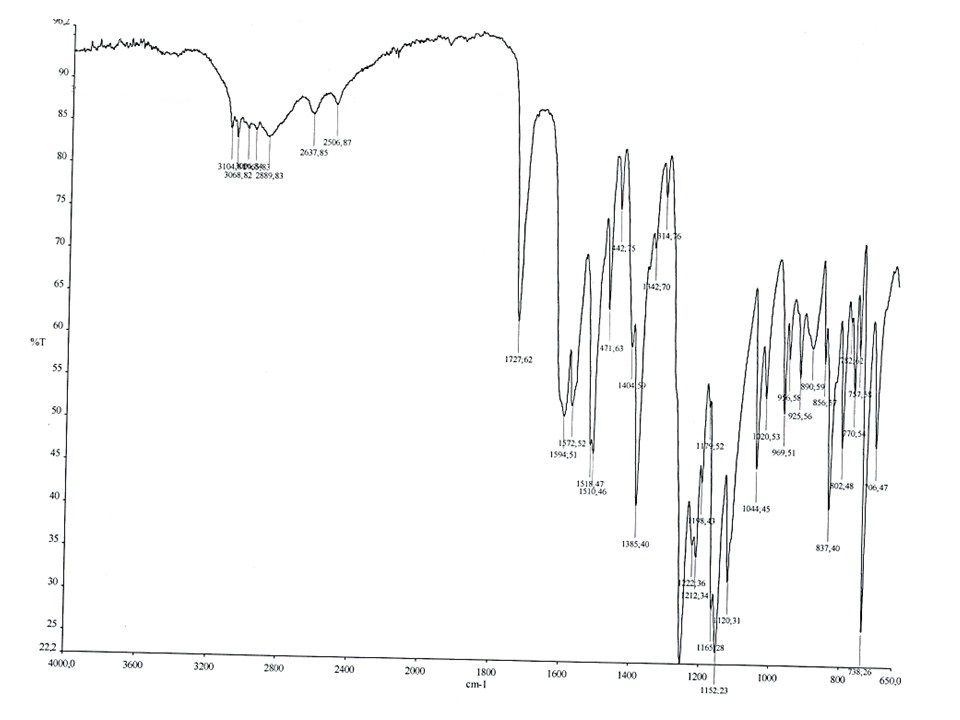


Figure S12. FTIR Spectrum for compound **5e**


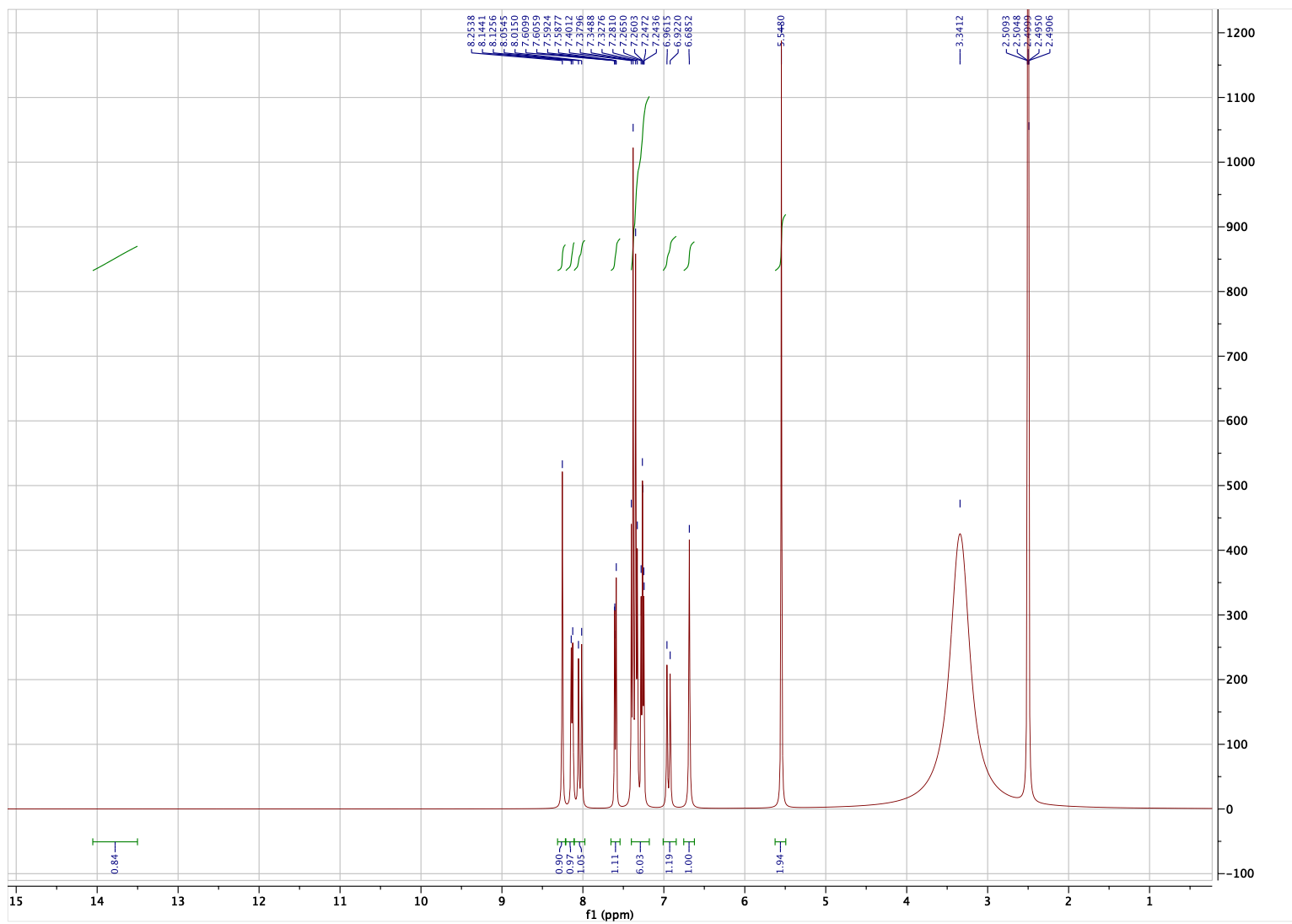


Figure S13. ^1^H NMR Spectrum for compound **5e**


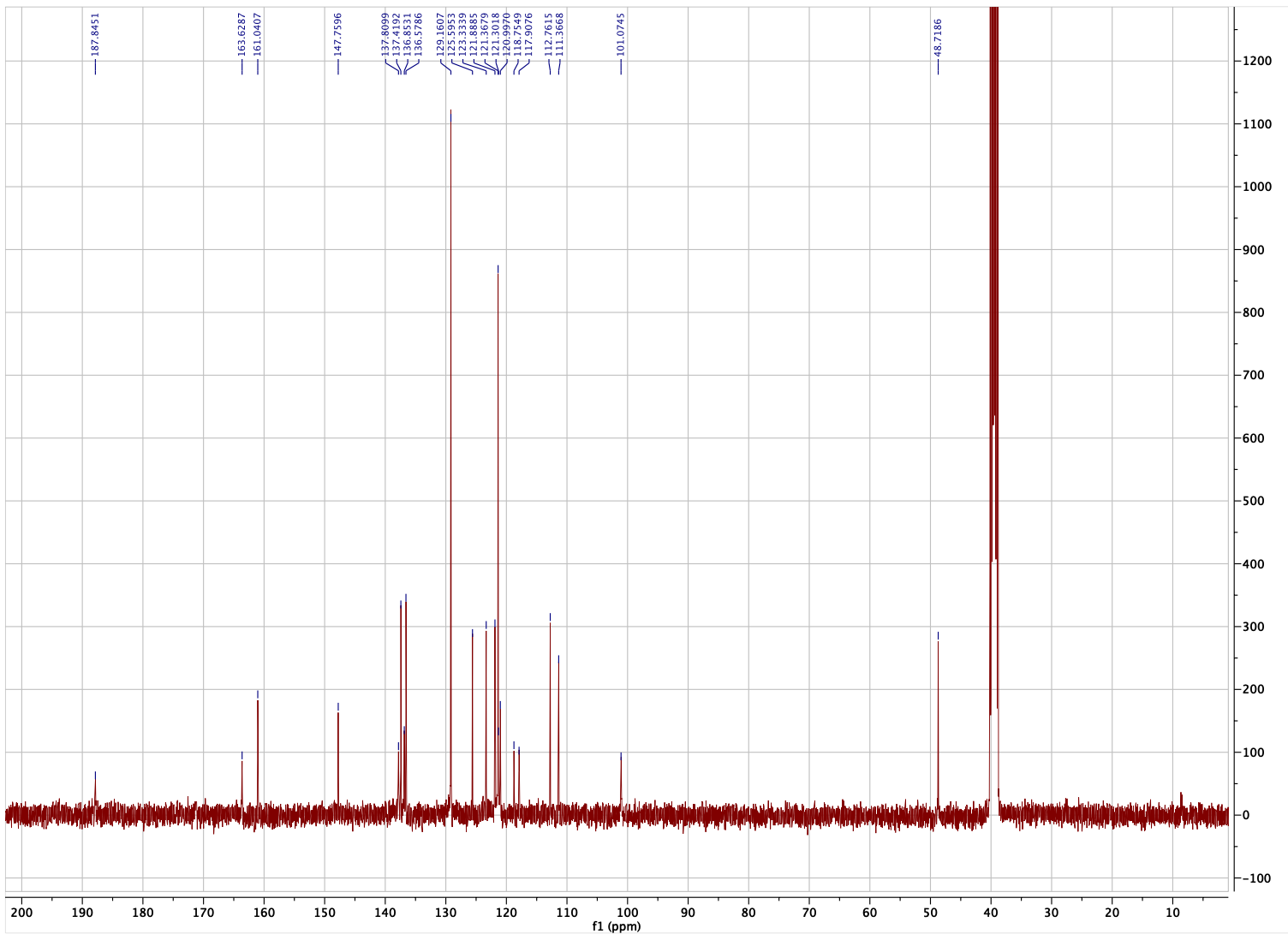


Figure S14. ^13^C NMR Spectrum for compound **5e**


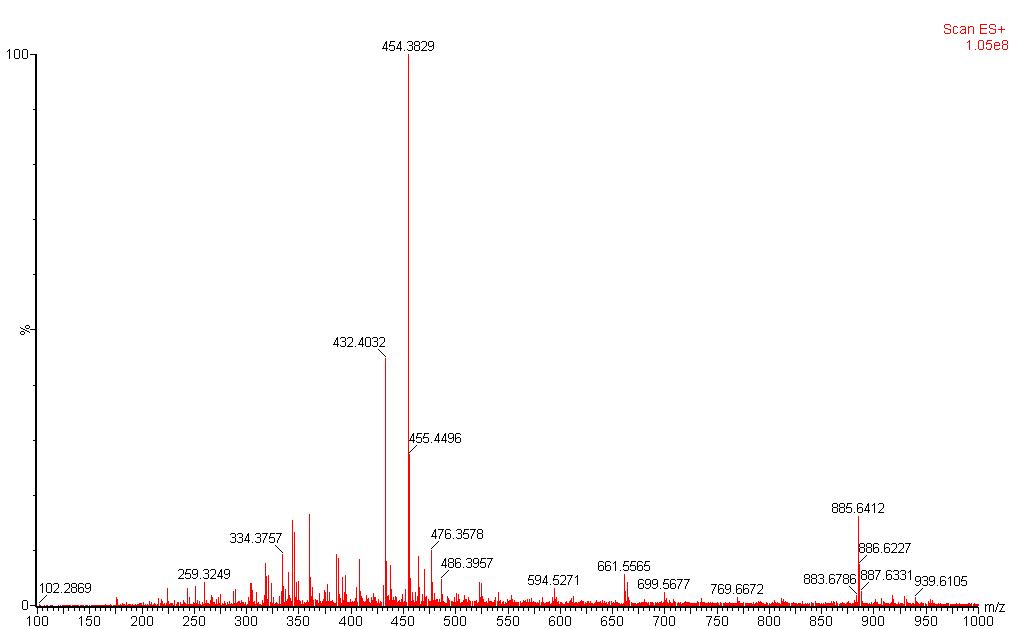


Figure S15. MS (ESI) Spectrum for compound **5e**


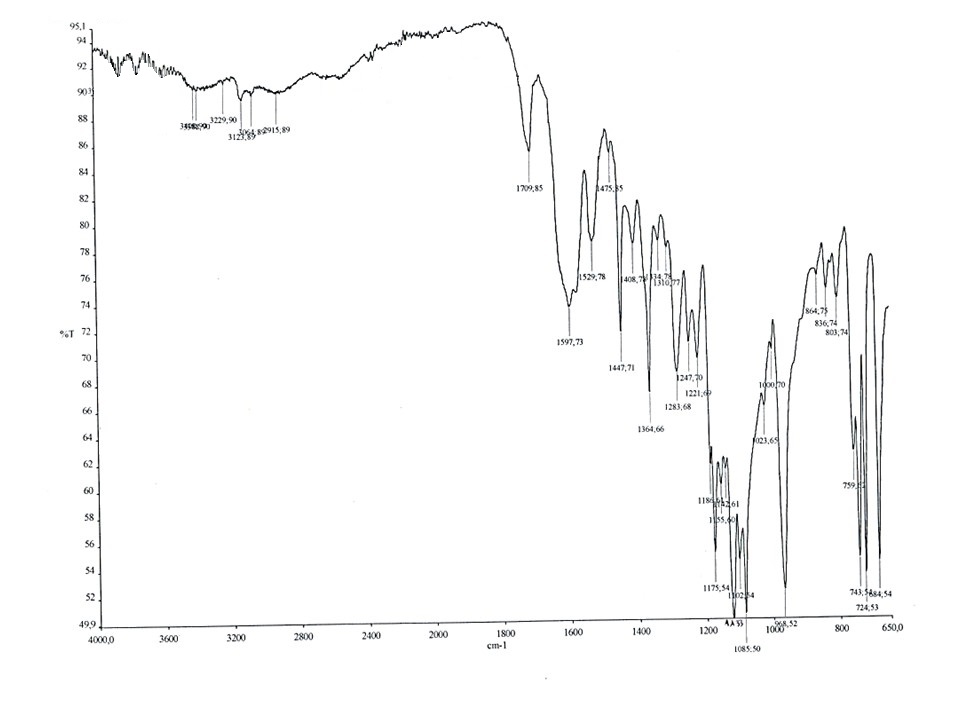
Figure S16. FTIR Spectrum for compound **5f**


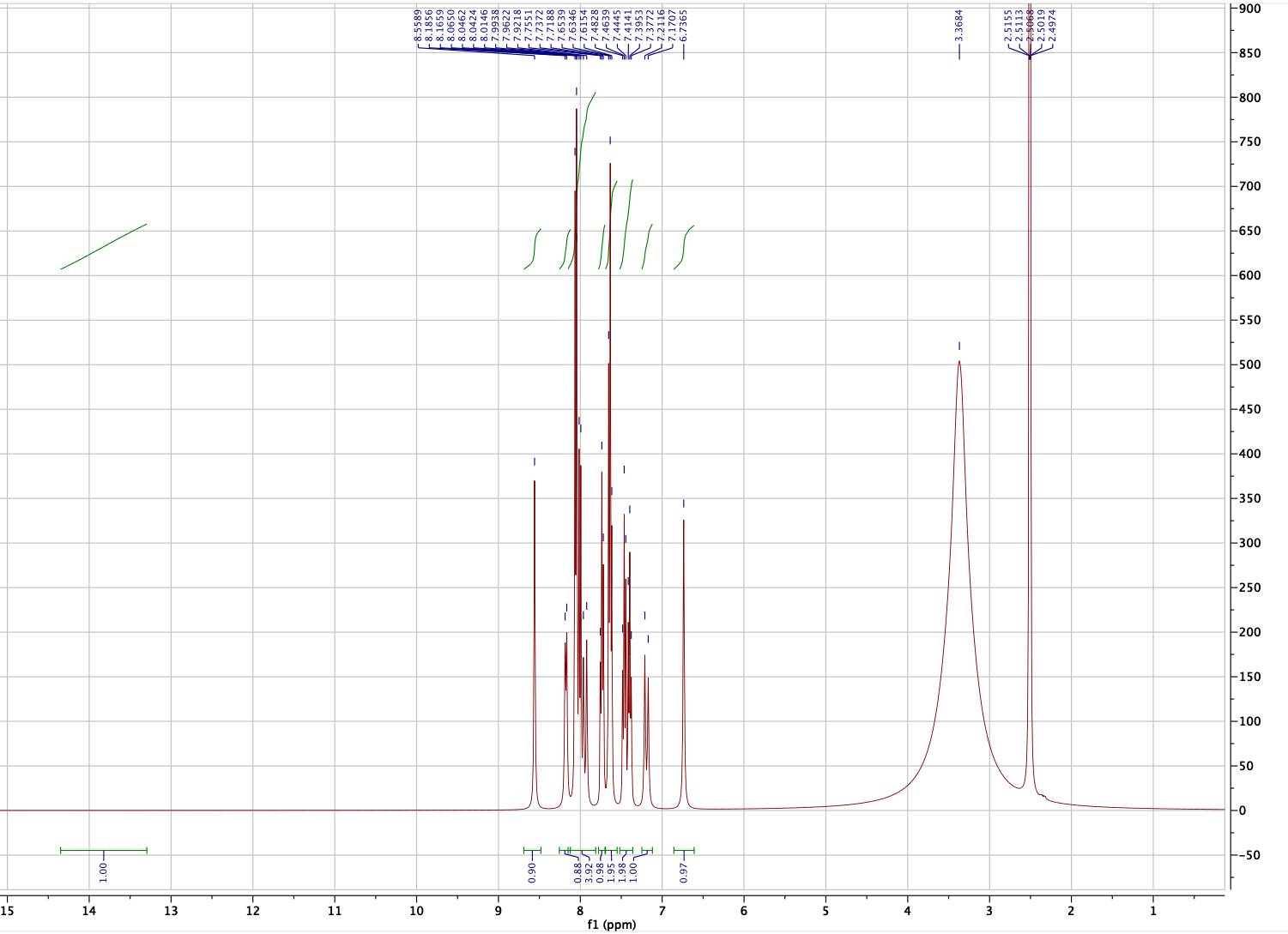


Figure S17. ^1^H NMR Spectrum for compound **5f**


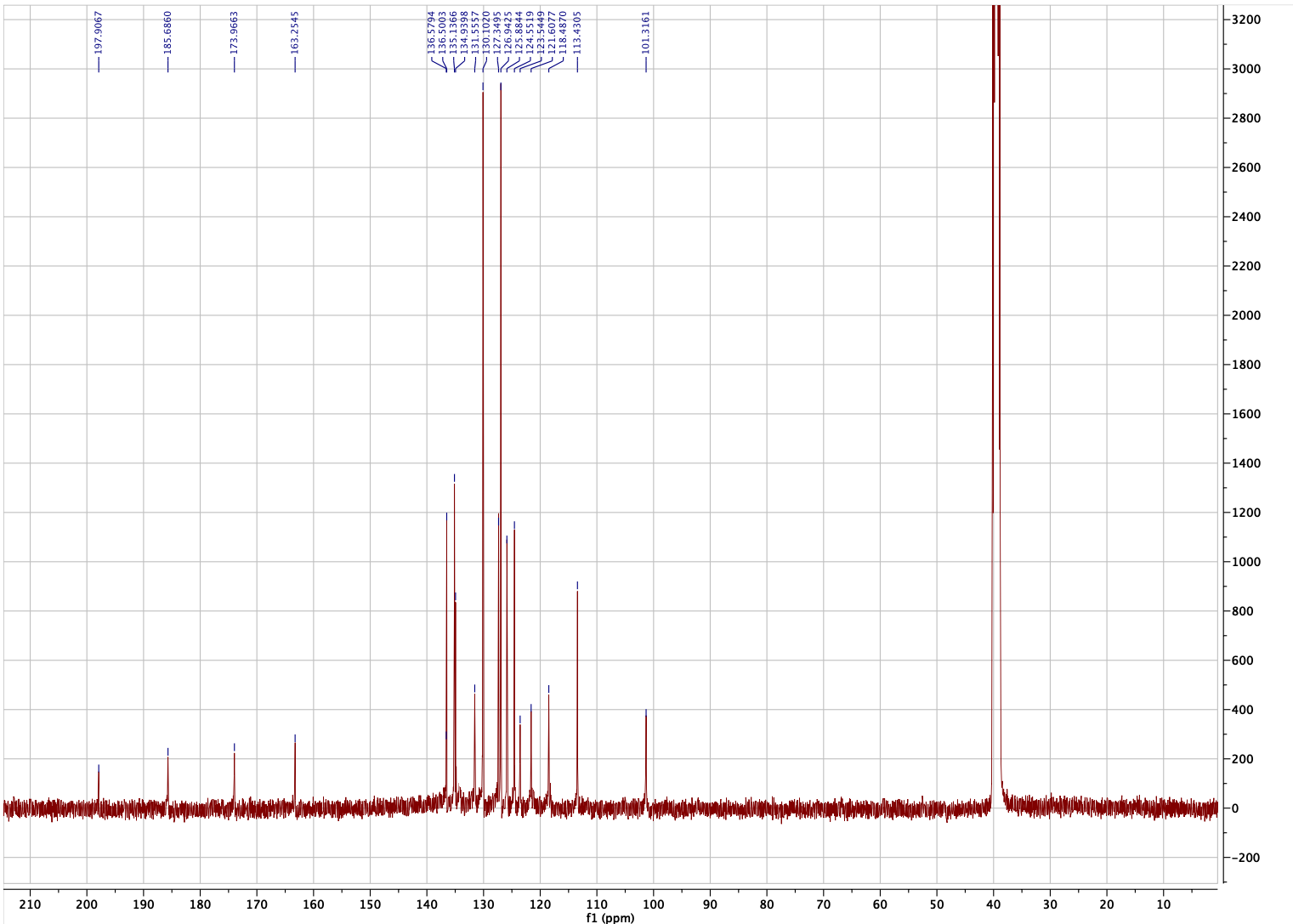


Figure S18. ^13^C NMR Spectrum for compound **5f**


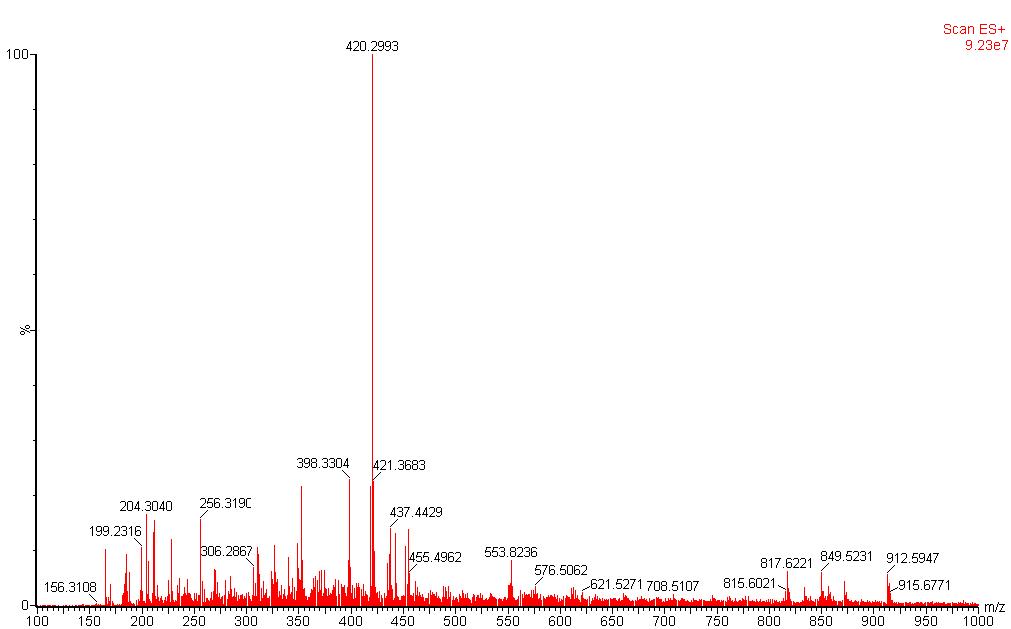


Figure S19. MS (ESI) Spectrum for compound **5f**


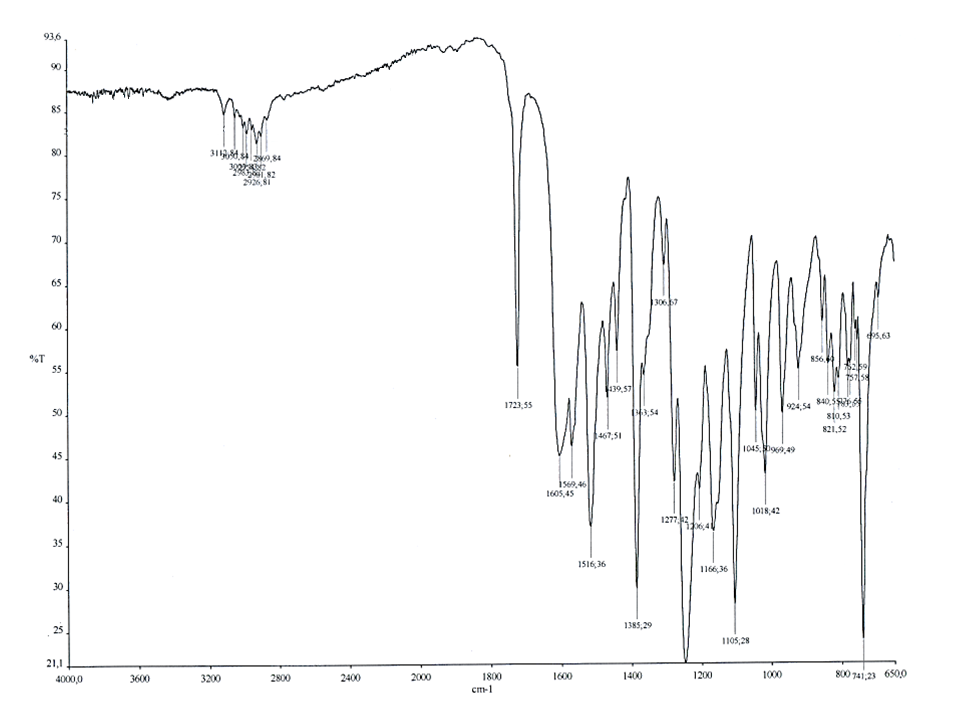


Figure S20. FTIR Spectrum for compound **6b**


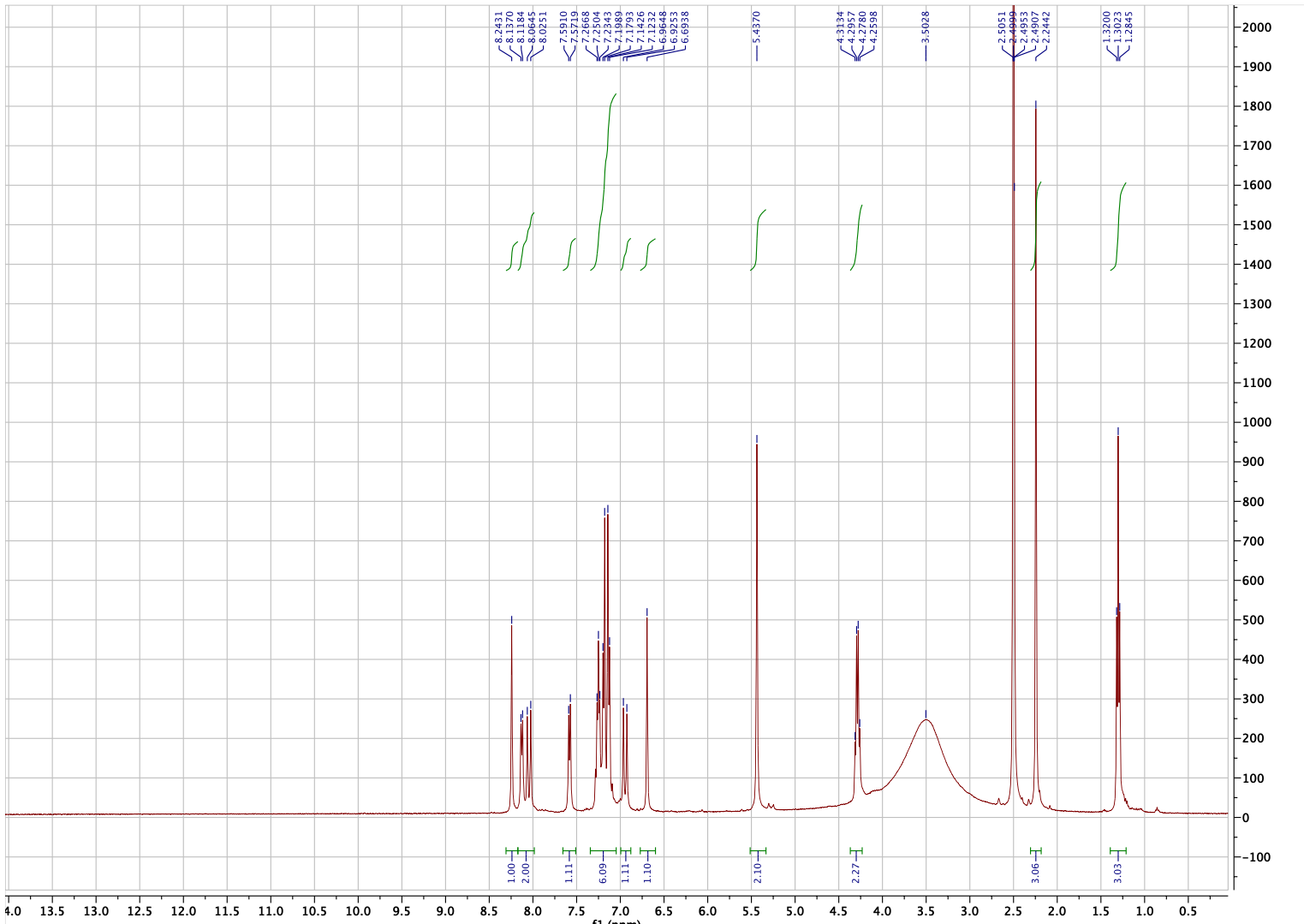


Figure S21. ^1^H NMR Spectrum for compound **6b**


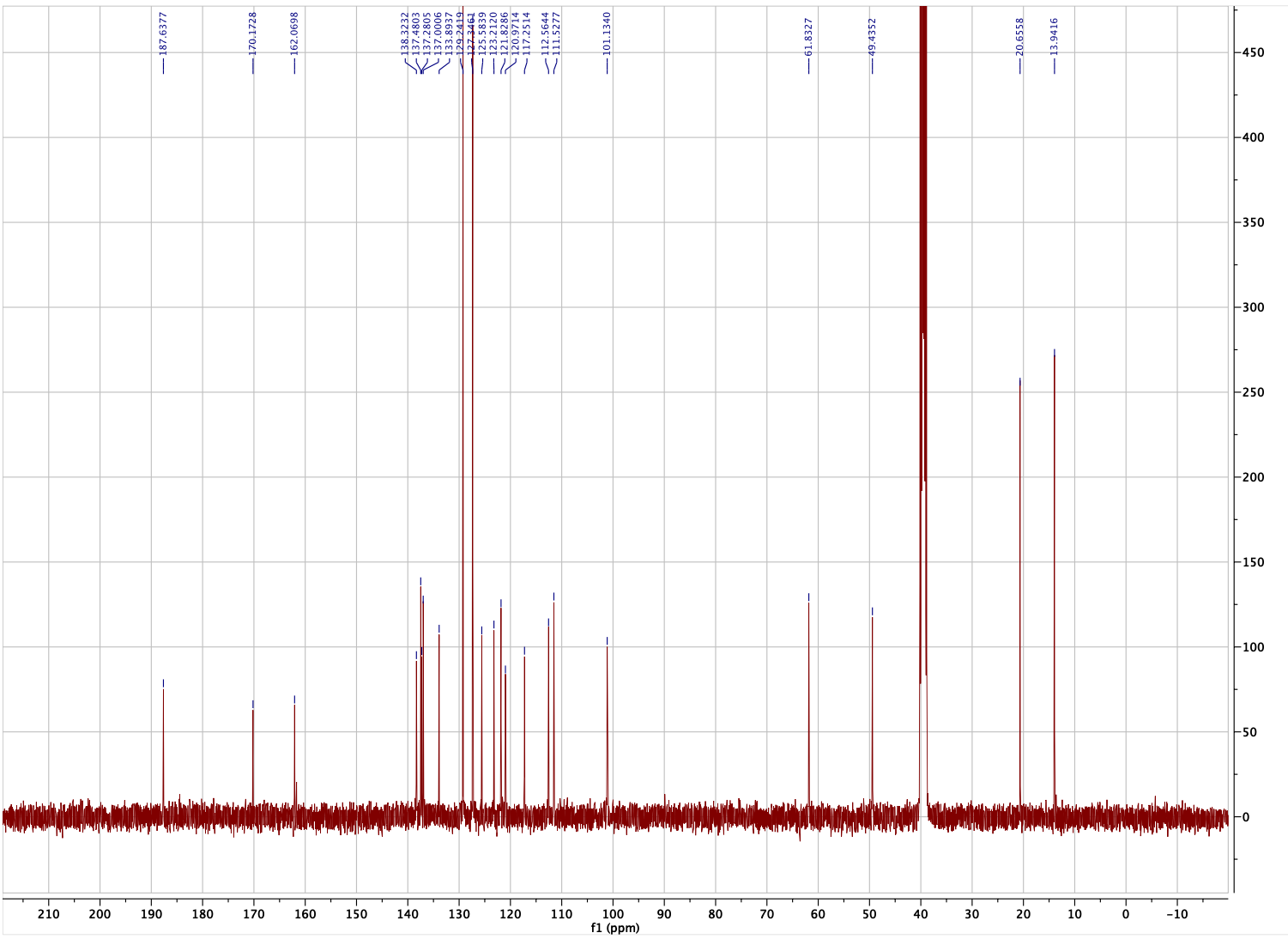


Figure S22. ^13^C NMR Spectrum for compound **6b**


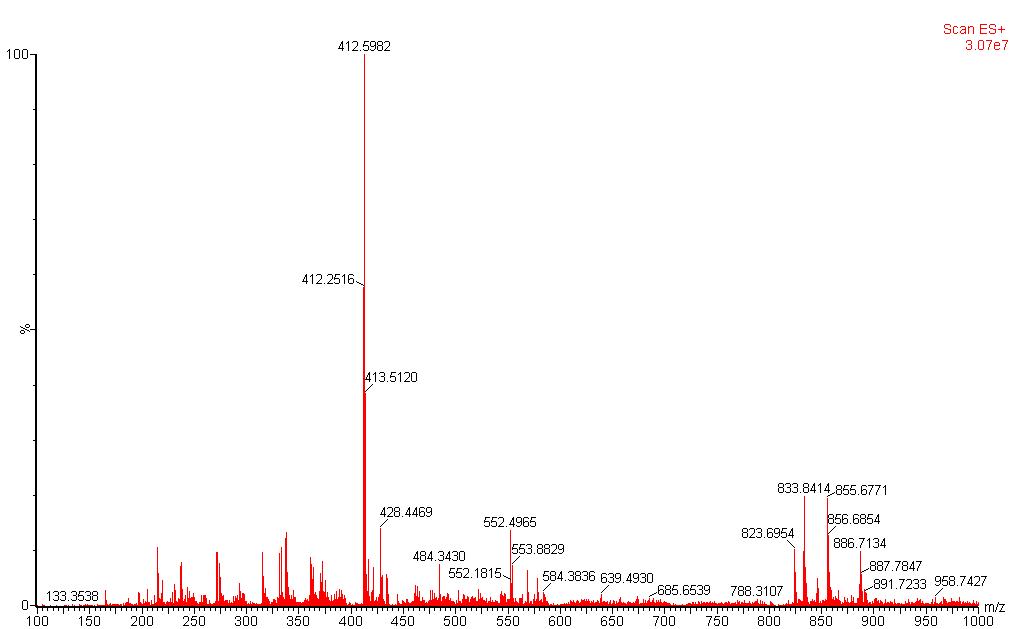


Figure S23. MS (ESI) Spectrum for compound **6b**


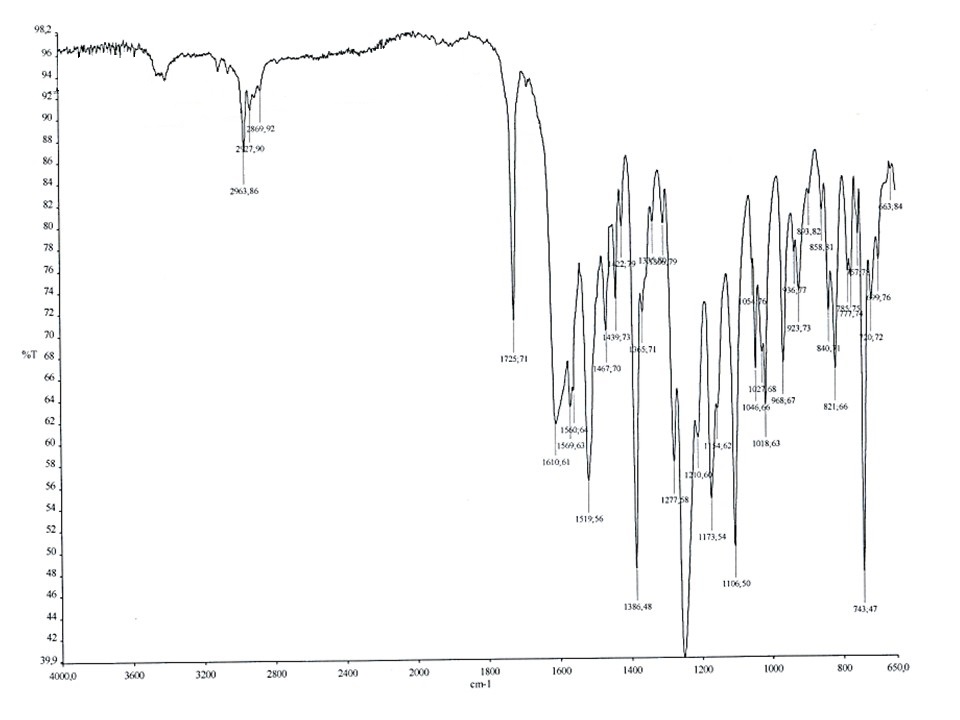


Figure S24. FTIR Spectrum for compound **6c**


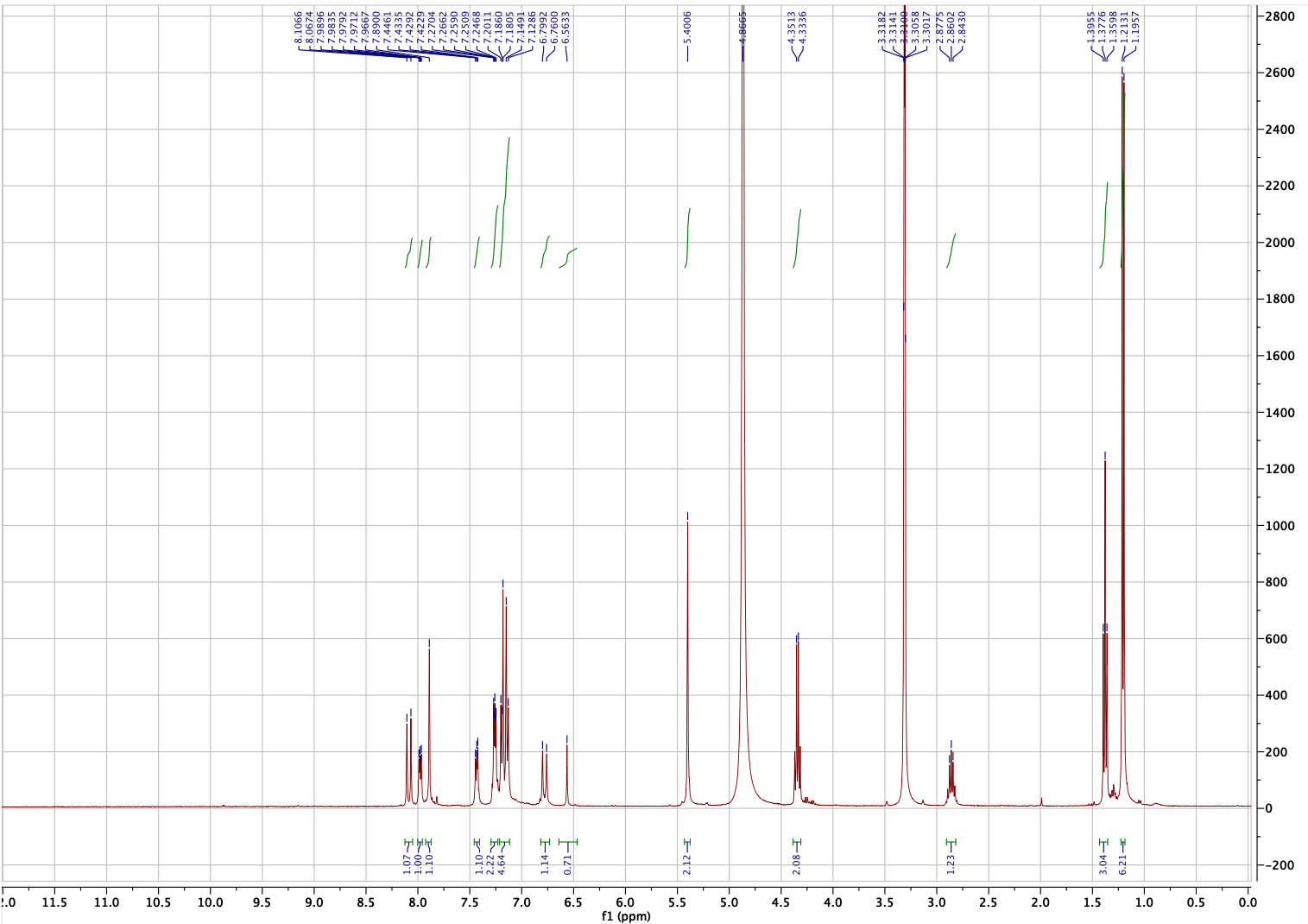


Figure S25. ^1^H NMR Spectrum for compound **6c**


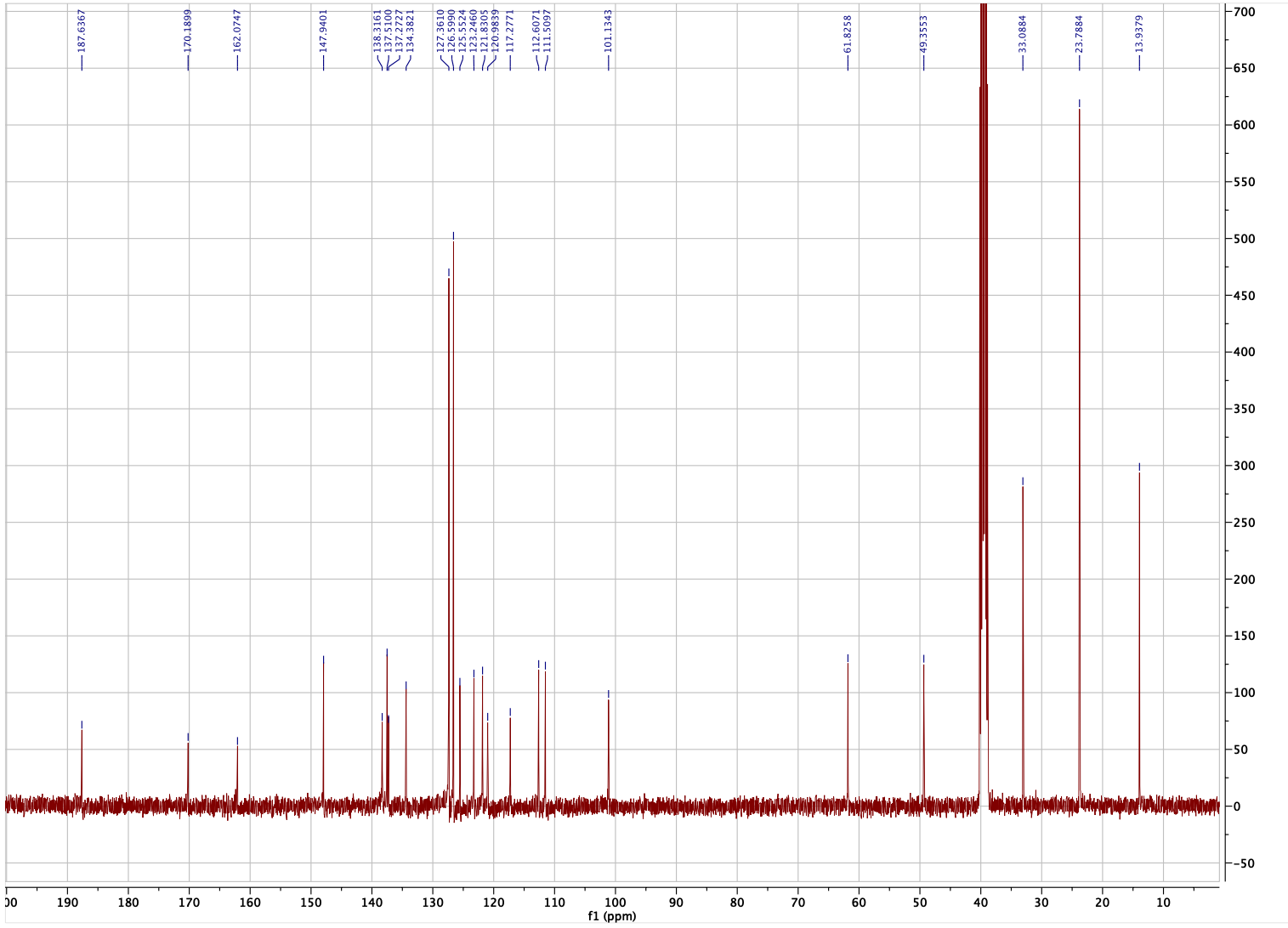
Figure S26. ^13^C NMR Spectrum for compound **6c**


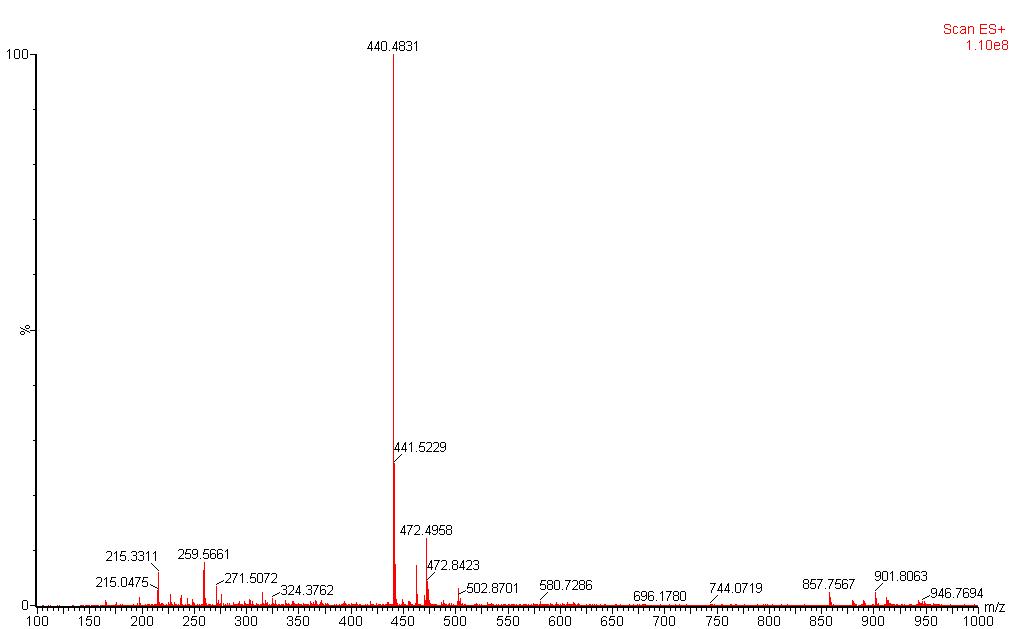


Figure S27. MS (ESI) Spectrum for compound **6c**


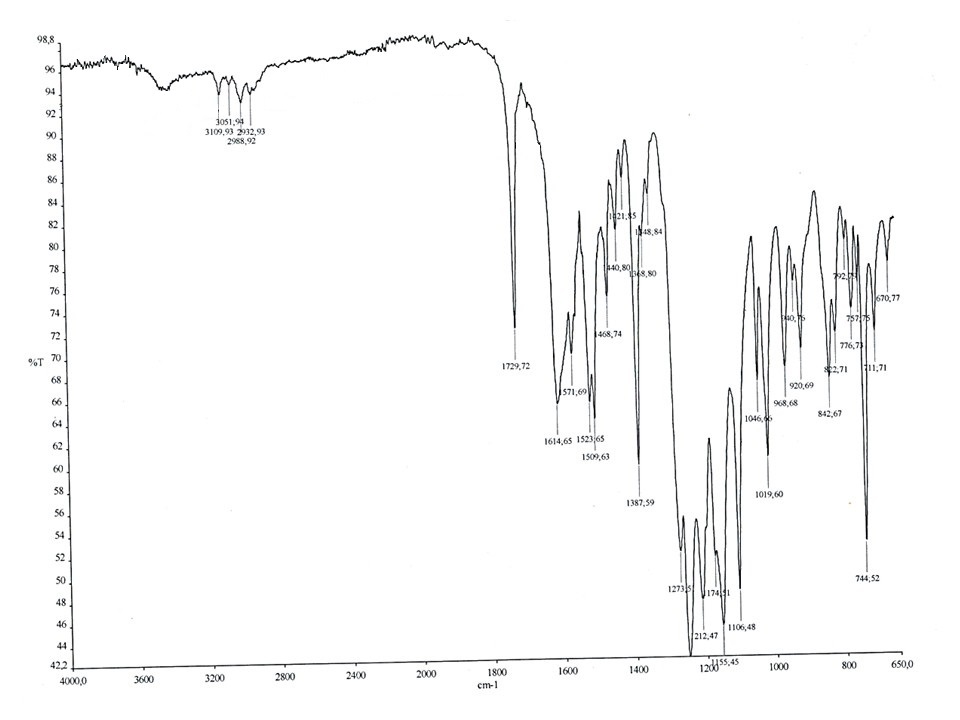


Figure S28. FTIR Spectrum for compound **6e**


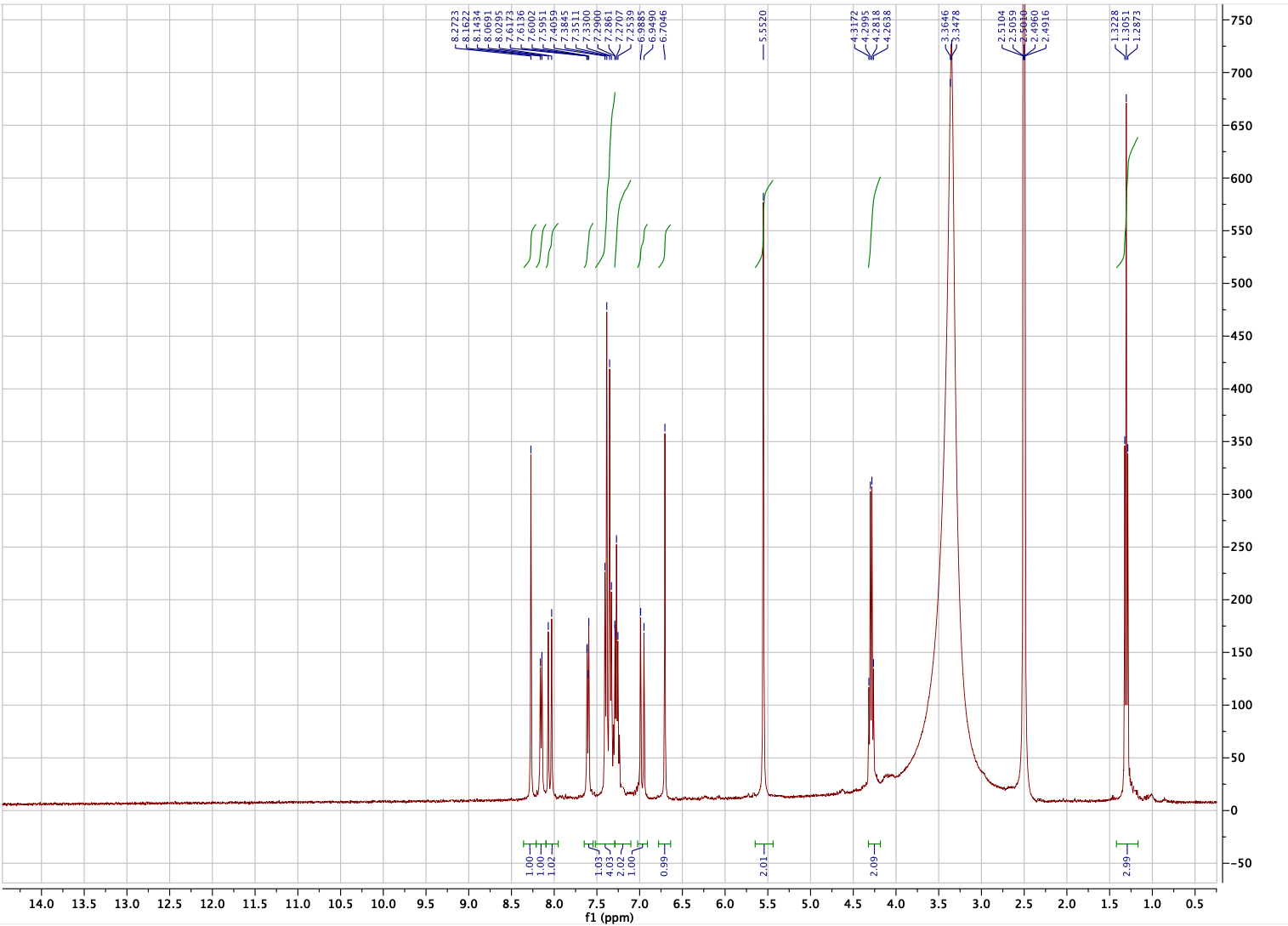


Figure S29. ^1^H NMR Spectrum for compound **6e**


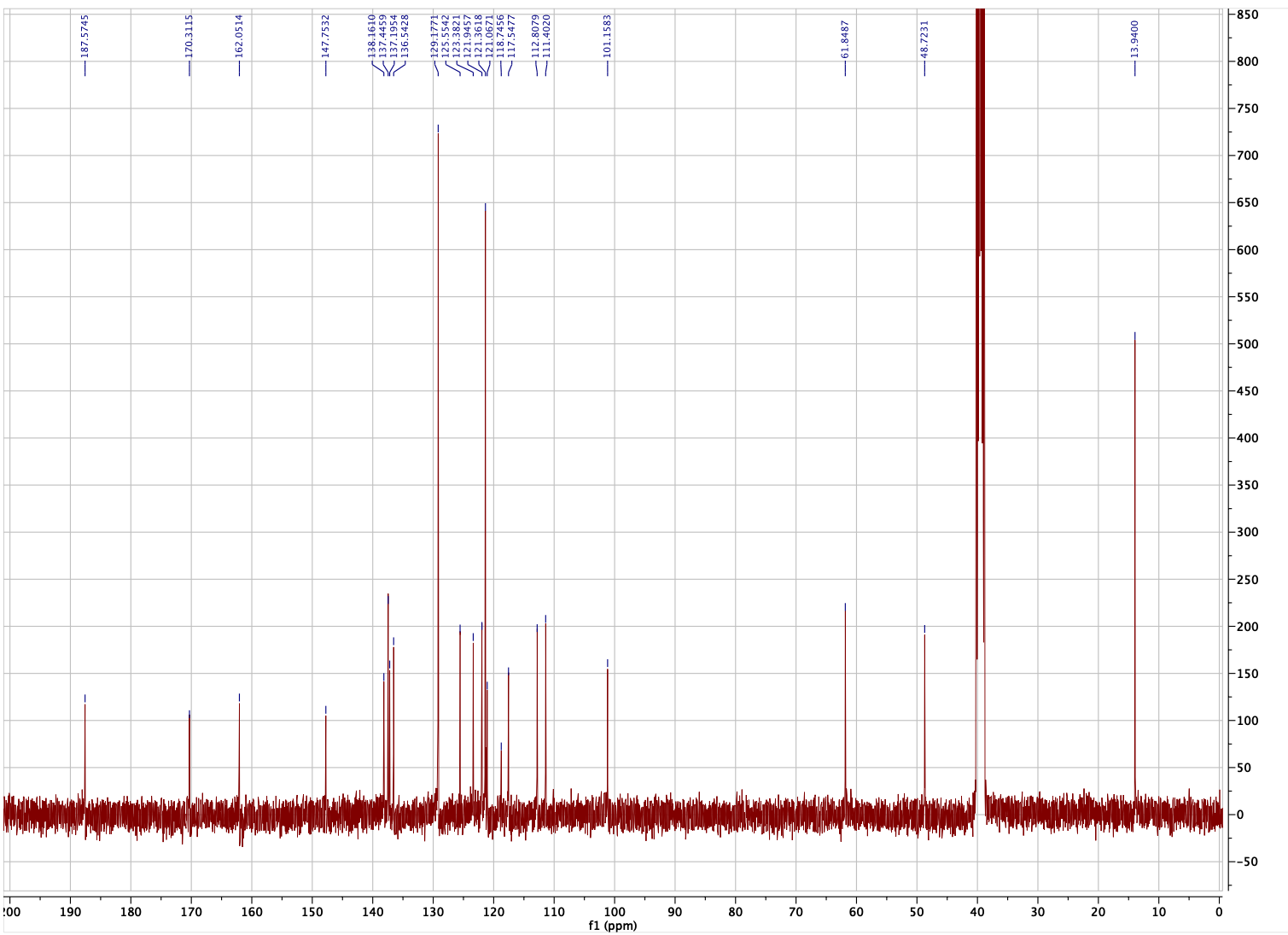


Figure S30. ^13^C NMR Spectrum for compound **6e**


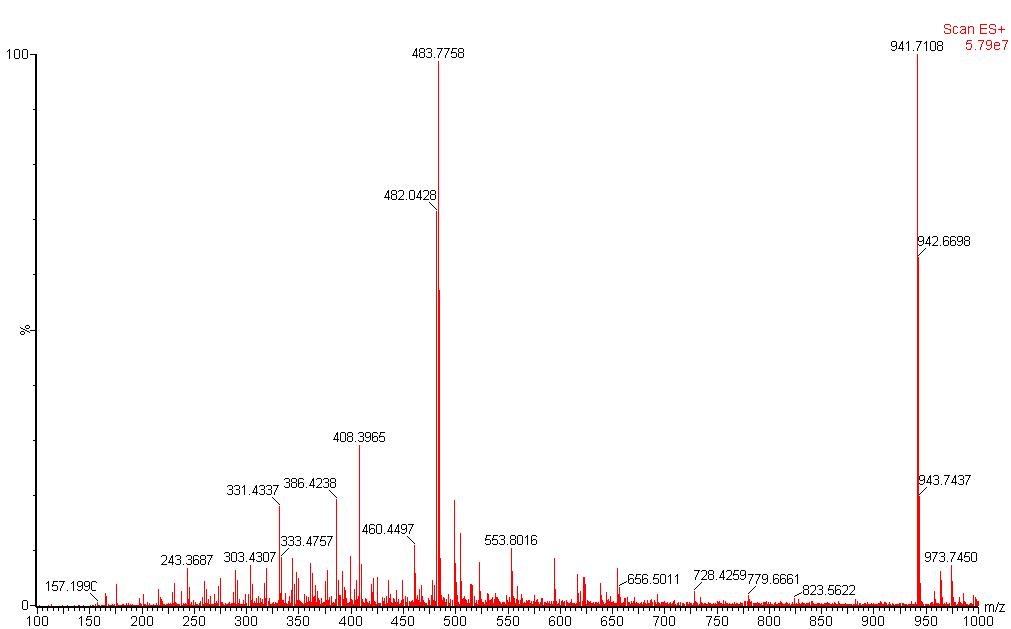


Figure S31. MS (ESI) Spectrum for compound **6e**


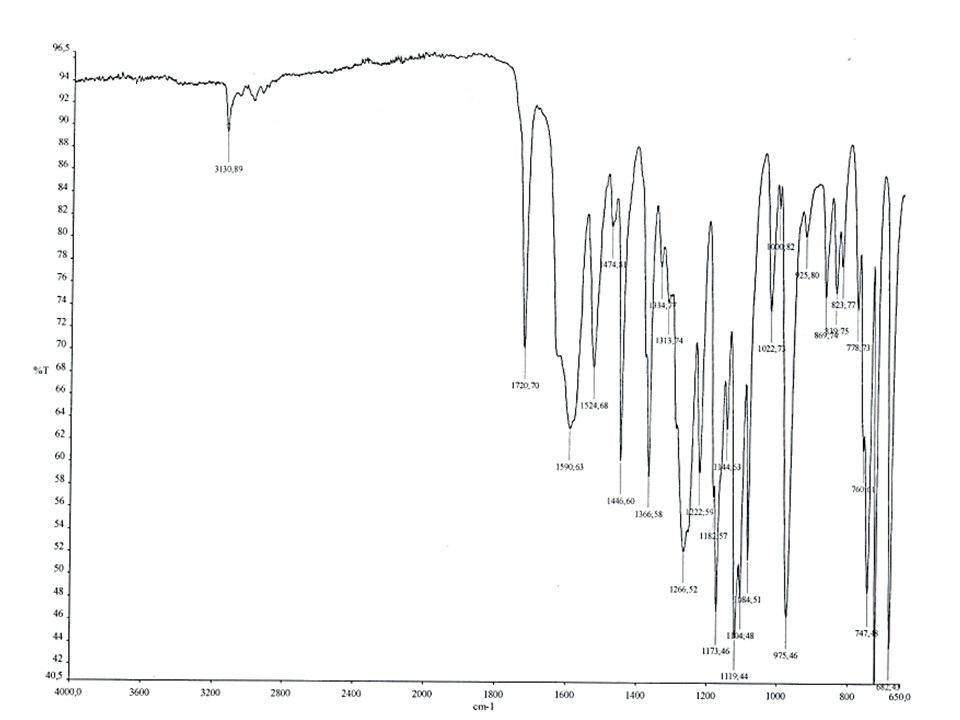


Figure S32. FTIR Spectrum for compound **6f**


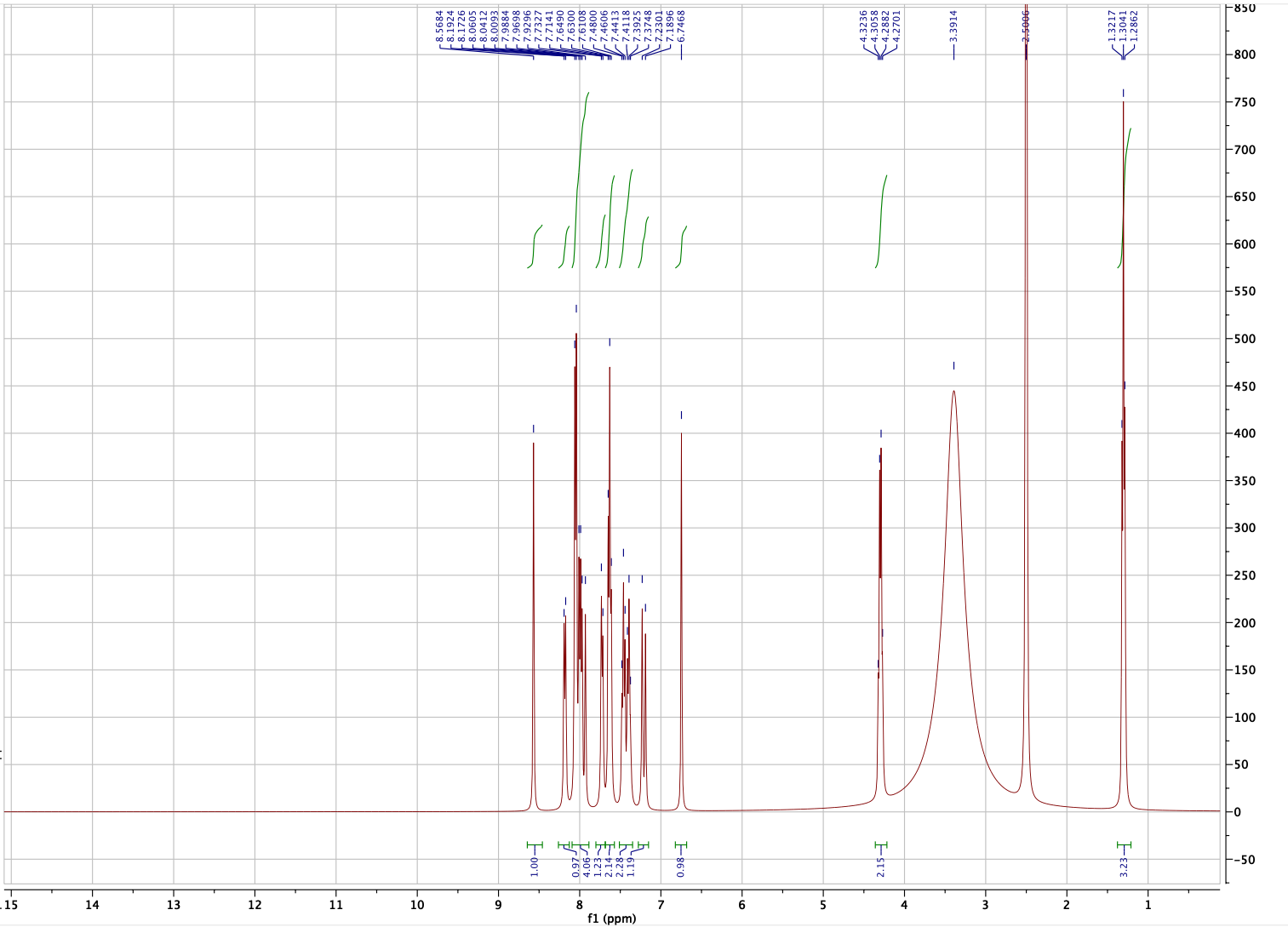


Figure S33. ^1^H NMR Spectrum for compound **6f**


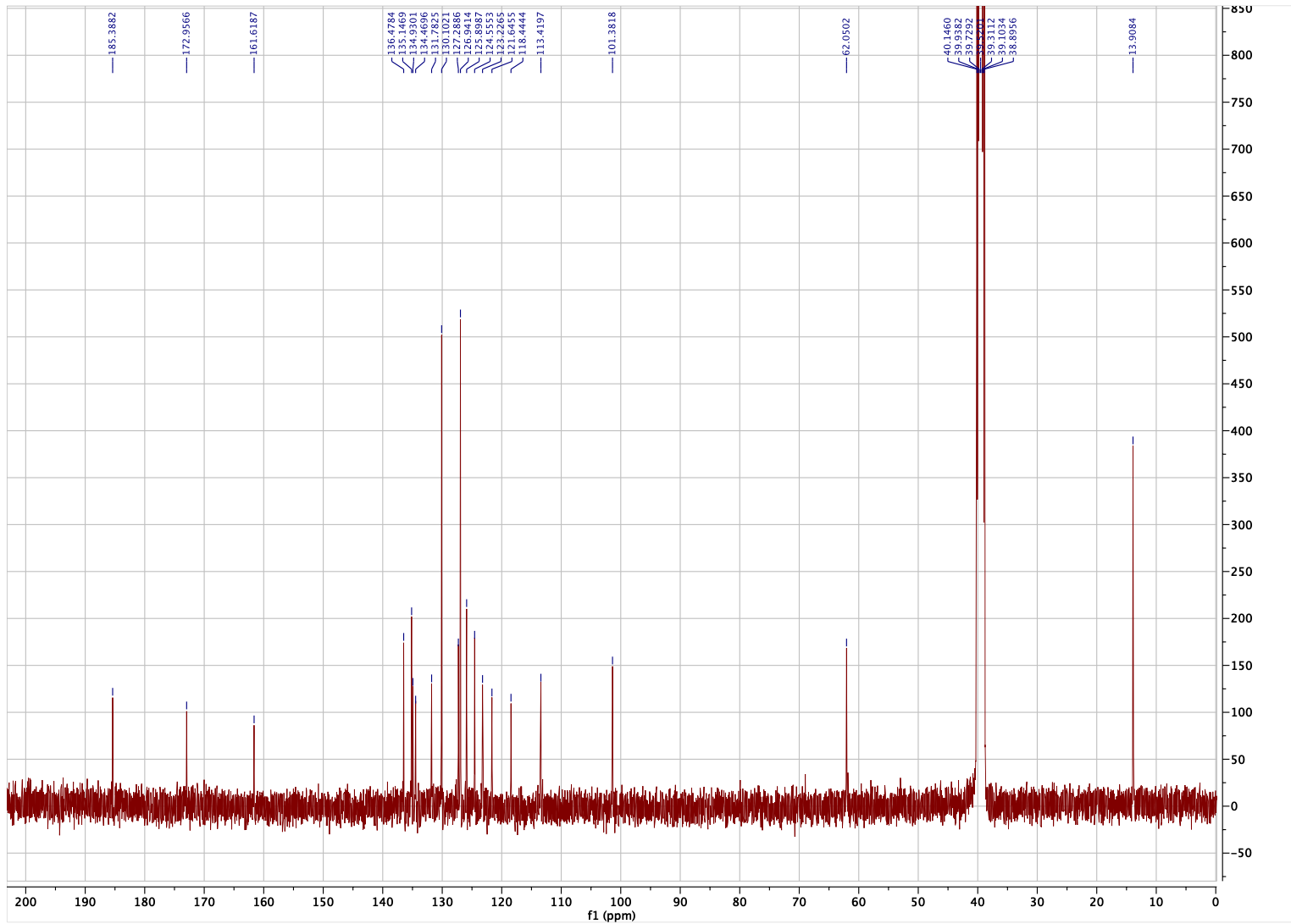
Figure S34. ^13^C NMR Spectrum for compound **6f**


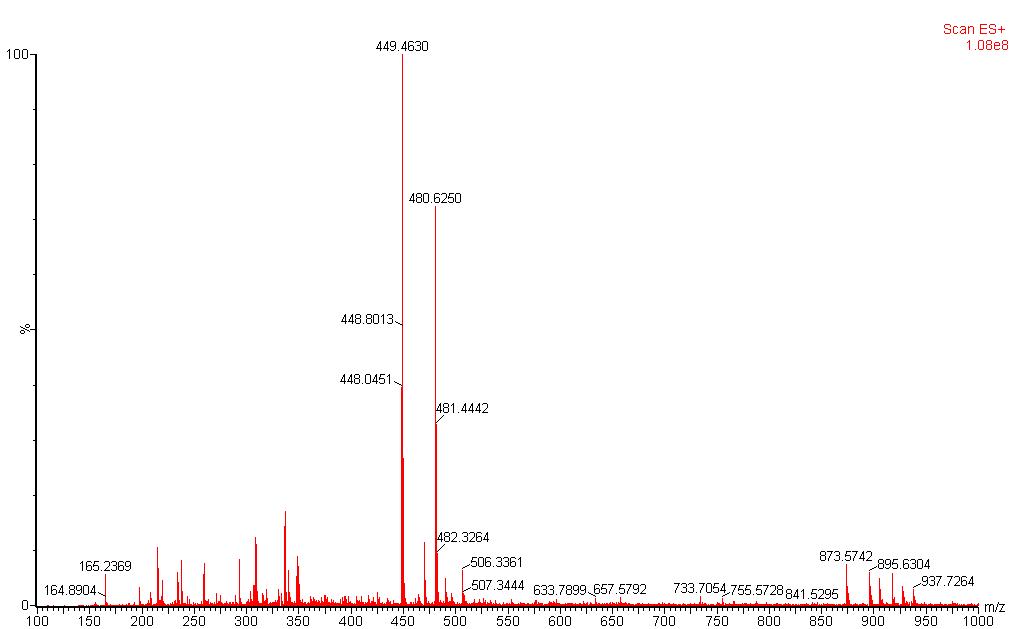


Figure S35. MS (ESI) Spectrum for compound **6f**


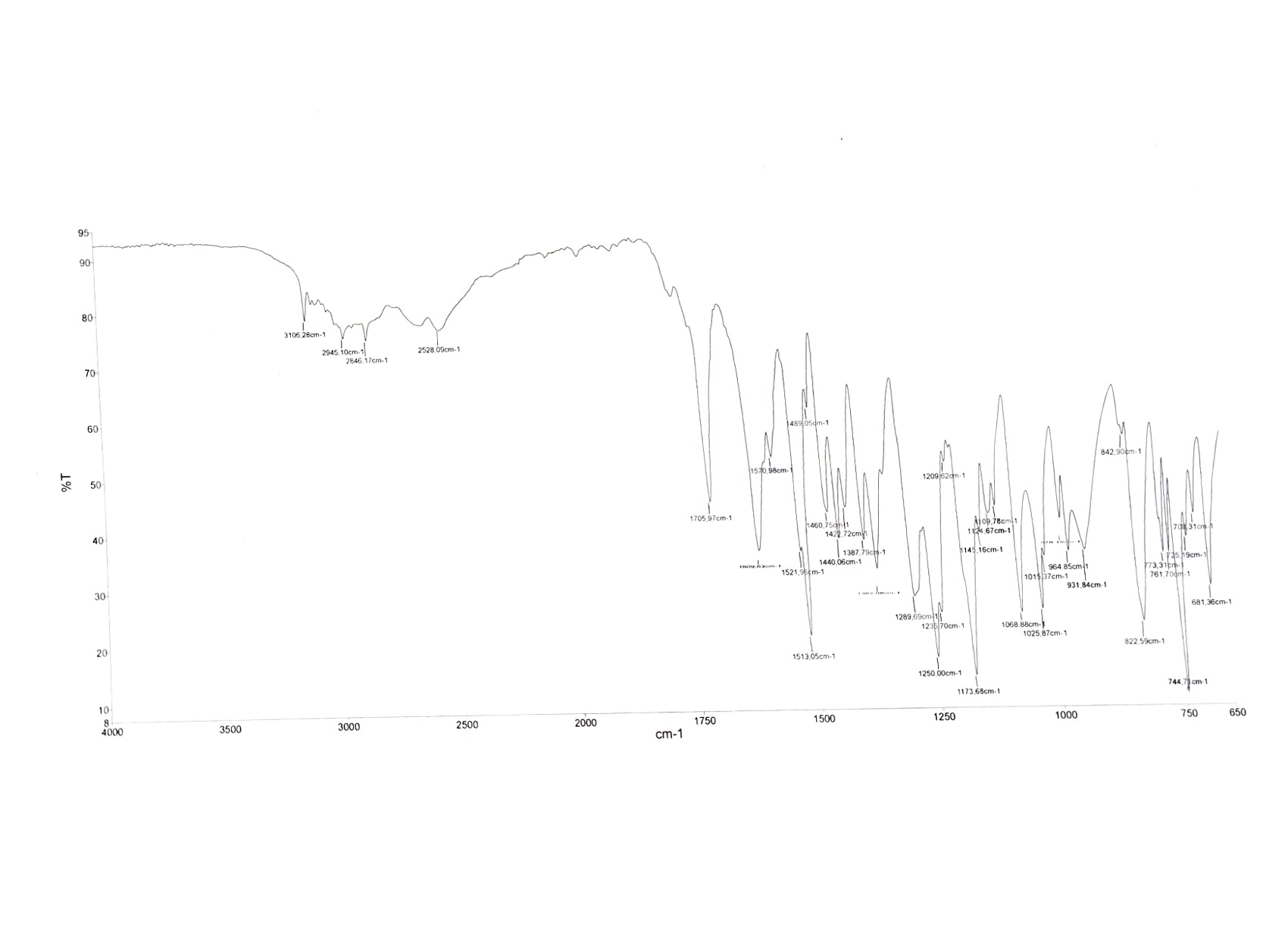


Figure S36. FTIR Spectrum for compound **7a**


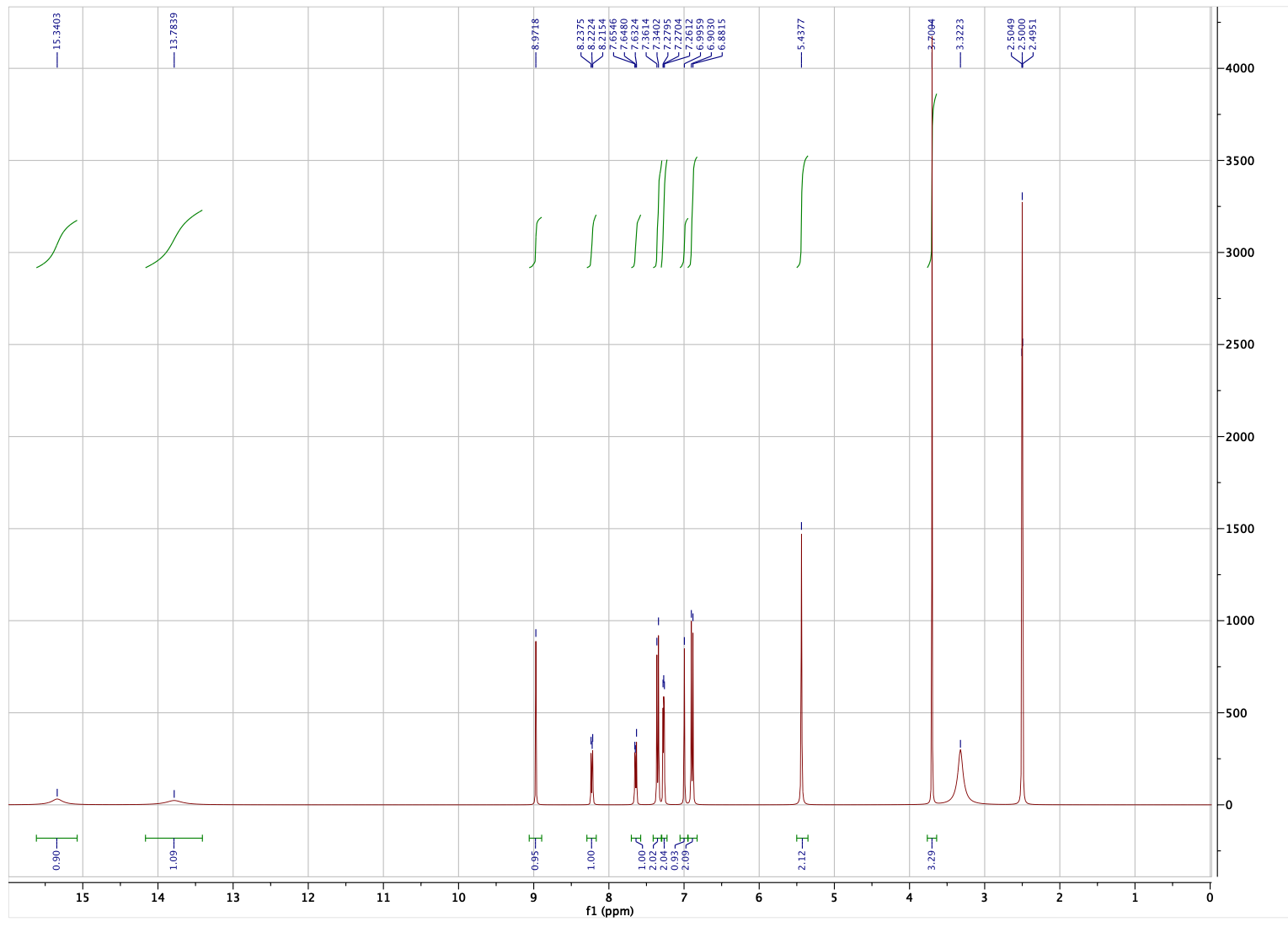
Figure S37. ^1^H NMR Spectrum for compound **7a**


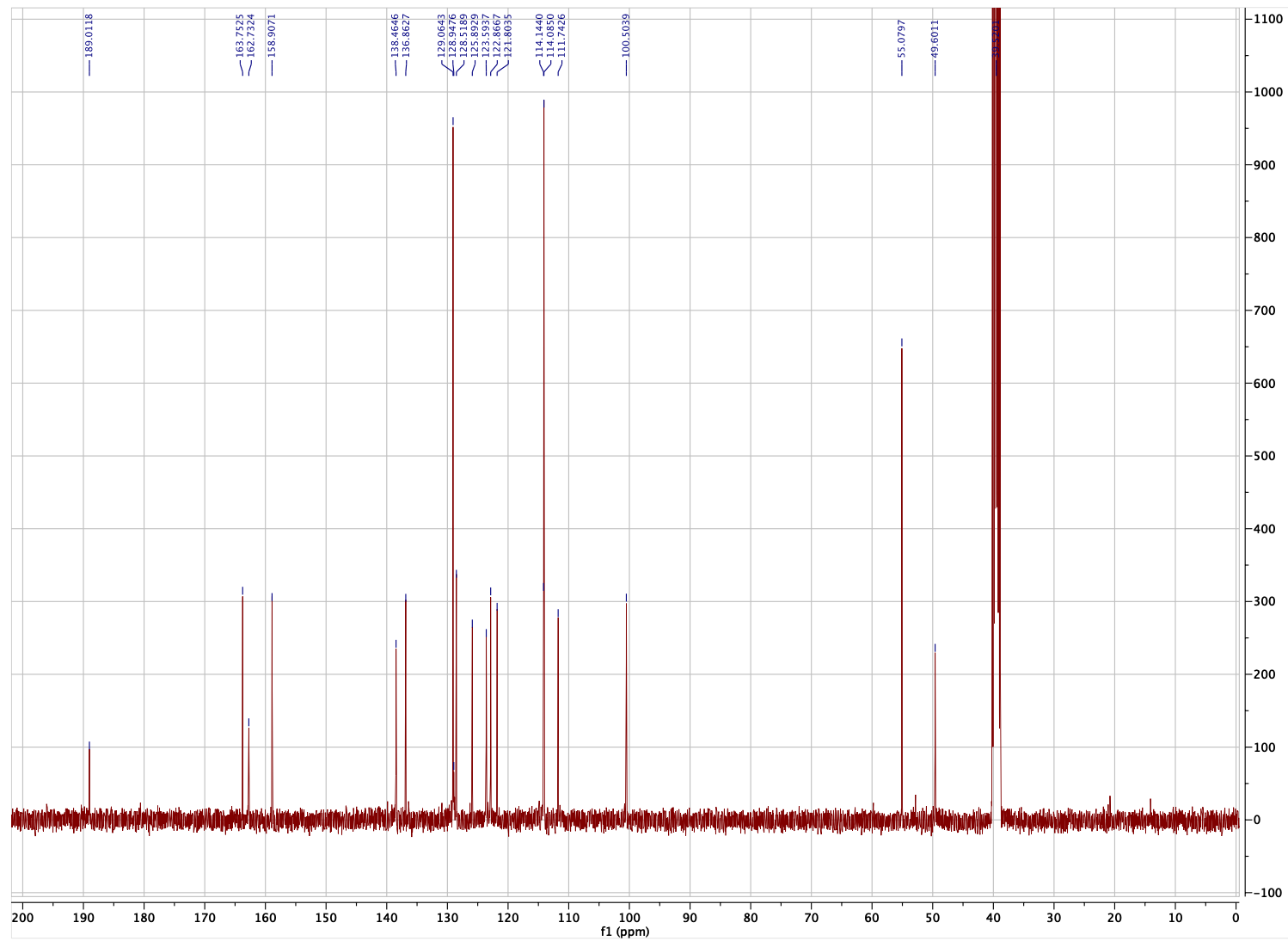


Figure S38. ^13^C NMR Spectrum for compound **7a**


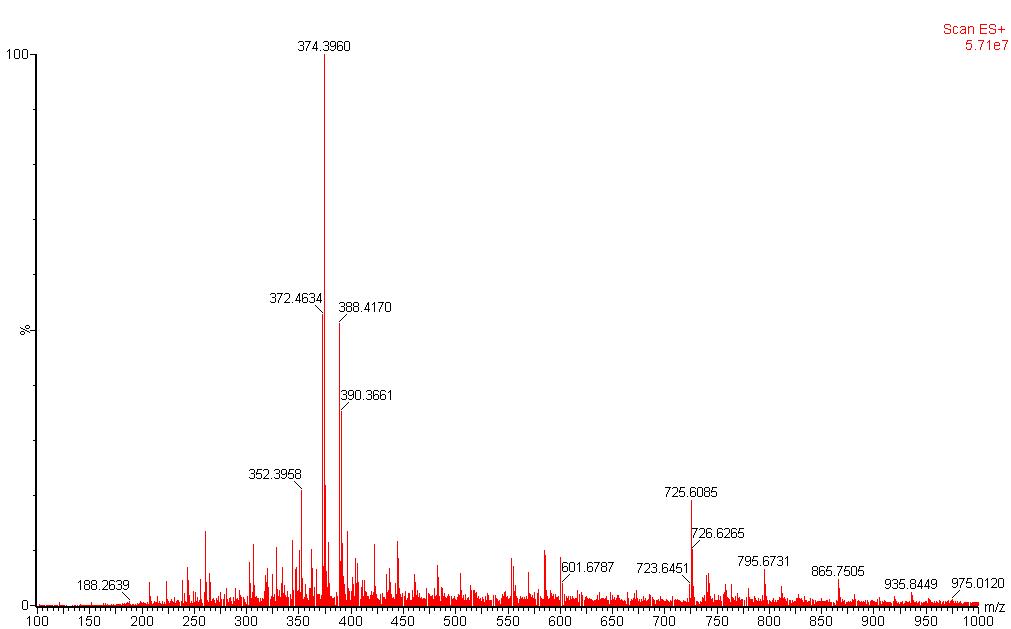


Figure S39. MS (ESI) Spectrum for compound **7a**


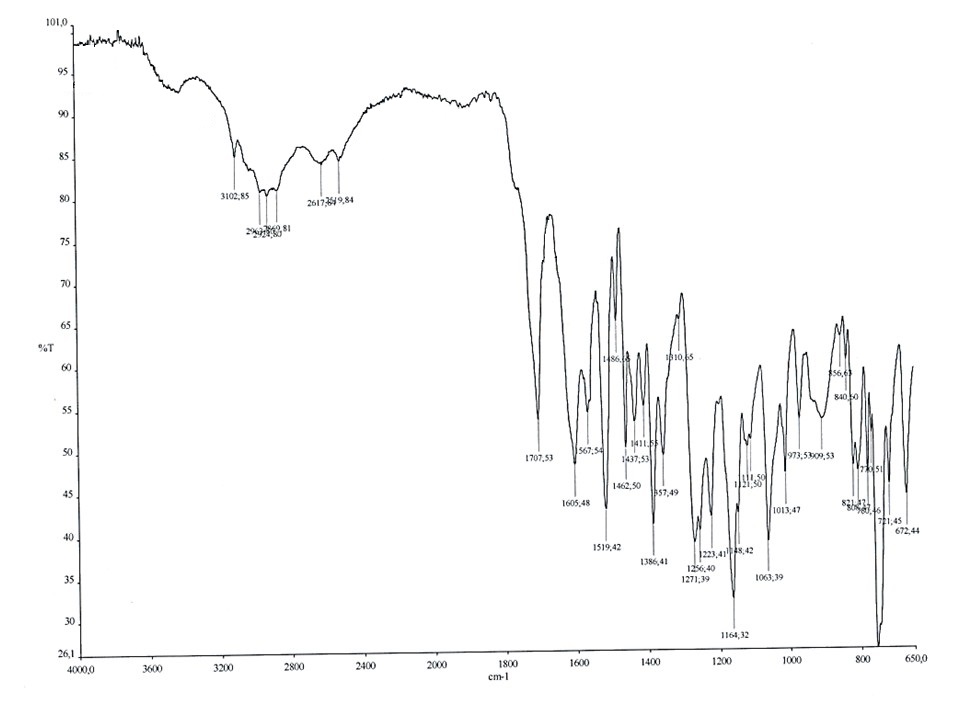


Figure S40. FTIR Spectrum for compound **7b**


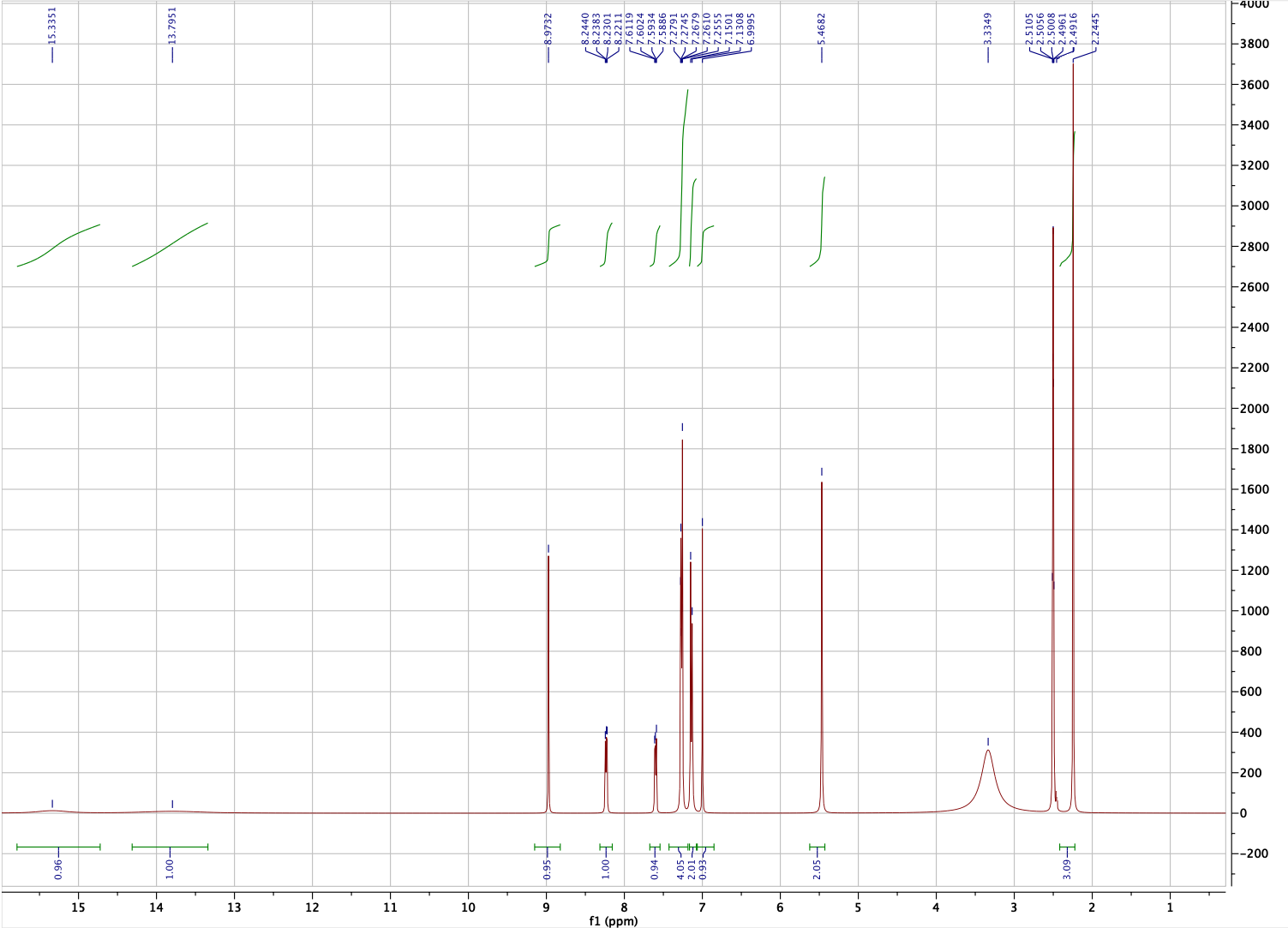


Figure S41. ^1^H NMR Spectrum for compound **7b**


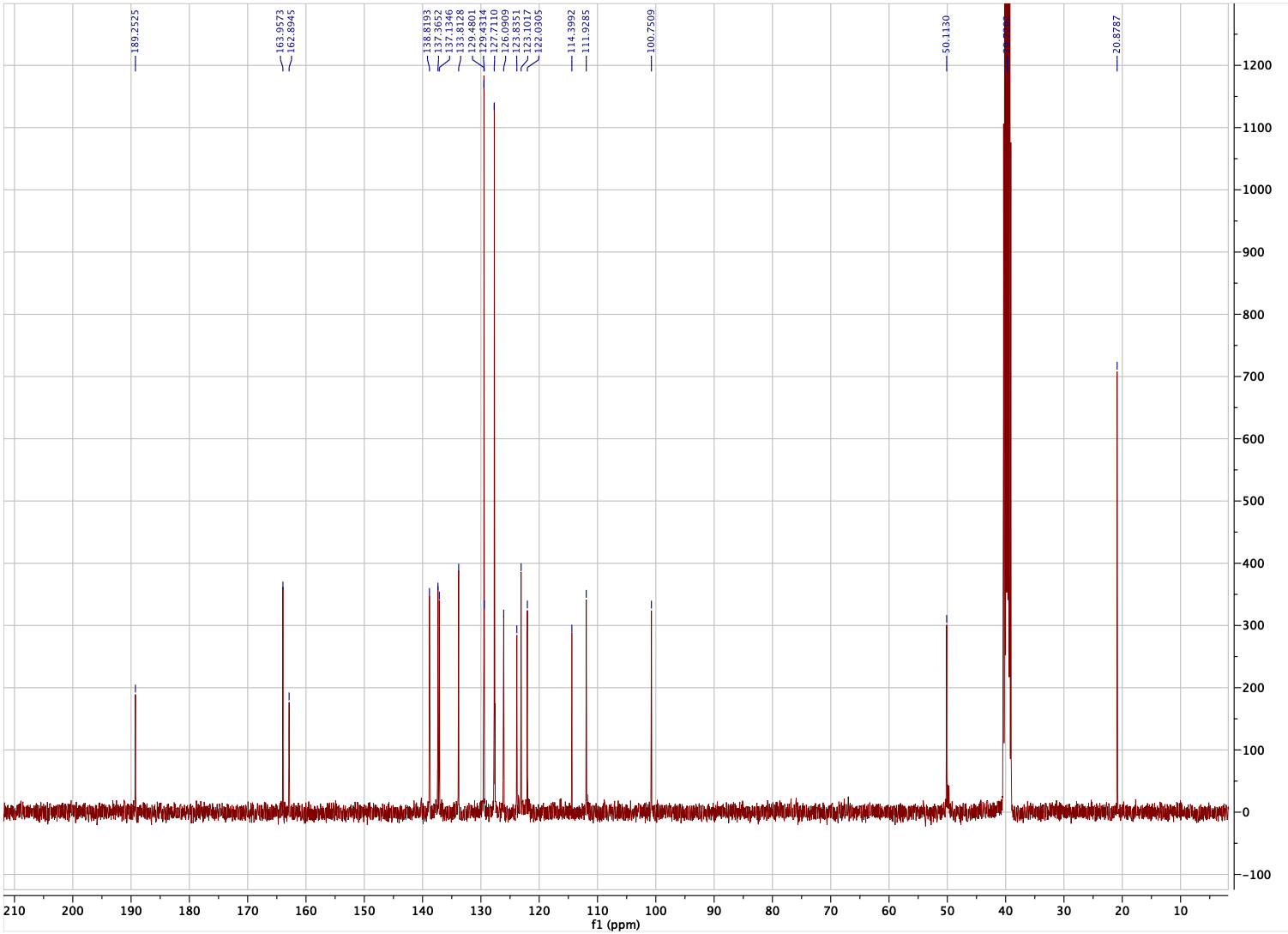


Figure S42. ^13^C NMR Spectrum for compound **7b**

**
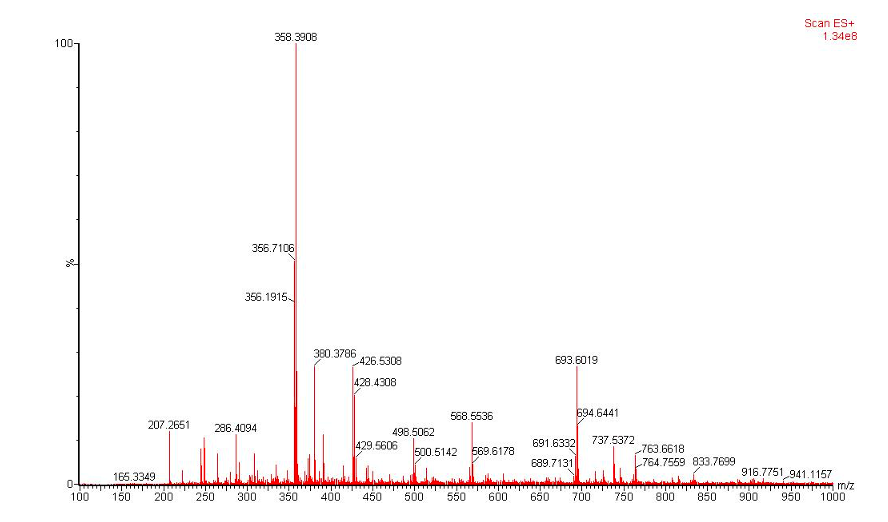
**

Figure S43. MS (ESI) Spectrum for compound **7b**


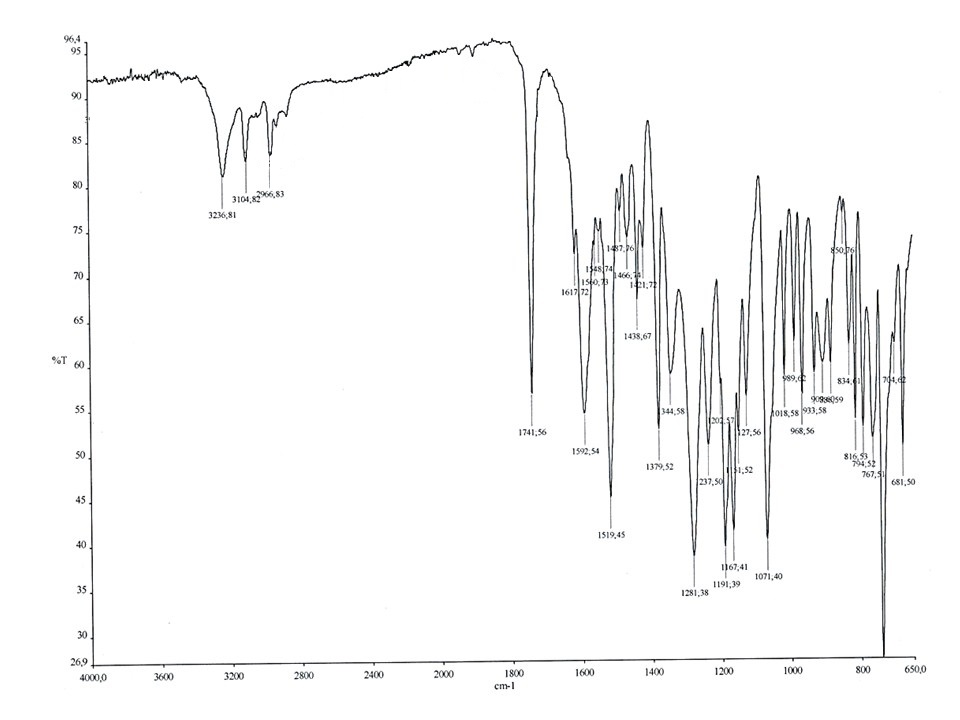


Figure S44. FTIR Spectrum for compound **7c**


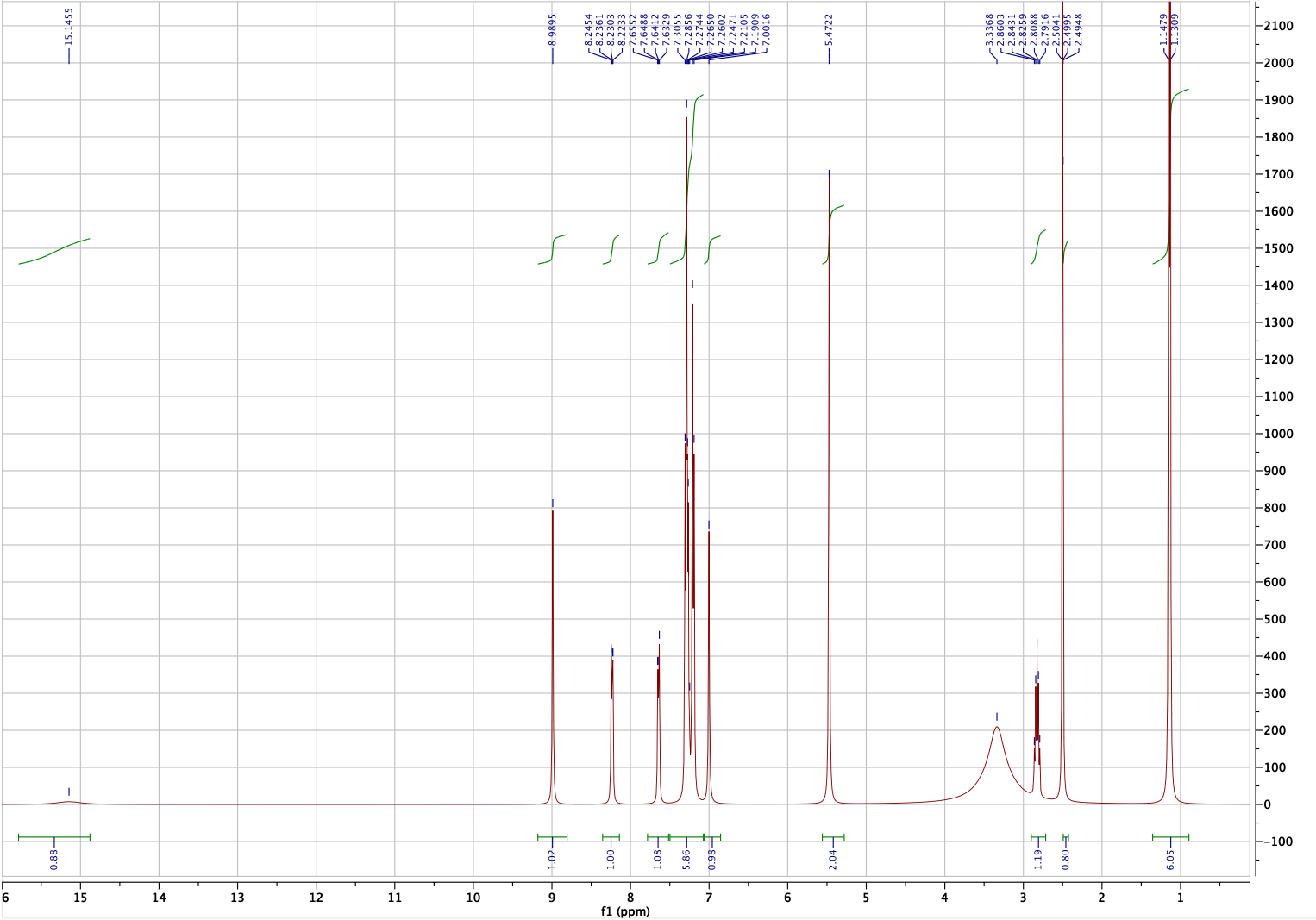


Figure S45. ^1^H NMR Spectrum for compound **7c**


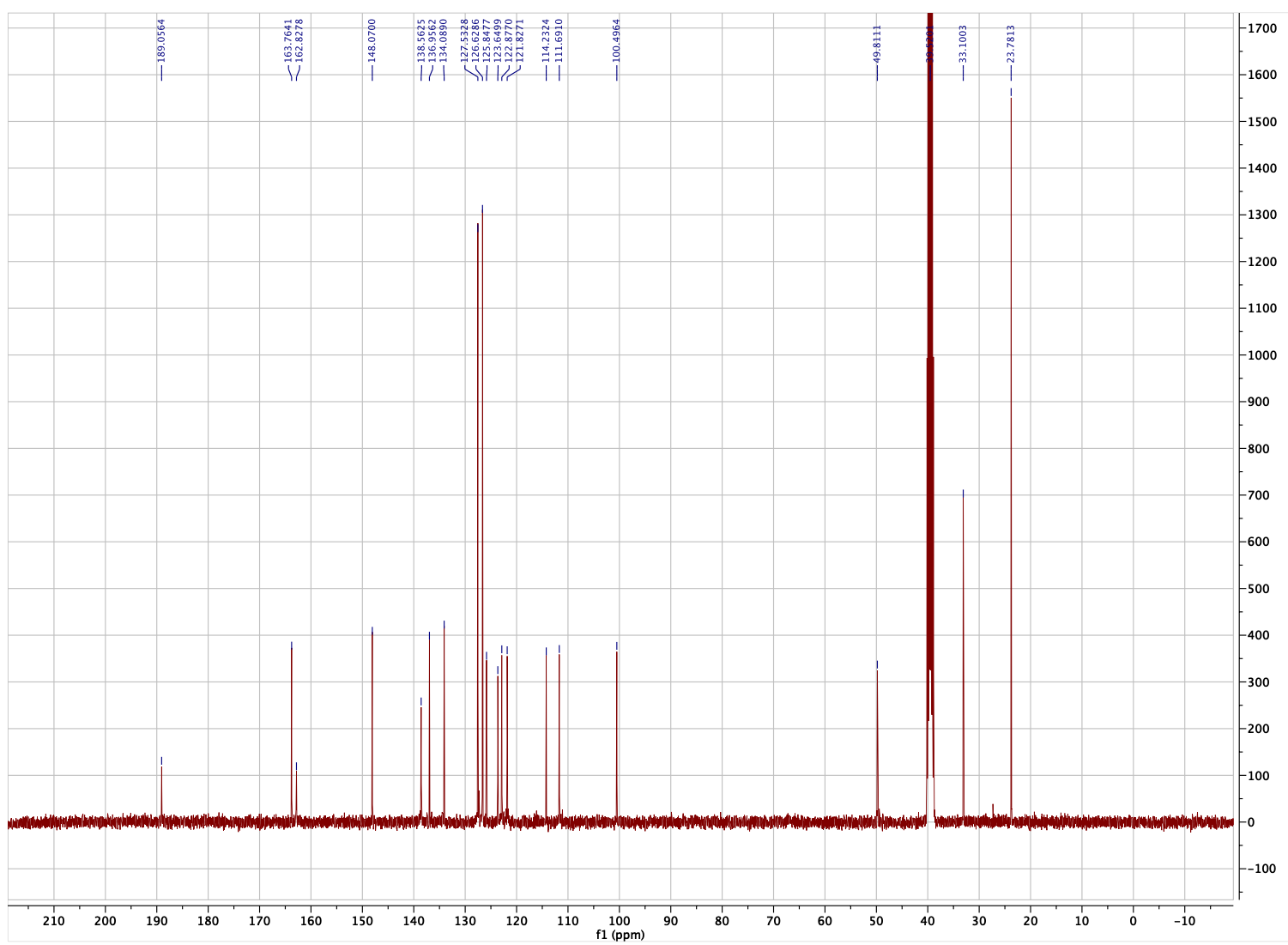


Figure S46. ^13^C NMR Spectrum for compound **7c**
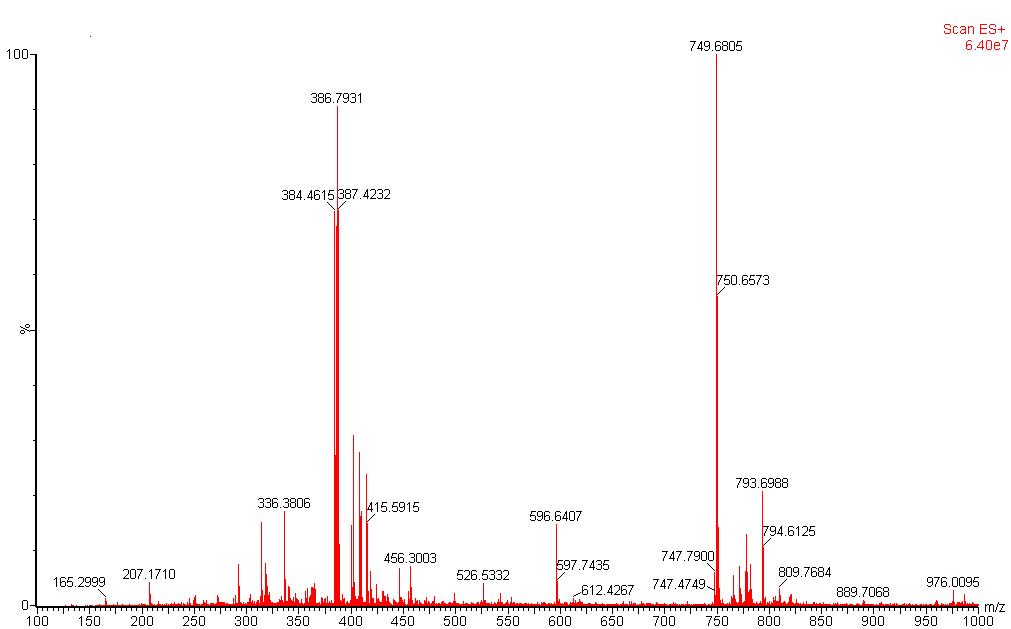


Figure S47. MS (ESI) Spectrum for compound **7c**


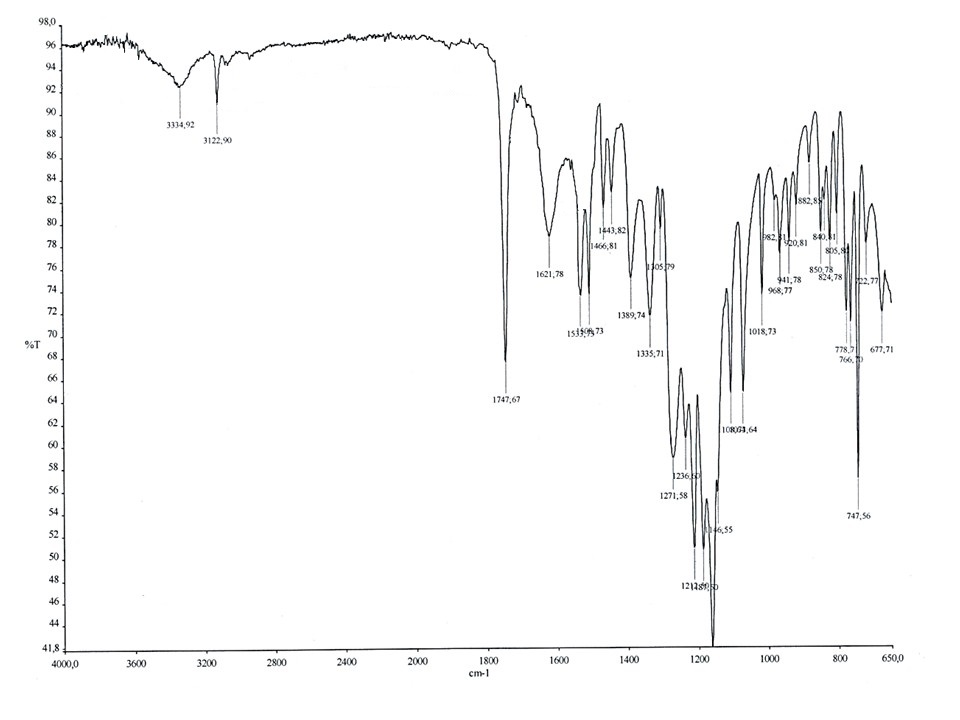
Figure S48. FTIR Spectrum for compound **7e**


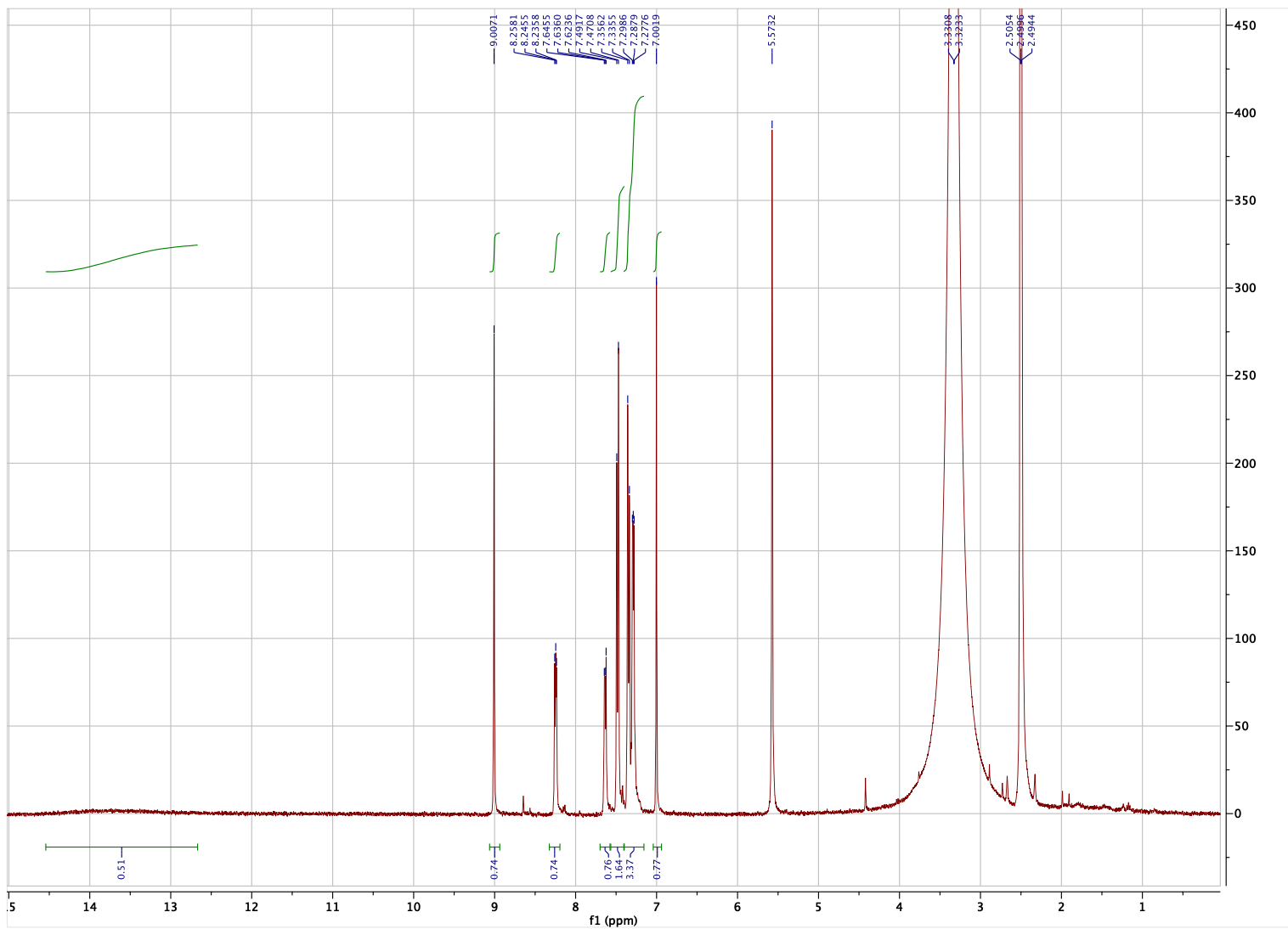
Figure S49. ^1^H NMR Spectrum for compound **7e**


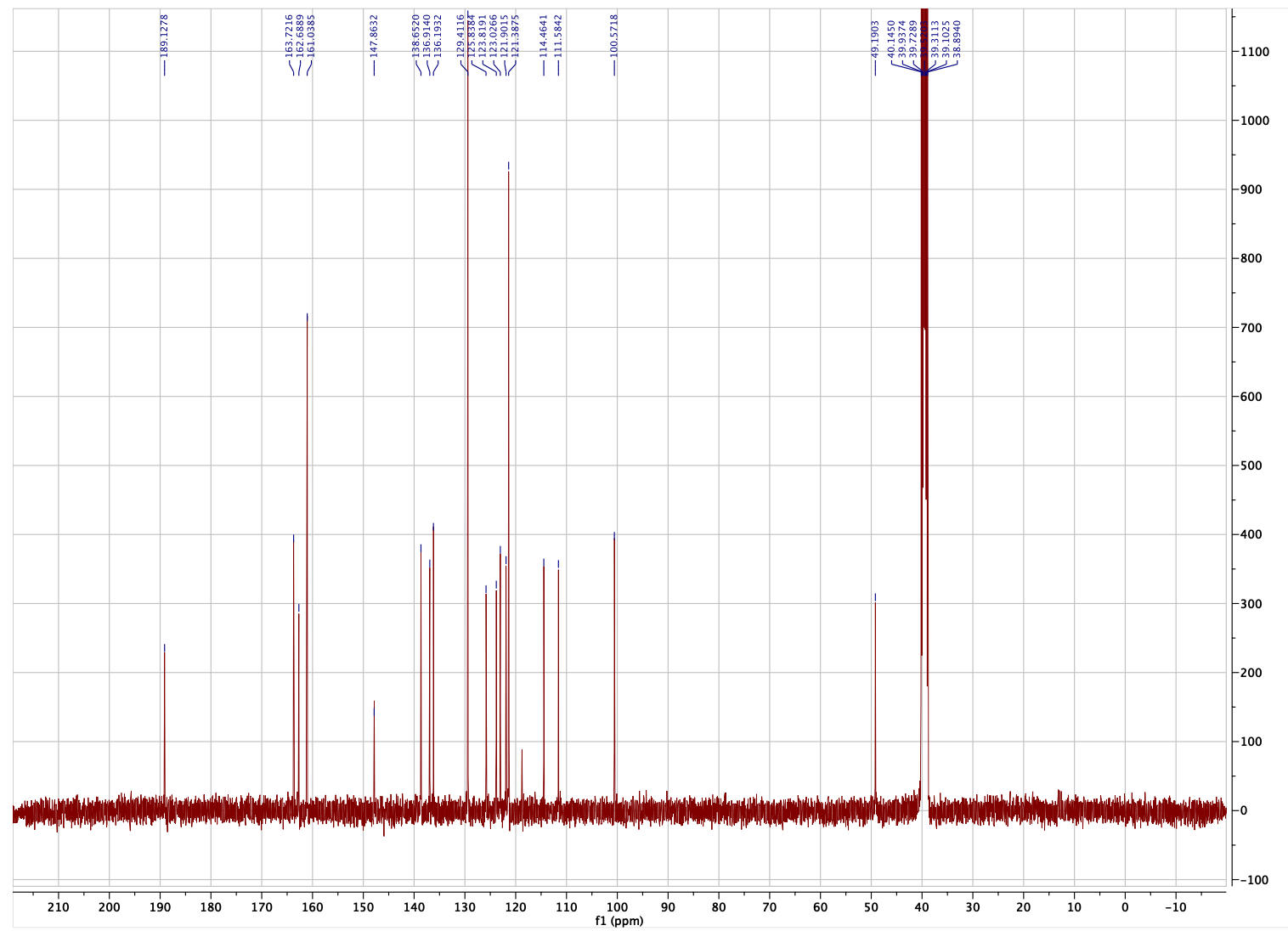


Figure S50. ^13^C NMR Spectrum for compound **7e**


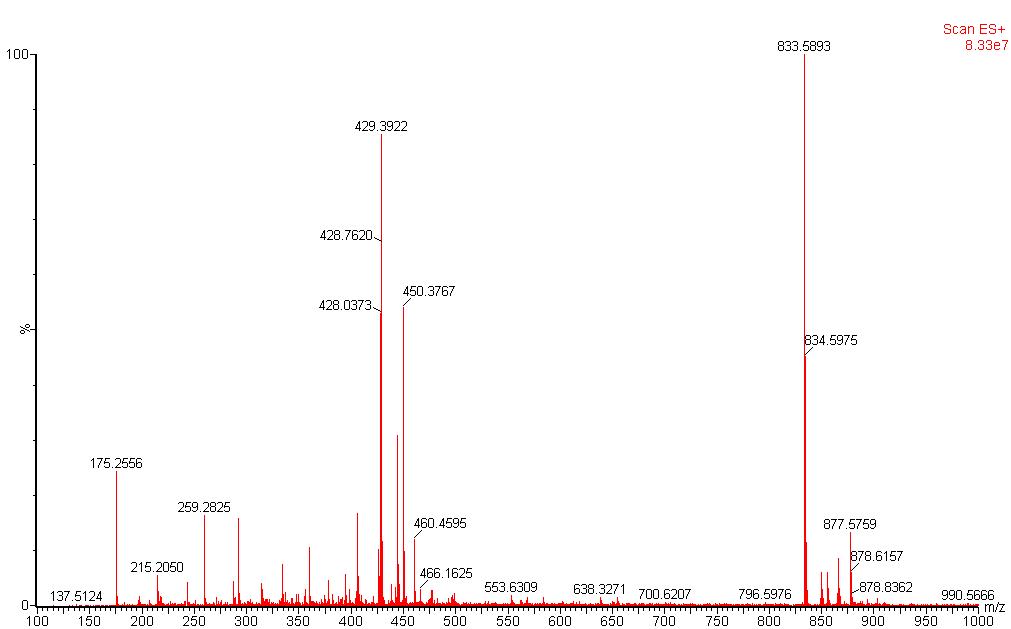


Figure S51. MS (ESI) Spectrum for compound **7e**


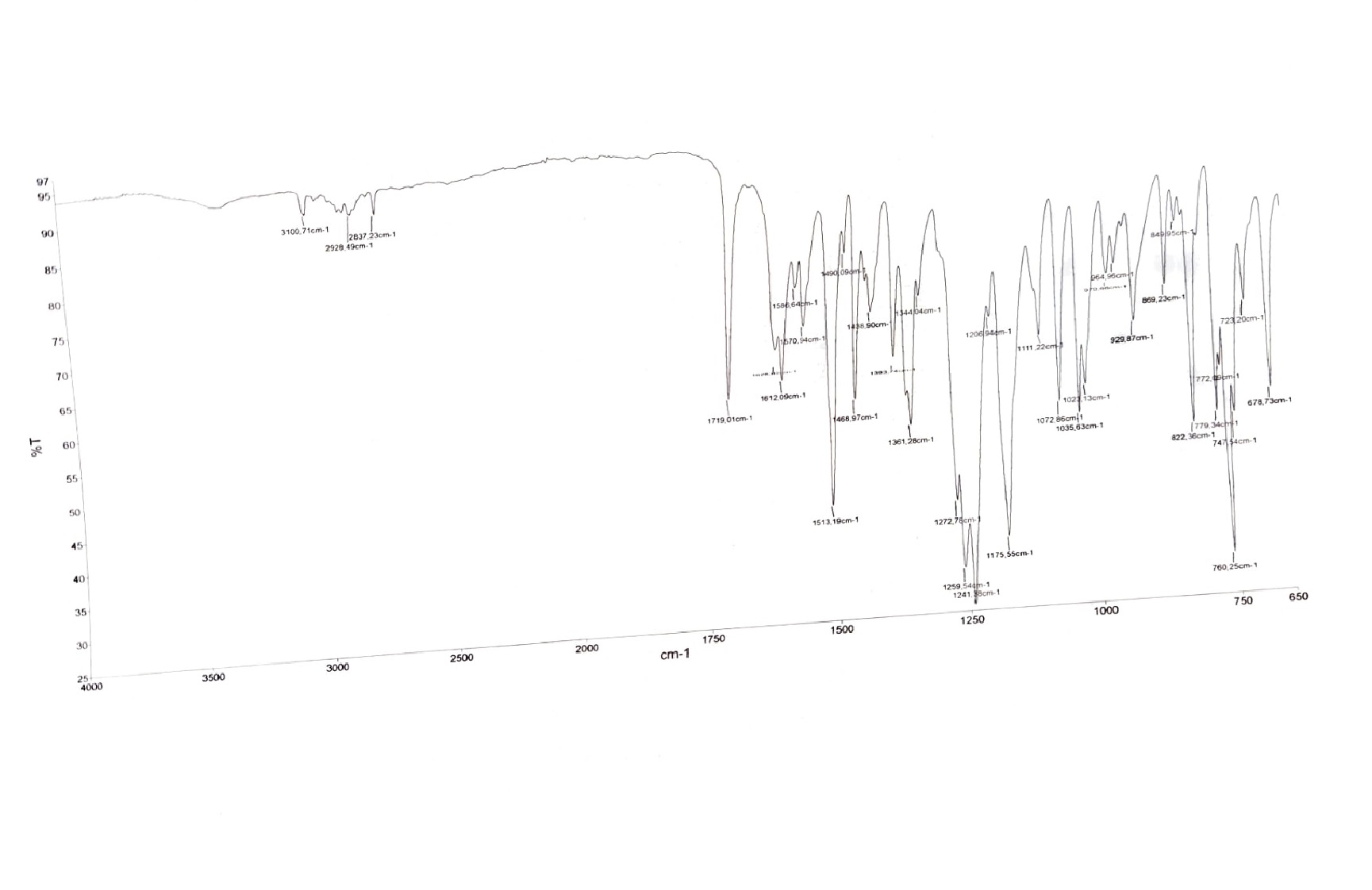


Figure S52. FTIR Spectrum for compound **8a**


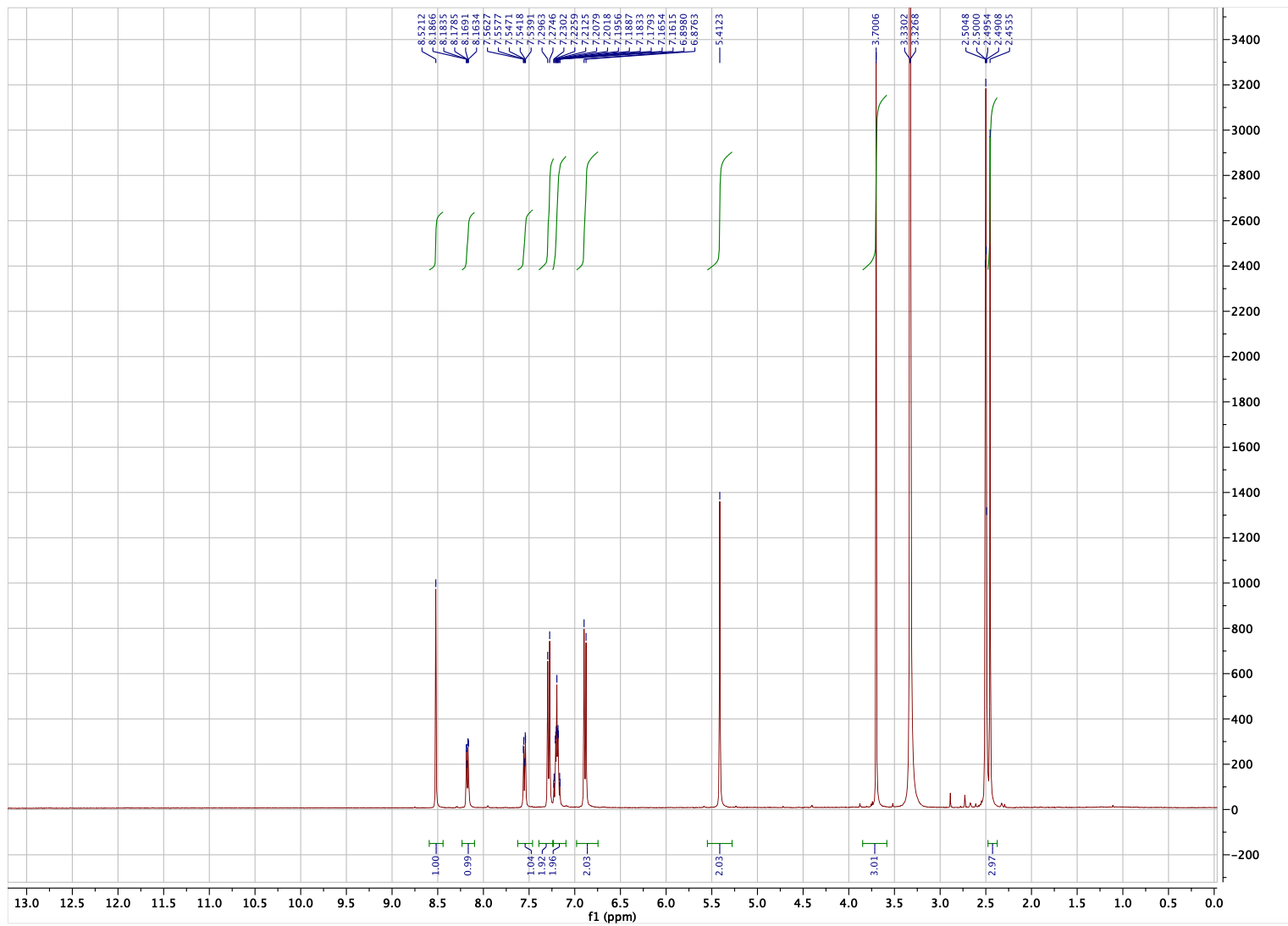


Figure S53. ^1^H NMR Spectrum for compound **8a**


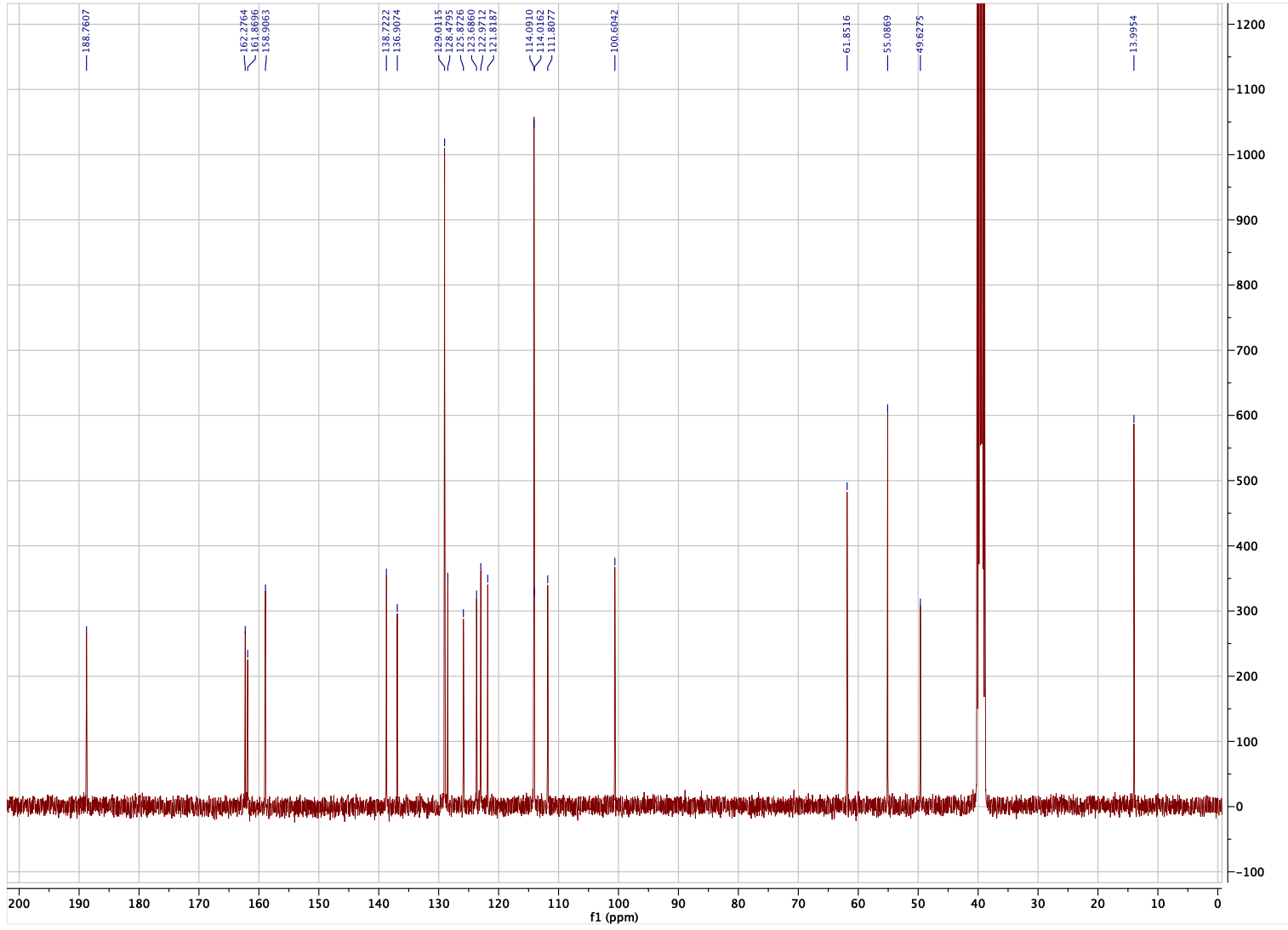
Figure S54. ^13^C NMR Spectrum for compound **8a**


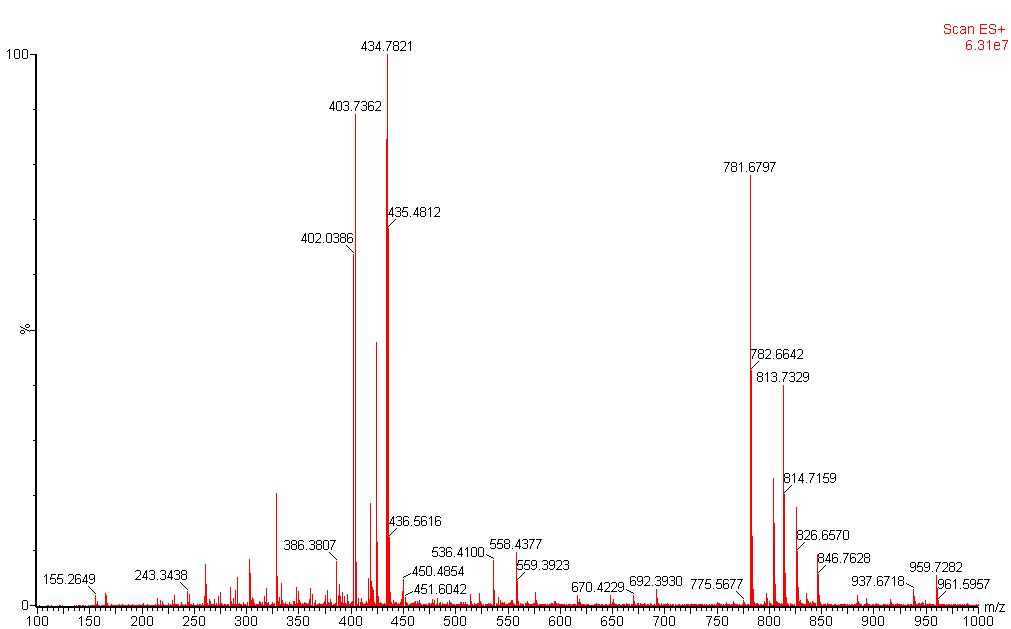


Figure S55. MS (ESI) Spectrum for compound **8a**


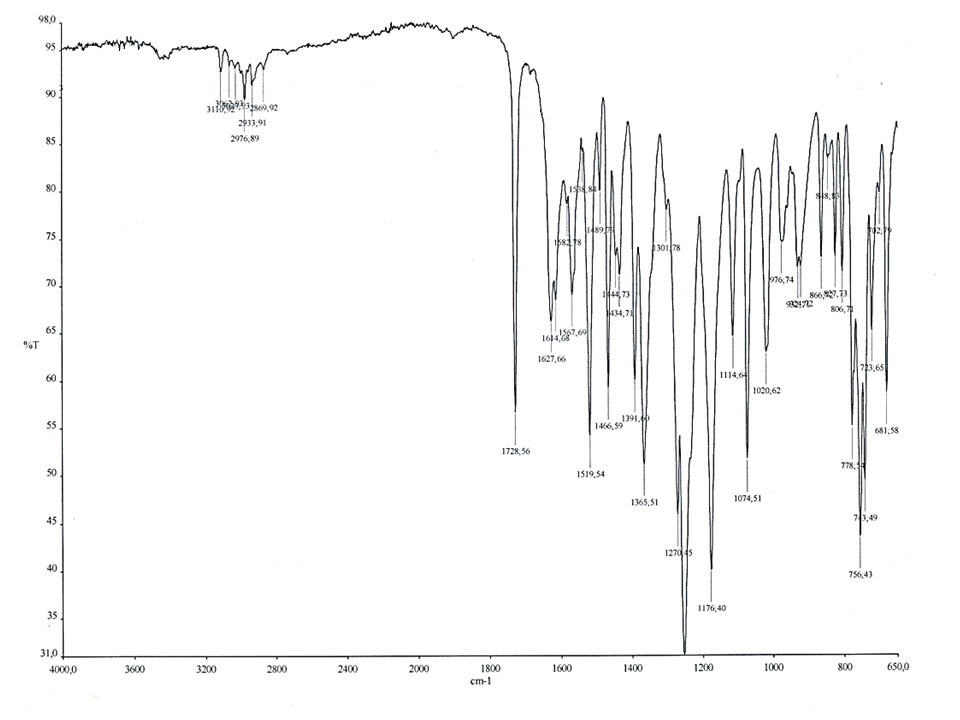


Figure S56. FTIR Spectrum for compound **8b**


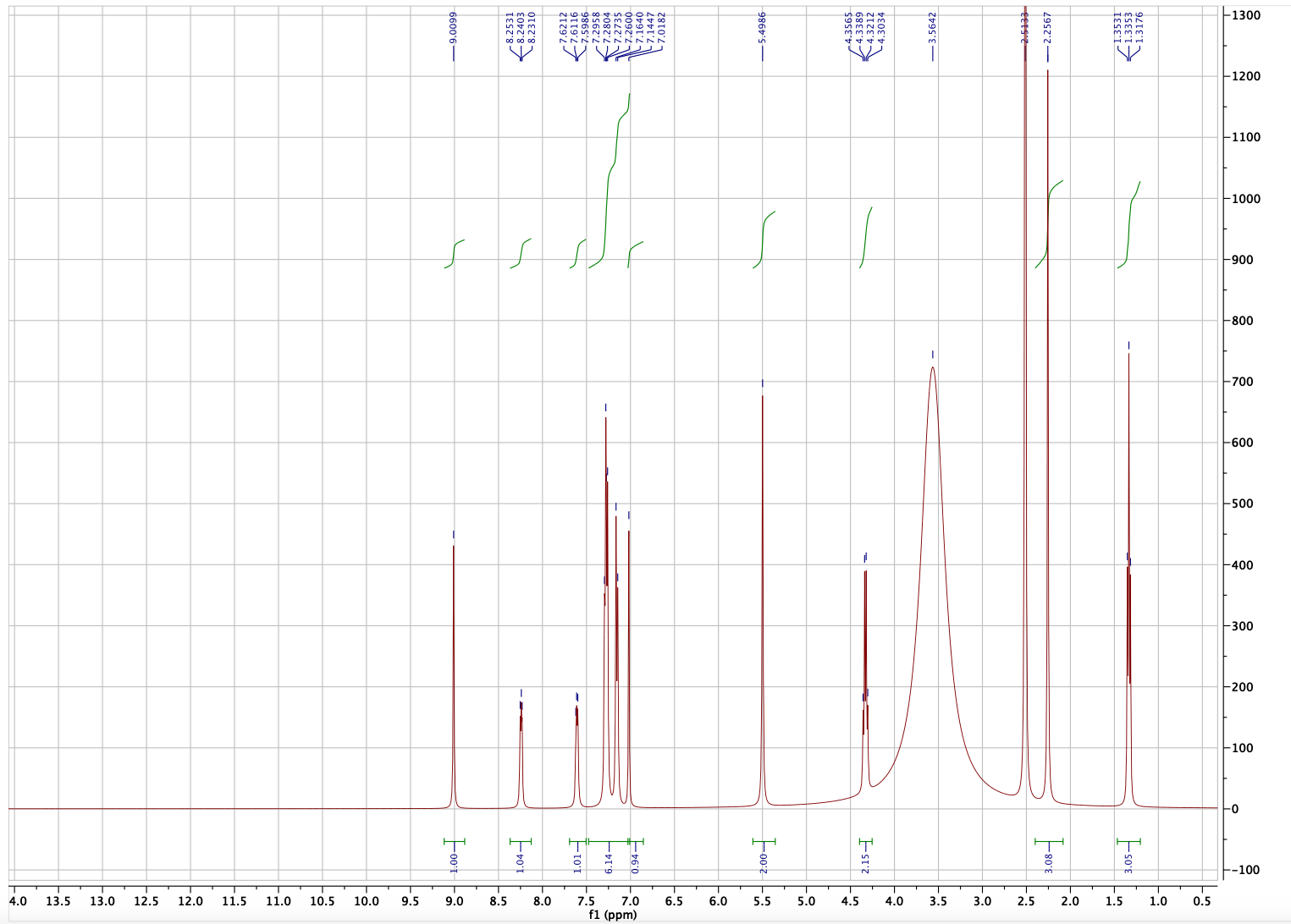


Figure S57. ^1^H NMR Spectrum for compound **8b**


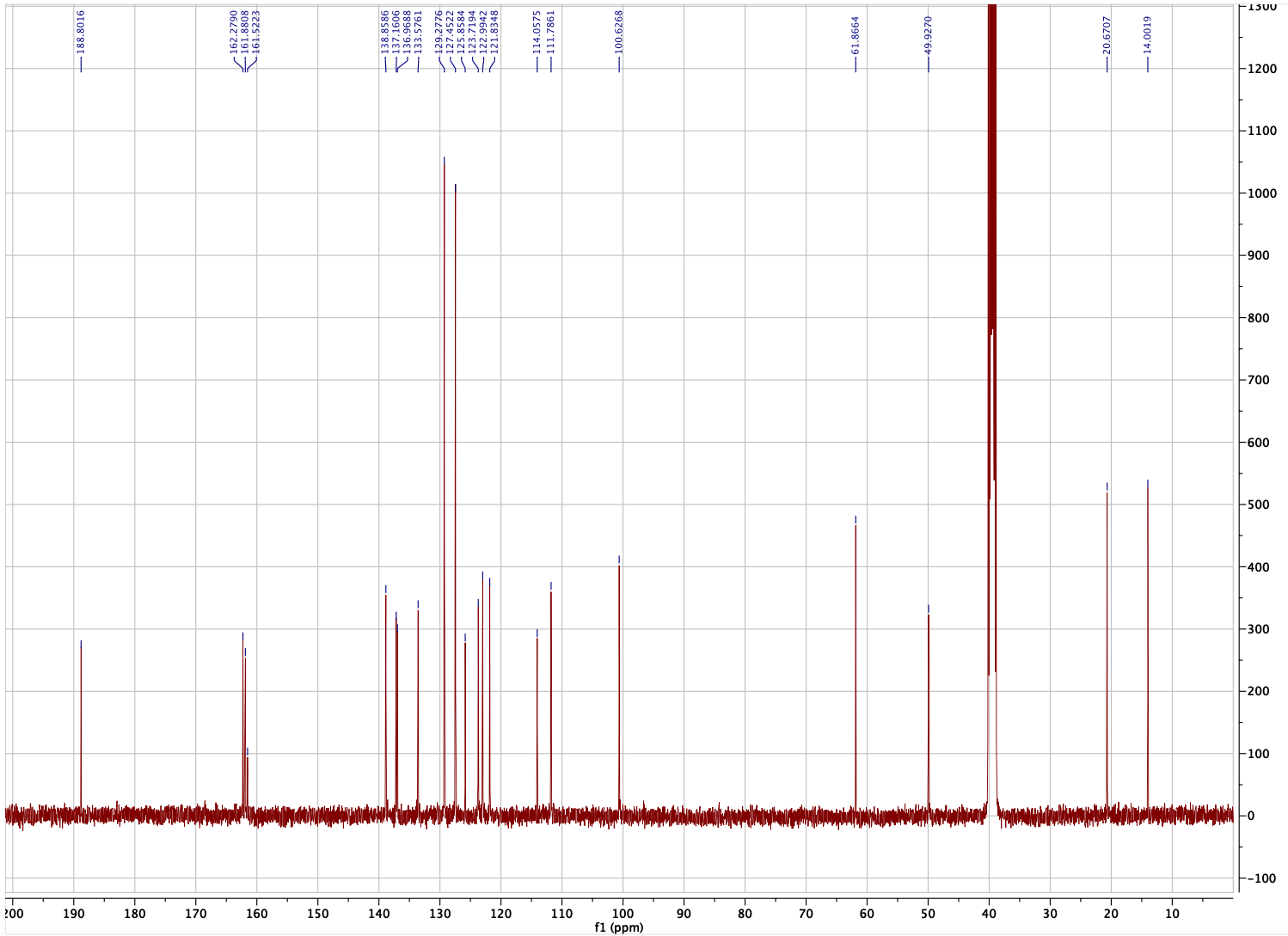


Figure S58. ^13^C NMR Spectrum for compound **8b**


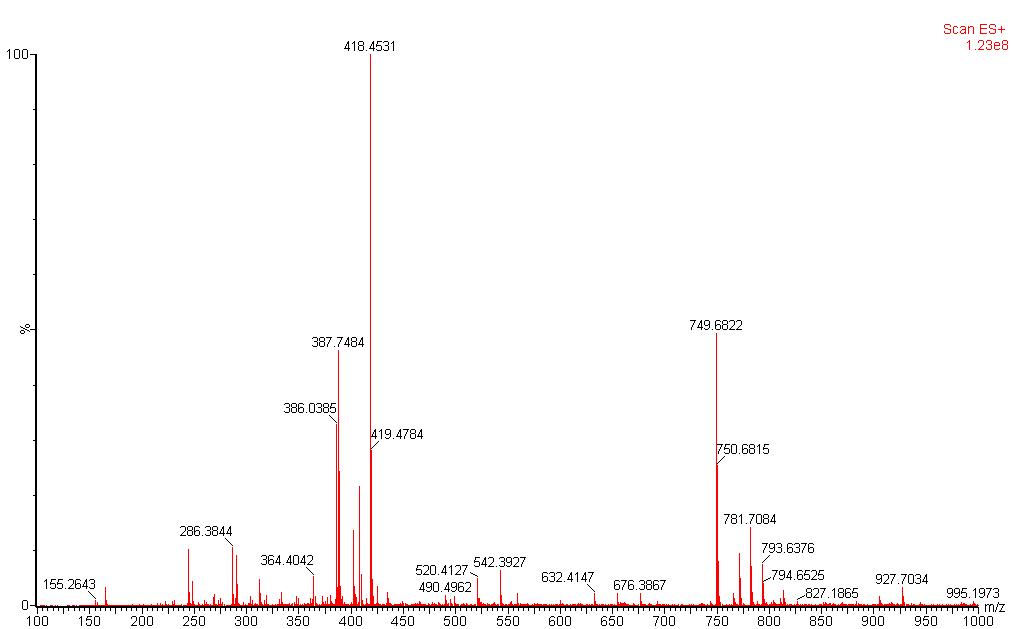


Figure S59. MS (ESI) Spectrum for compound **8b**


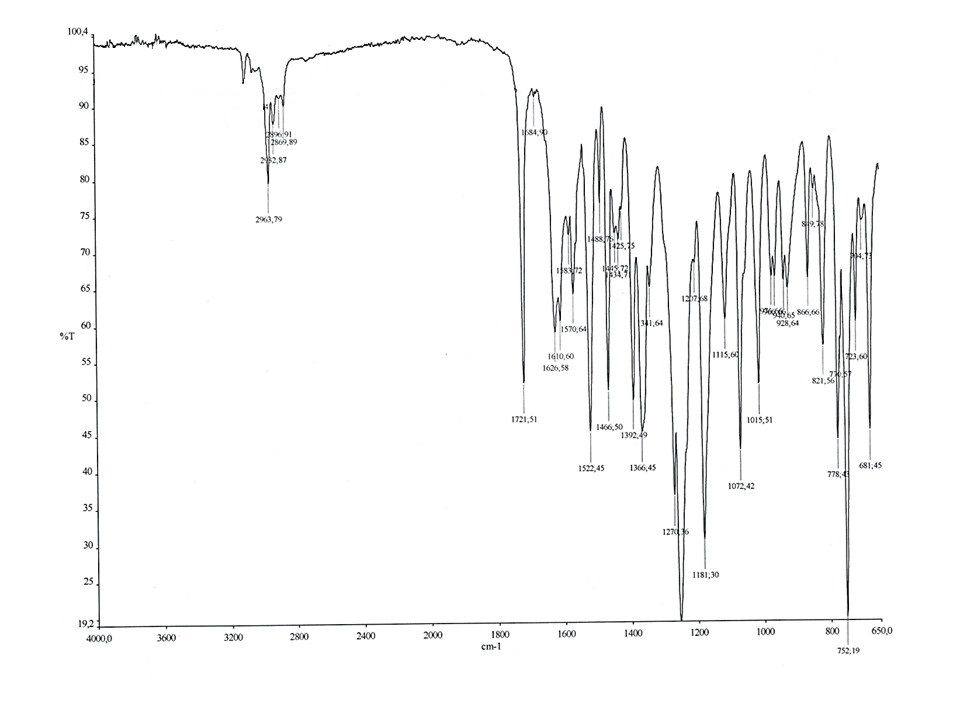


Figure S60. FTIR Spectrum for compound **8c**


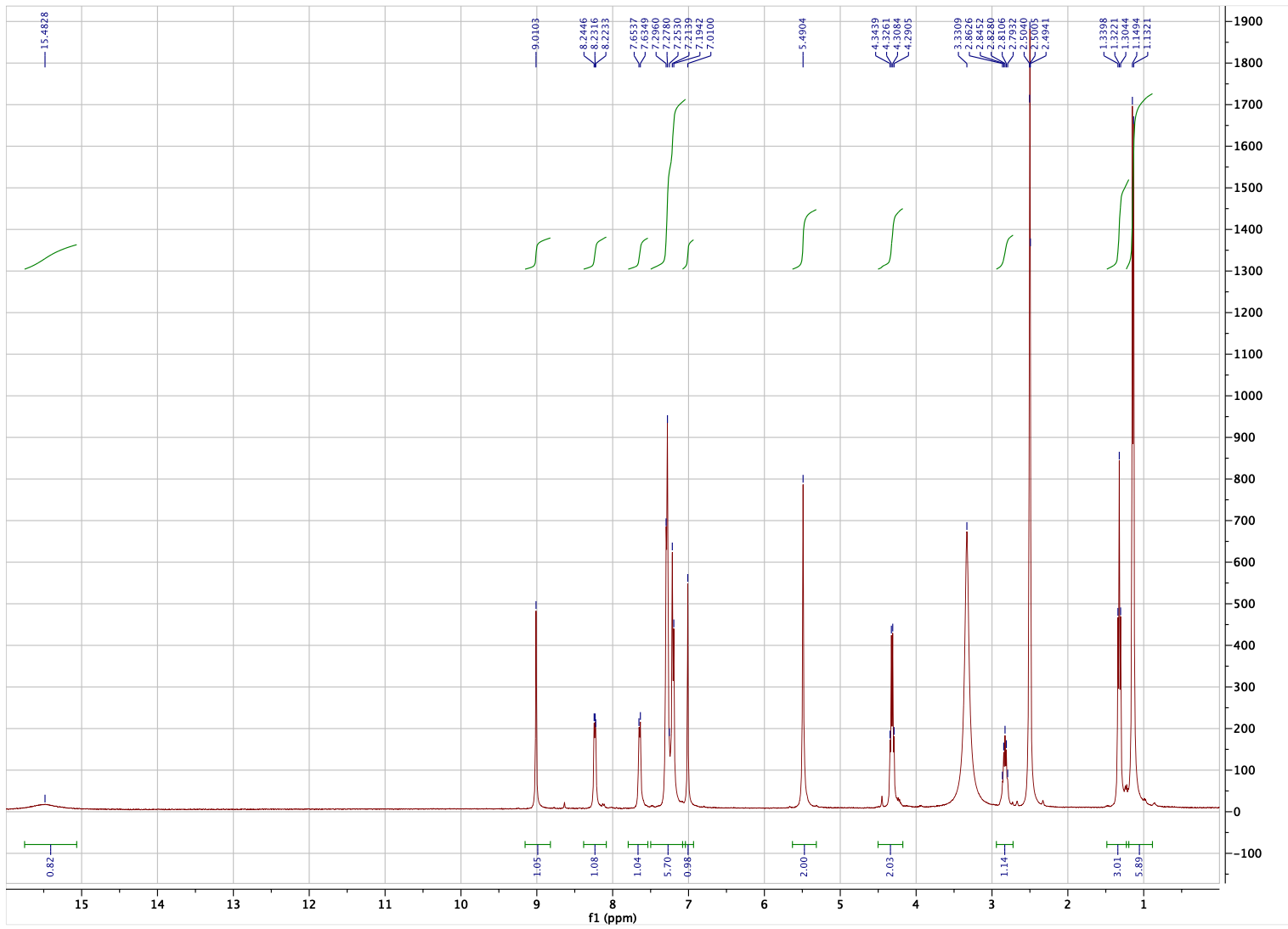


Figure S61. ^1^H NMR Spectrum for compound **8c**


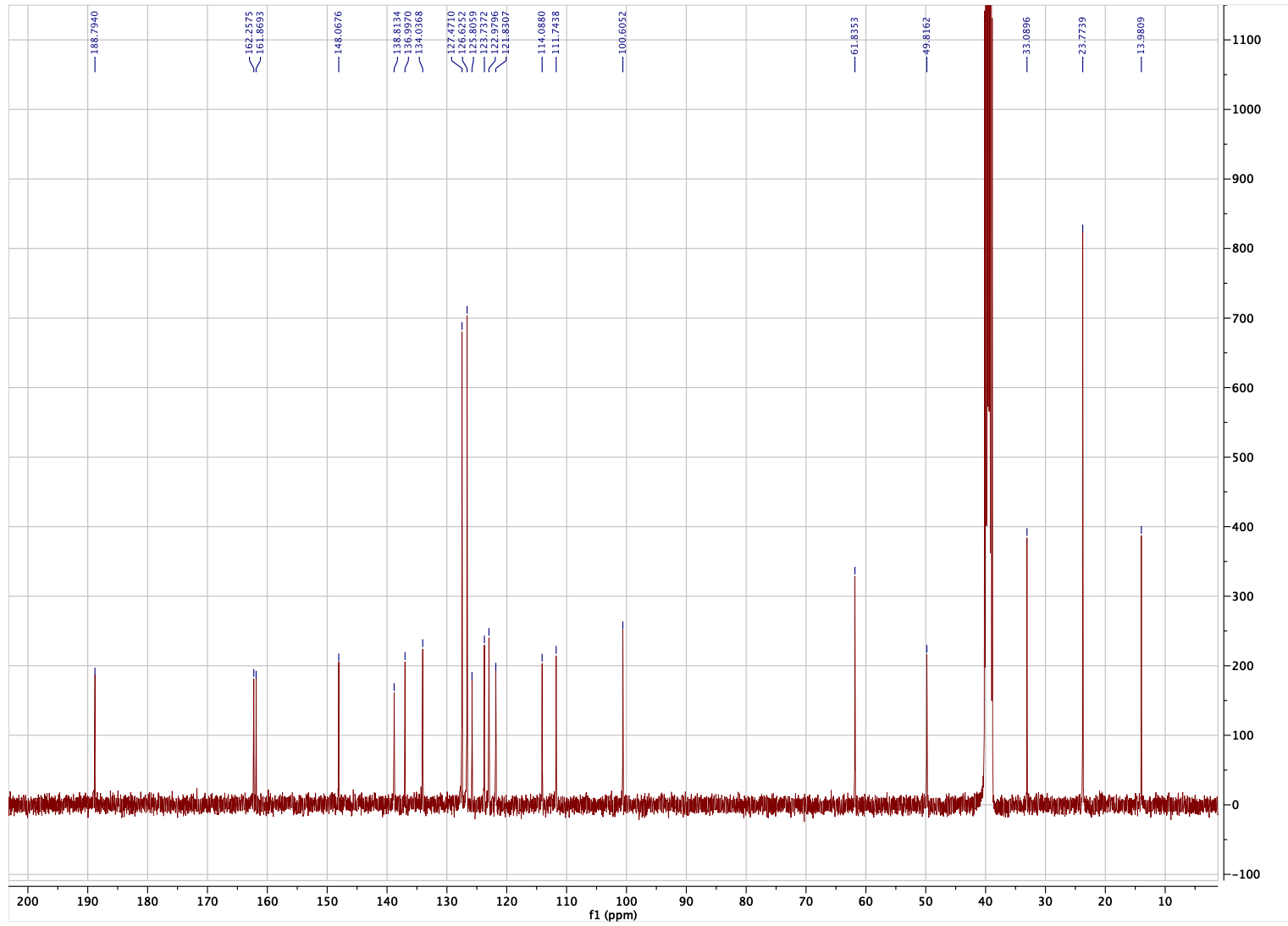
Figure S62. ^13^C NMR Spectrum for compound **8c**


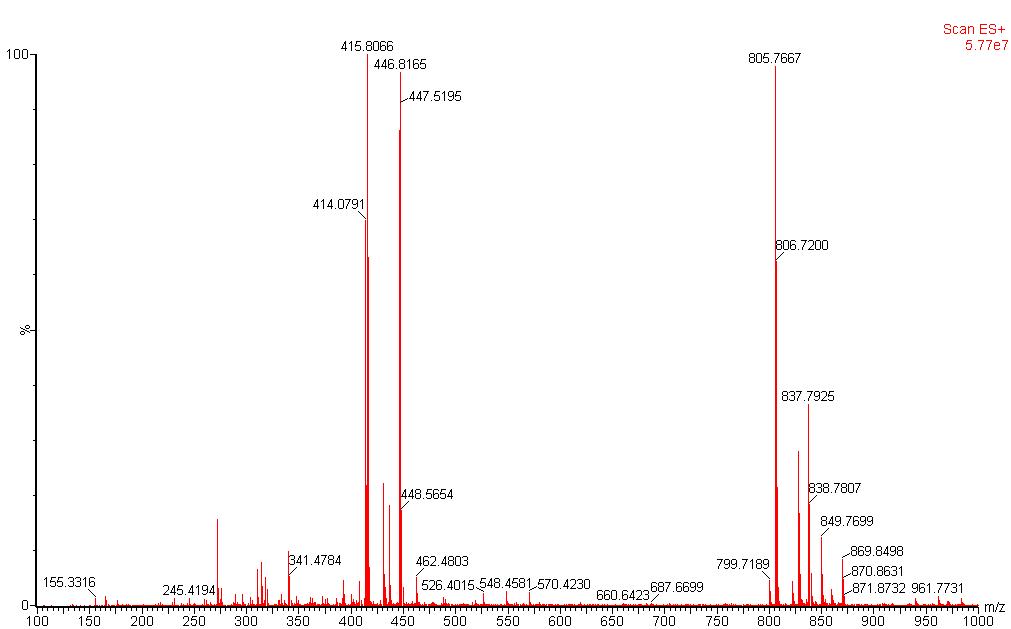


Figure S63. MS (ESI) Spectrum for compound **8c**


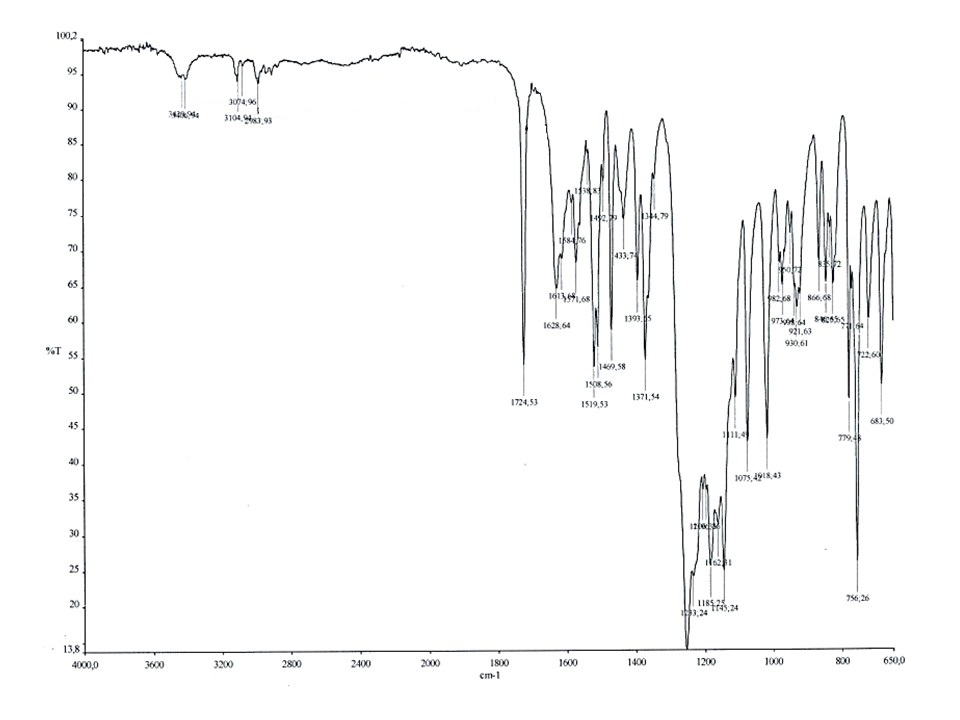


Figure S64. FTIR Spectrum for compound **8e**


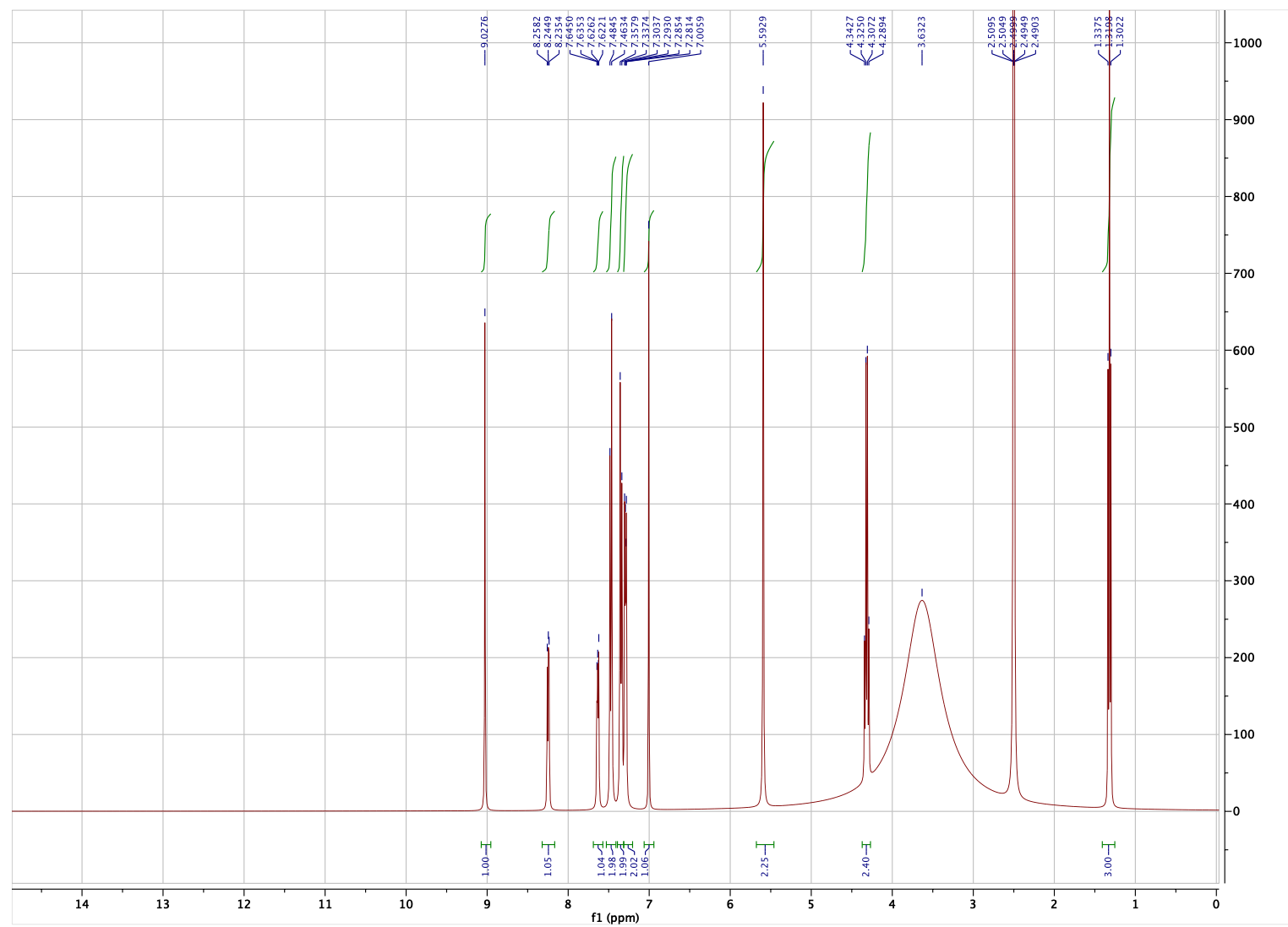
Figure S65. ^1^H NMR Spectrum for compound **8e**


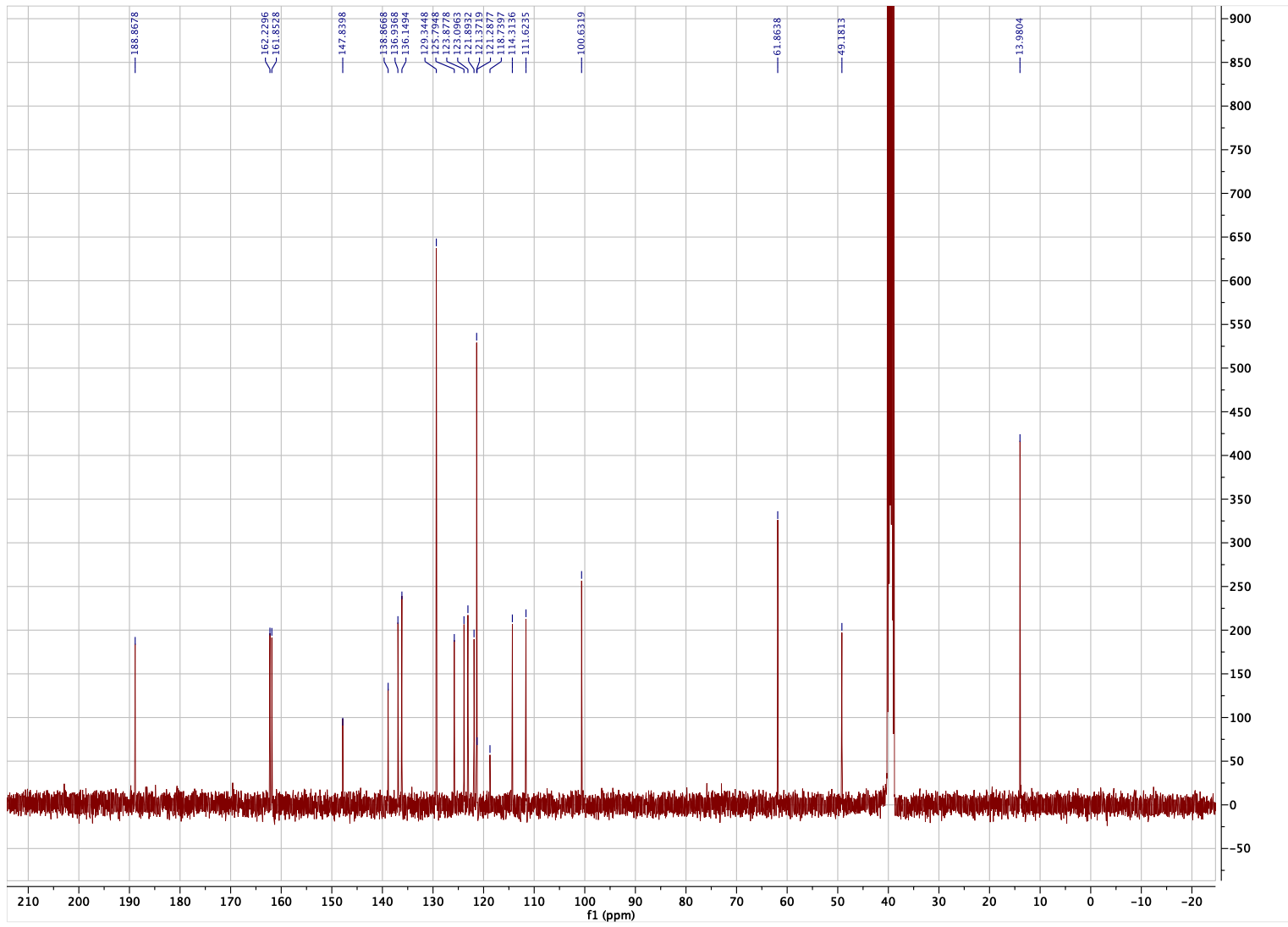
Figure S66. ^13^C NMR Spectrum for compound **8e**


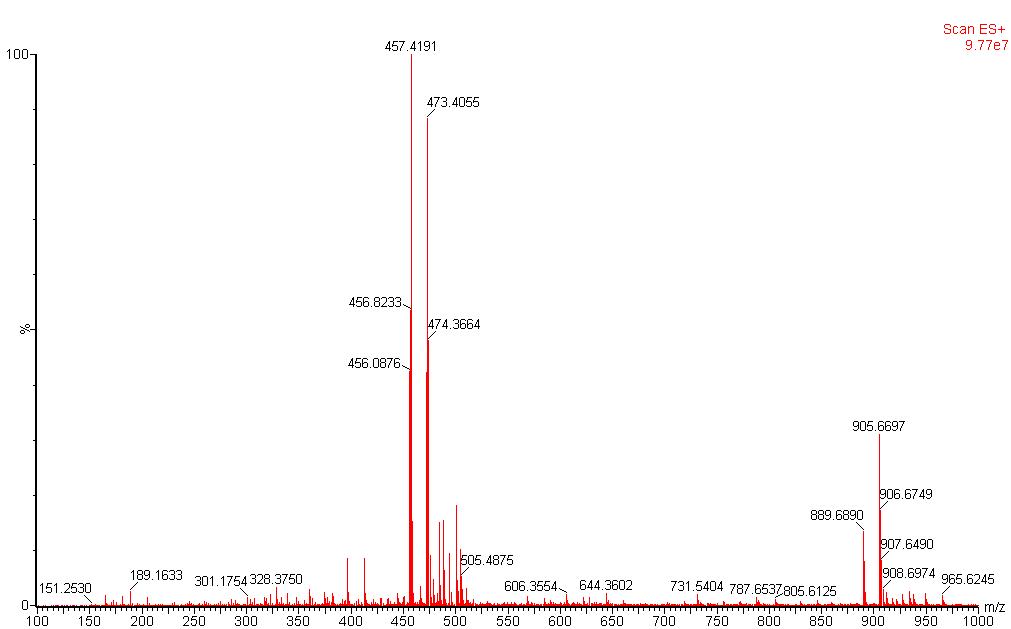


Figure S67. MS (ESI) Spectrum for compound **8e**


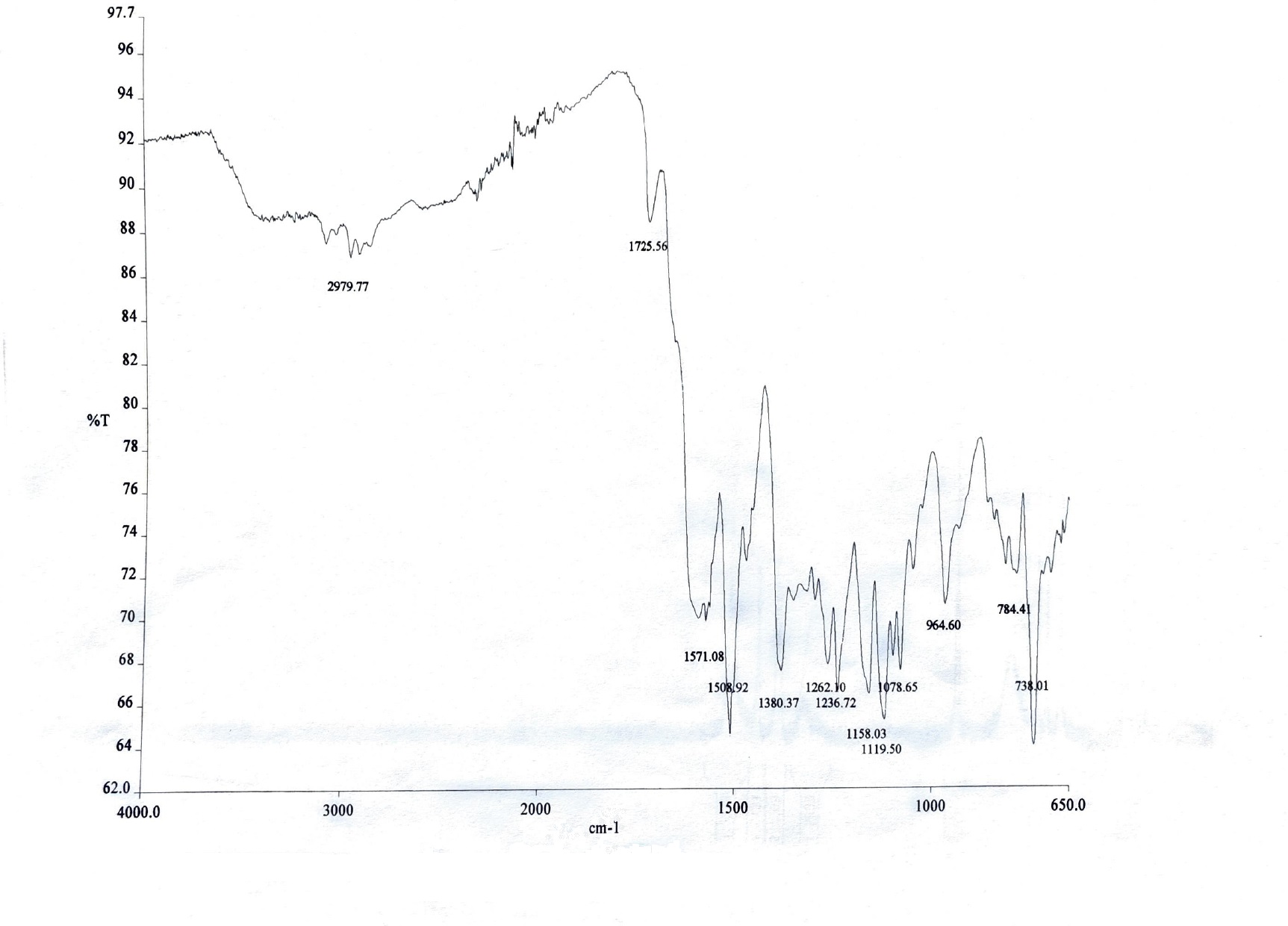


Figure S68. FTIR Spectrum for compound **9d**


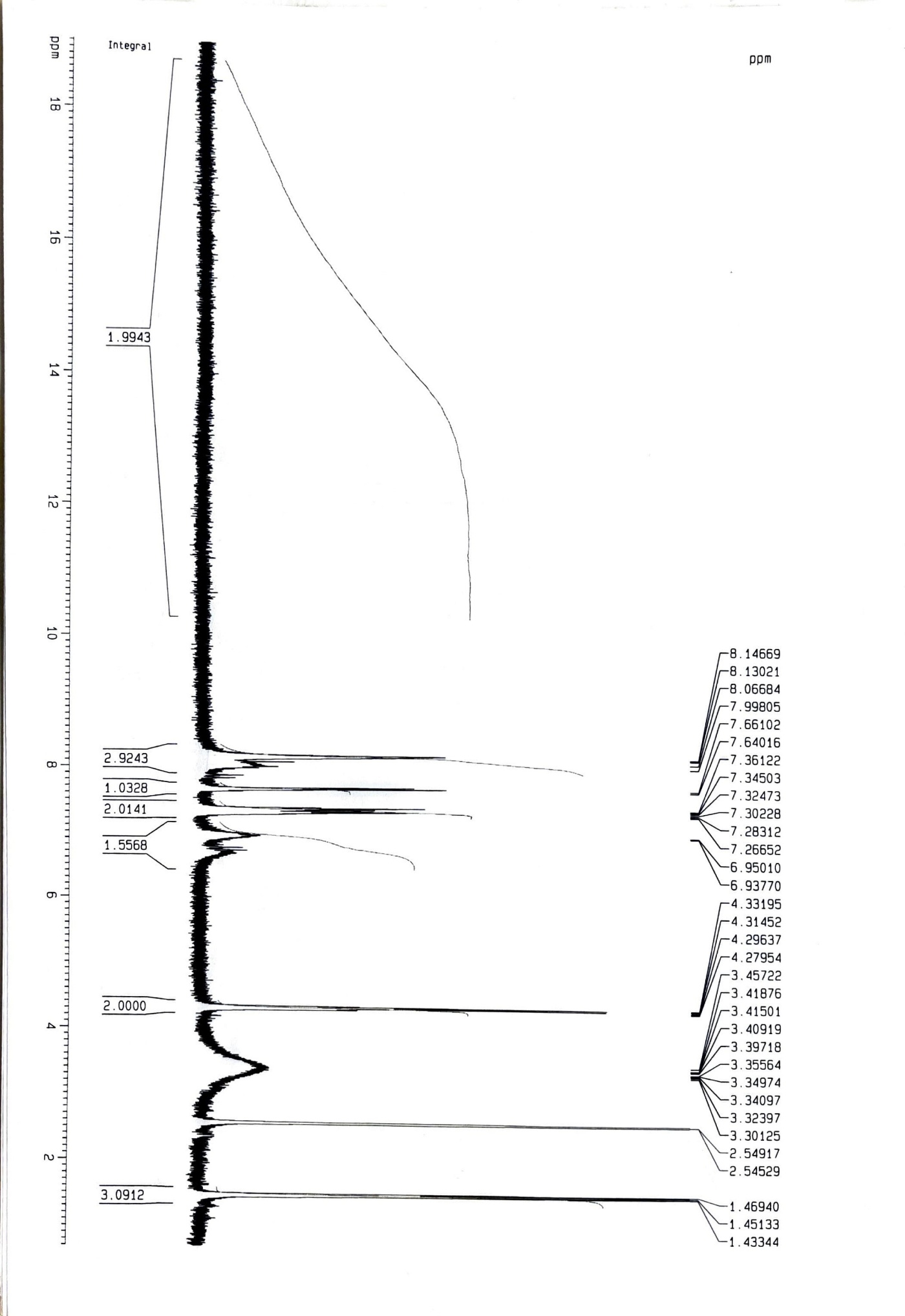


Figure S69. ^1^H NMR Spectrum for compound **9d**


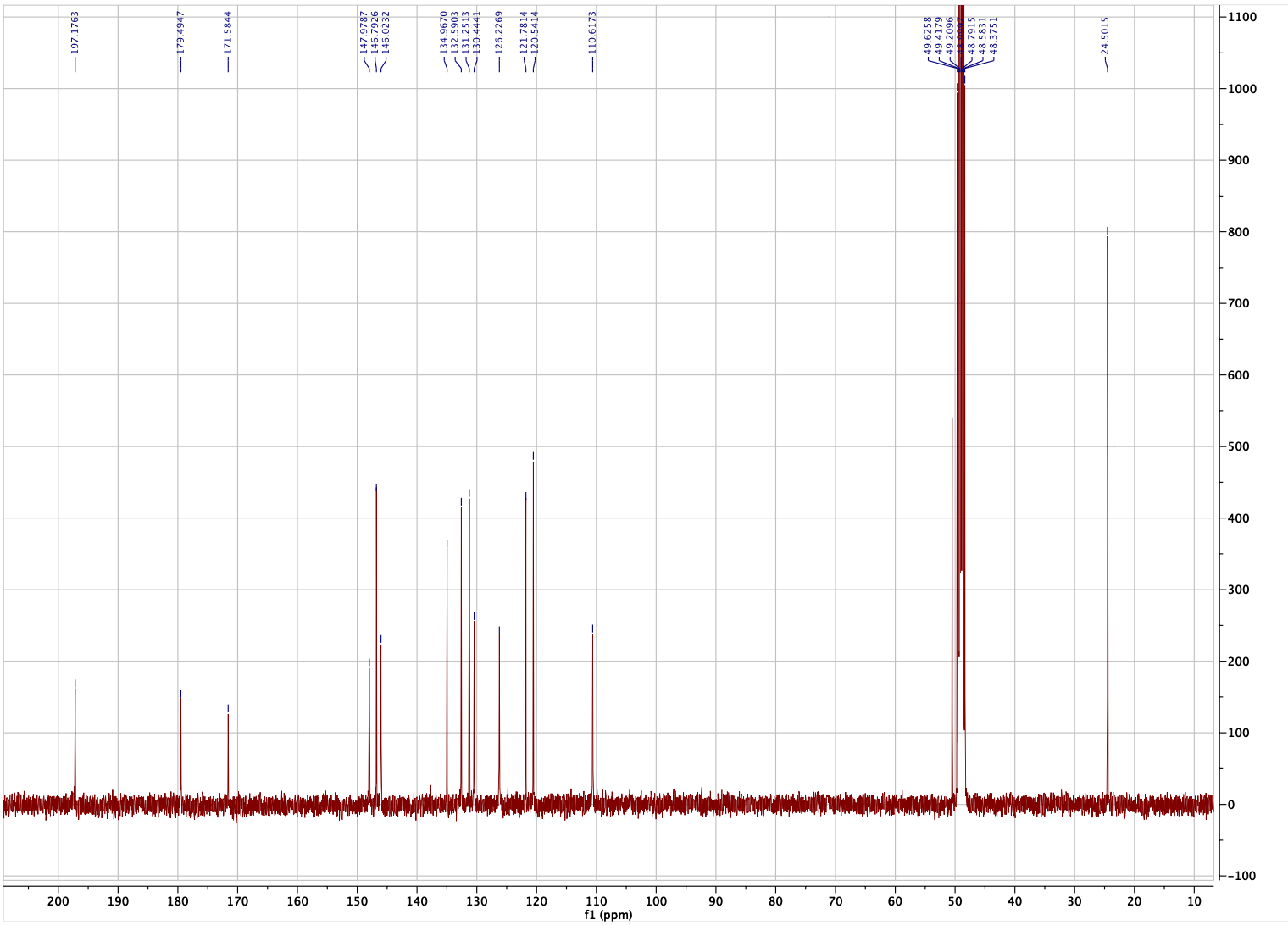


Figure S70. ^13^C NMR Spectrum for compound **9d**
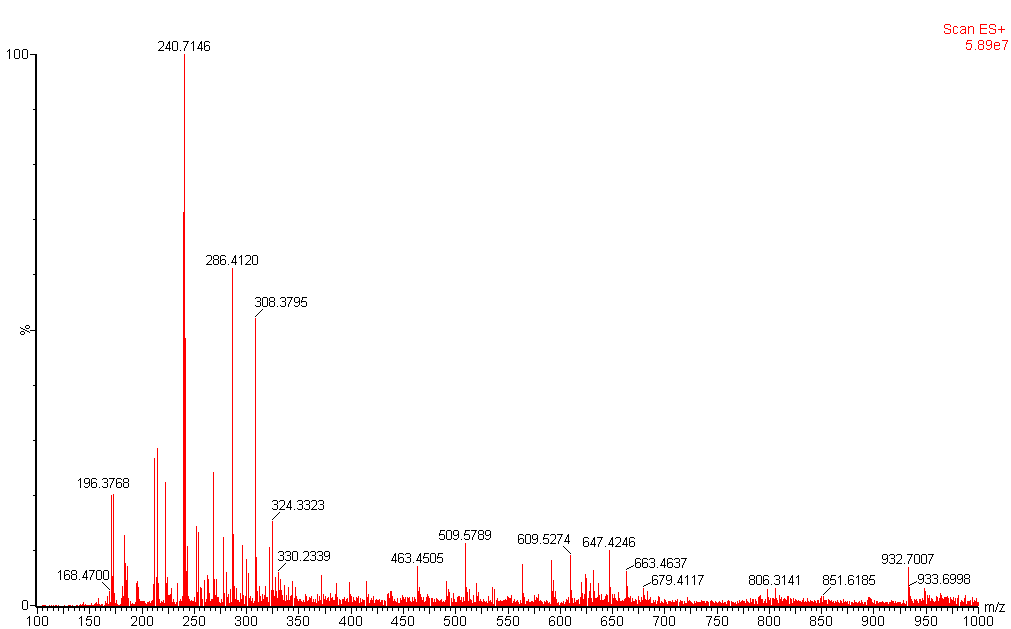


Figure S71. MS (ESI) Spectrum for compound **9d**


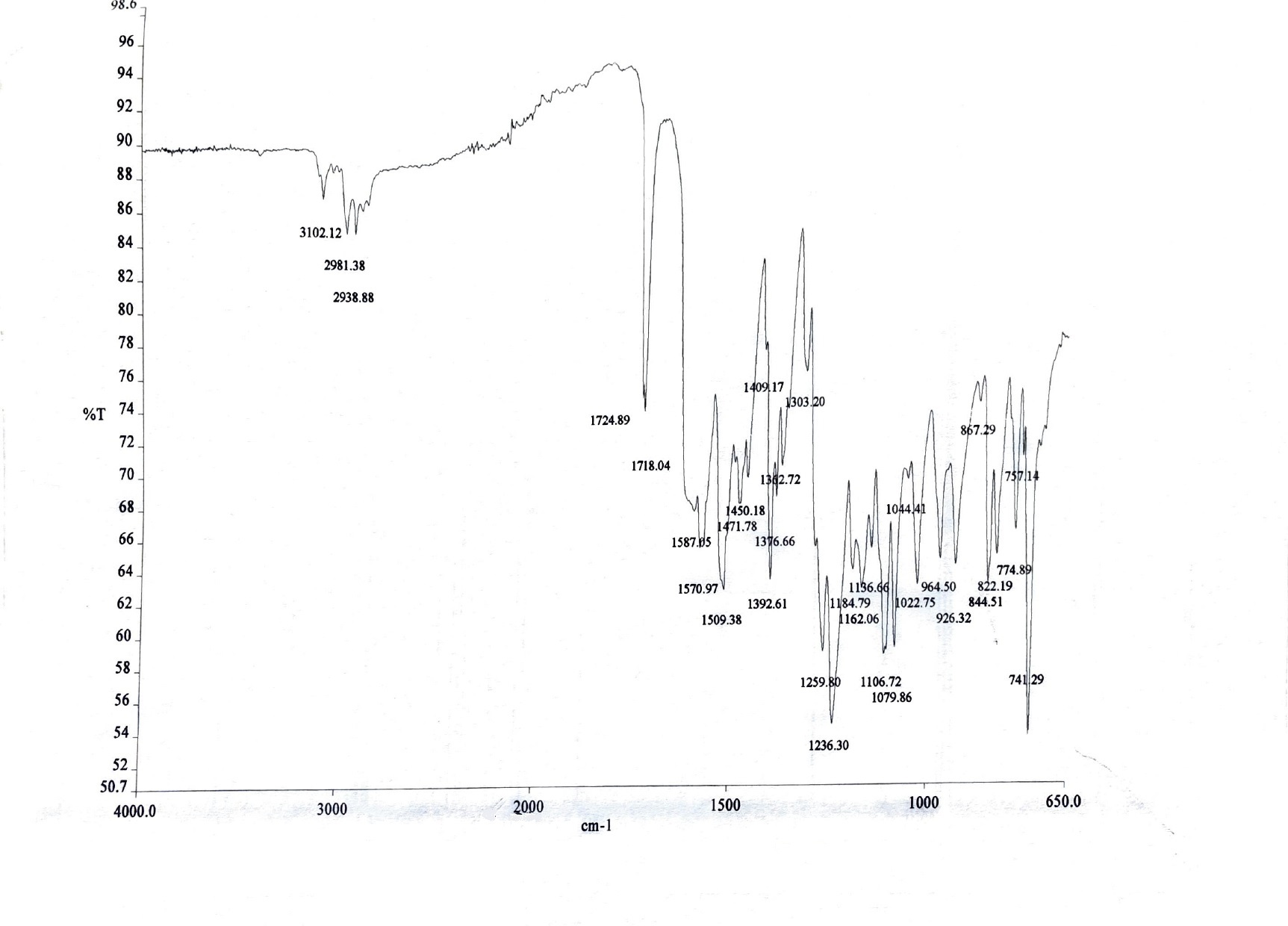


Figure S72. FTIR Spectrum for compound **10d**


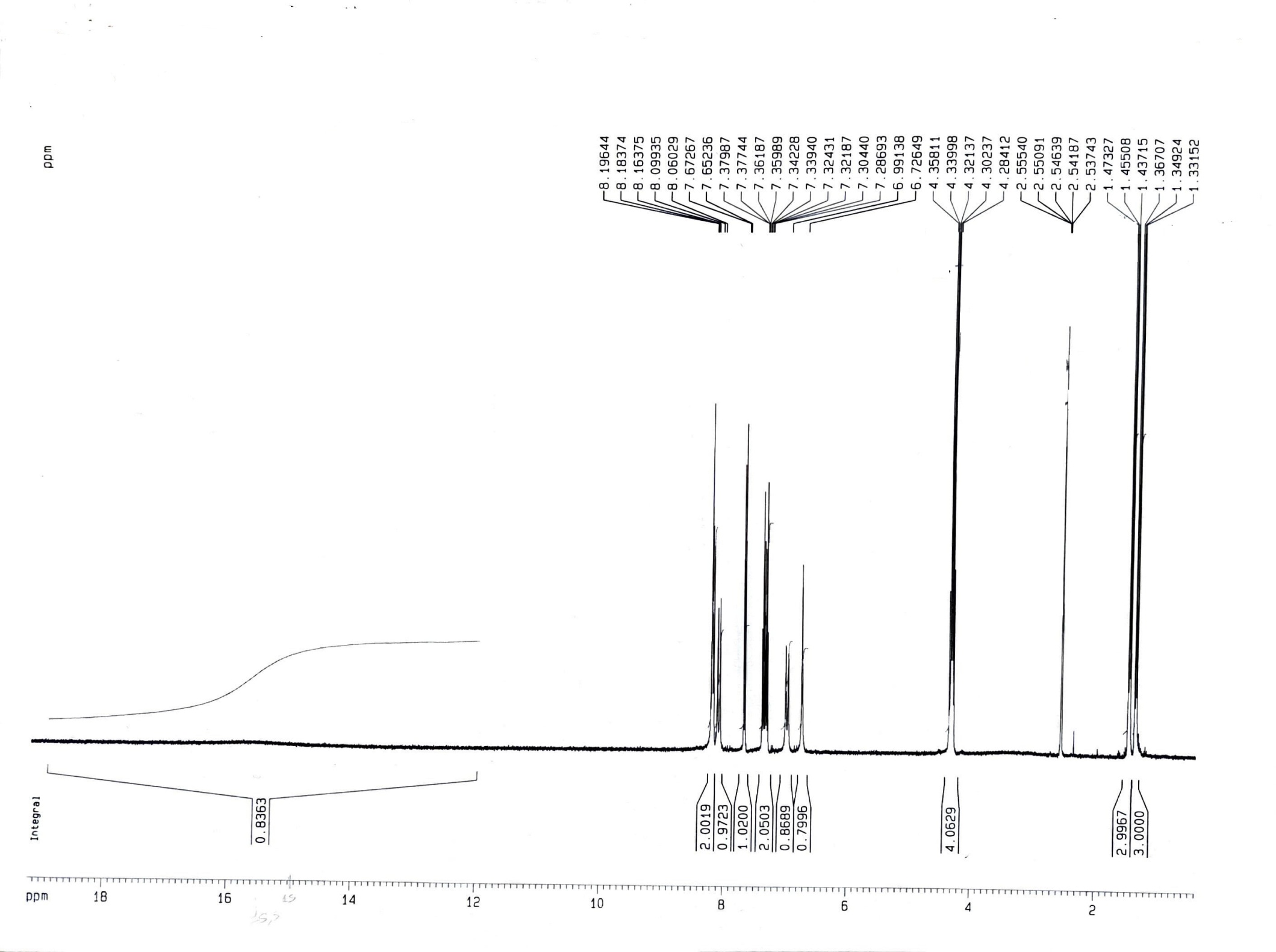


Figure S73. ^1^H NMR Spectrum for compound **10d**


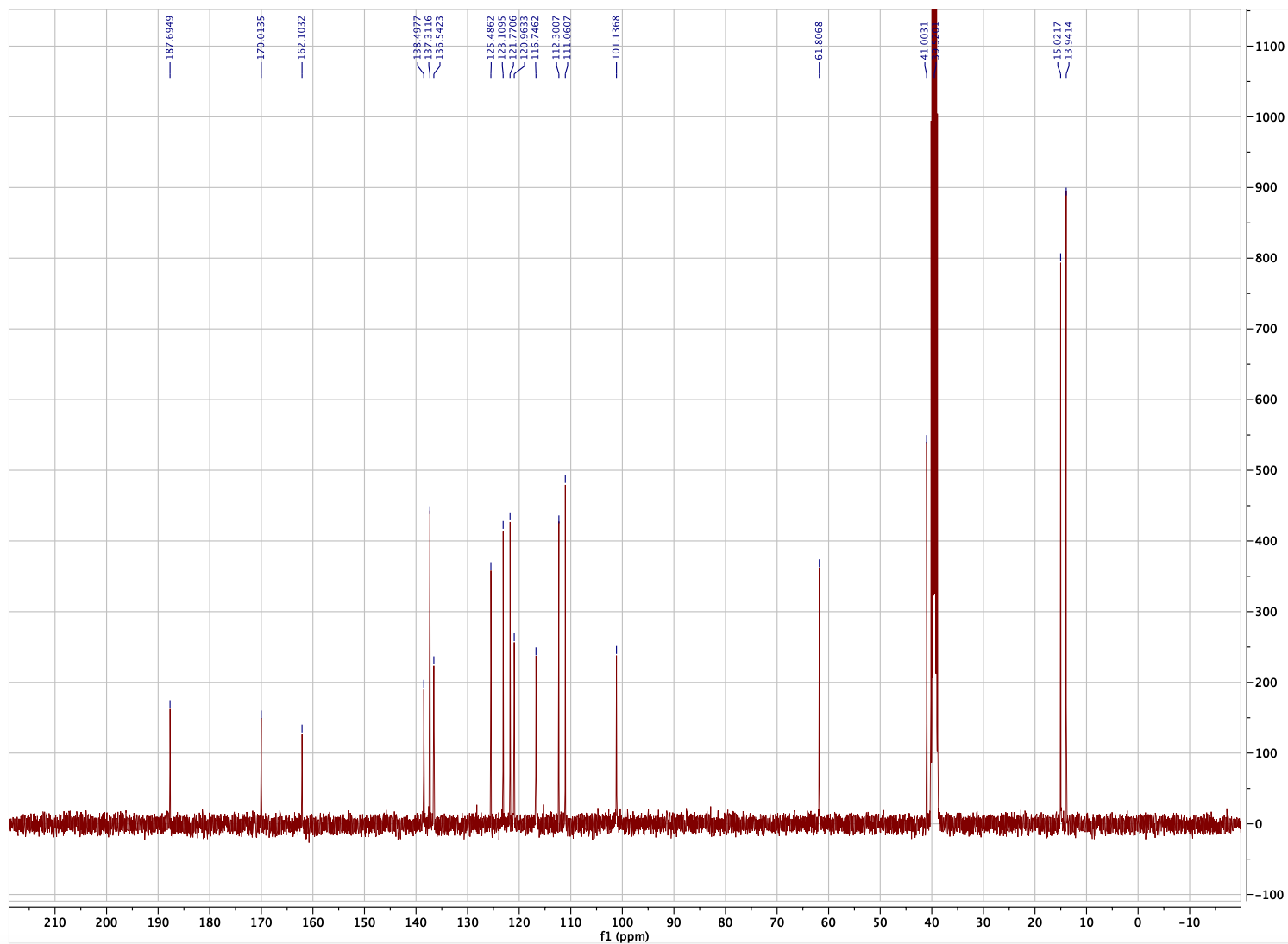


Figure S74. ^13^C NMR Spectrum for compound **10d**


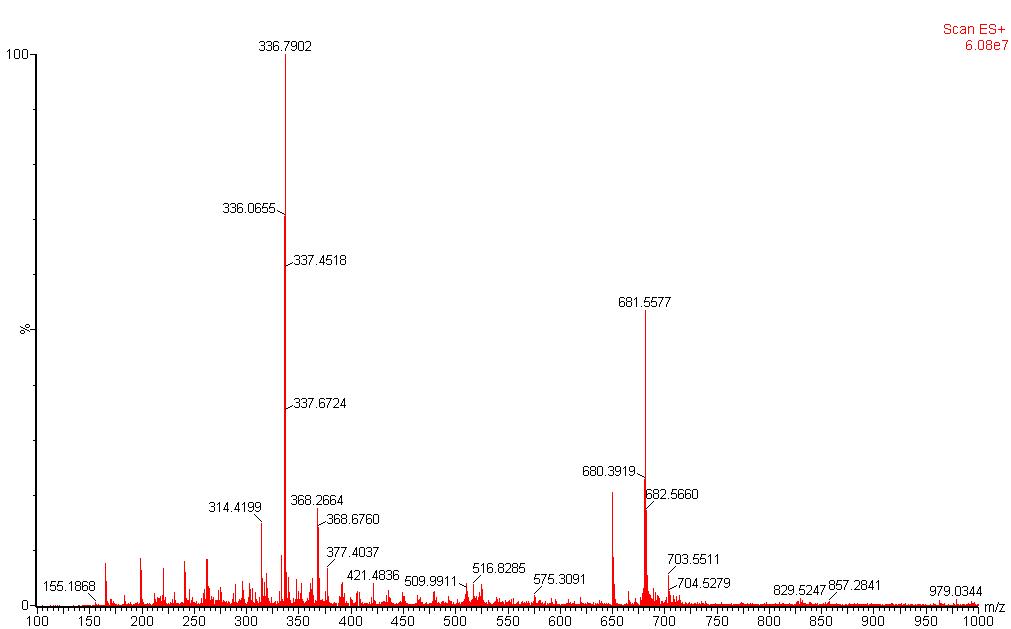


Figure S75. MS (ESI) Spectrum for compound **10d**
